# CCL26–CX3CR1 Axis Mediates a Feedback Loop between Cancer Cell and PMN-MDSCs to Promote CD8^+^ T Cell Exhaustion during Stomach Carcinogenesis

**DOI:** 10.34133/research.1002

**Published:** 2025-11-21

**Authors:** Xiaotao Jiang, Hui Wu, Ning Yan, Anzhou Wu, Xianzhe Wang, Yi Wen, Jinqi An, Jingming Chen, Jiaxing Yan, Changlong Wang, Yushan Zou, Yuancheng Huang, Wei Wang, Peiwu Li, Kunhai Zhuang, Yufeng Liu, Fengbin Liu

**Affiliations:** ^1^ First Clinical Medical College, Guangzhou University of Chinese Medicine, Guangzhou 510405, Guangdong, China.; ^2^ Lingnan Medical Research Center, Guangzhou University of Chinese Medicine, Guangzhou 510405, Guangdong, China.; ^3^ The First Affiliated Hospital of Guangzhou University of Chinese Medicine, Guangzhou 510405, Guangdong, China.; ^4^ Department of Oncology, Dongguan People’s Hospital (The Tenth Affiliated Hospital of Southern Medical University), Dongguan 523000, Guangdong, China.; ^5^ Department of Gastrointestinal Surgery, The First Affiliated Hospital of Guangzhou University of Chinese Medicine, Guangzhou 510405, Guangdong, China.; ^6^ Department of Hepatobiliary Diseases, The First Affiliated Hospital of Guangzhou University of Chinese Medicine, State Key Laboratory of Traditional Chinese Medicine Syndrome, Guangzhou 510405, Guangdong, China.; ^7^ Baiyun Hospital of The First Affiliated Hospital of Guangzhou University of Chinese Medicine, Guangzhou 510470, Guangdong, China.; ^8^ Center for Medical Research on Innovation and Translation, Guangzhou First People’s Hospital, Guangzhou 510180, Guangdong, China.; ^9^ Lingnan Institute of Spleen and Stomach Diseases, The First Affiliated Hospital of Guangzhou University of Chinese Medicine, Guangzhou 510405, Guangdong, China.

## Abstract

The development of an immunosuppressive microenvironment is a critical factor in stomach carcinogenesis. Polymorphonuclear myeloid-derived suppressor cells (PMN-MDSCs) serve a pivotal function in mediating immune suppression. However, the precise mechanisms underlying PMN-MDSCs infiltration into the tumor immune microenvironment (TIME) and their immunosuppressive functions remain poorly understood. In this investigation, we observed that PMN-MDSCs were up-regulated during stomach carcinogenesis, with gastric cancer (GC) cells secreting CCL26 to promote the infiltration of PMN-MDSCs into the TIME via the CX3CR1 receptor. The infiltrating CX3CR1^+^ PMN-MDSCs secreted transforming growth factor-β1 (TGF-β1), which, through the SMAD2/3/SNAI1 signaling pathway, further sustained the secretion of CCL26 by GC cells, establishing a positive feedback loop. Additionally, CX3CR1^+^ PMN-MDSCs suppressed mTOR signaling via TGF-β1 and competed with CD8^+^ T cells for glucose, disrupting glycolysis and leading to T cell exhaustion. Notably, inhibition of CX3CR1 reduced the infiltration of PMN-MDSCs, improved anti-PD-1 therapeutic efficacy, and suppressed tumor growth. In conclusions, this study illustrated that the CCL26–CX3CR1 axis mediates a positive feedback loop between GC cells and PMN-MDSCs, promoting CD8^+^ T cell exhaustion and tumorigenesis. Targeting CX3CR1 on PMN-MDSCs could serve as a potential therapeutic strategy to prevent stomach carcinogenesis.

## Introduction

Gastric cancer (GC) is the fifth most common malignancy and the third leading cause of cancer-related death worldwide [1]. Its intestinal type typically follows the Correa cascade, progressing from chronic non-atrophic gastritis (CNAG) to chronic atrophic gastritis (CAG), intestinal metaplasia (IM), low-grade intraepithelial neoplasia (LGIN), high-grade intraepithelial neoplasia (HGIN), and ultimately carcinoma [2]. Clinical outcomes are stage-dependent: 5-year survival approaches 95% in early GC but falls to 30% in advanced stage [3], underscoring the need to identify targets for earlier detection and interception.

Inflammation provides the context in which stomach carcinogenesis unfolds and reshapes the immune landscape [4,5]. Although acute responses recruit and activate effector cells to clear pathogens, persistent inflammation sustains cytokine production, drives the expansion and infiltration of immunosuppressive populations, and establishes an immunosuppressive microenvironment [6–9]. Nevertheless, the stage-specific immune alterations along this trajectory remain incompletely defined.

Myeloid-derived suppressor cells (MDSCs) are key players in immunosuppressive environment [10]. In pathological states such as cancer, disrupted cytokine signaling halts normal myeloid maturation, leading to accumulation of immature MDSCs [11]. They are typically classified into polymorphonuclear MDSCs (PMN-MDSCs) and monocytic MDSCs (M-MDSCs) [11]. Neutrophils are broadly defined as a lineage of polymorphonuclear myeloid cells derived from granulocyte–monocyte progenitors (GMPs) that span the developmental spectrum from early childhood to mature neutrophils [12]. Their physiological functions include anti-infective and anti-inflammatory activities [13]. PMN-MDSCs share a common origin as well as many morphological and phenotypic characteristics with neutrophils [14]. However, they typically arise through tumor-induced “emergency granulopoiesis” and possess potent immunosuppressive properties that distinguish them from conventional neutrophils [12]. PMN-MDSCs also differ from tumor-associated neutrophils (TANs), which are generally defined as neutrophils infiltrating the tumor immune microenvironment (TIME) [15,16]. Unlike TANs, PMN-MDSCs are not only confined to tumor tissues but also found in peripheral blood and the spleen, originating from bone marrow niches influenced remotely by tumor-derived factors [17]. Furthermore, TANs represent a heterogeneous population, including both “N1” (antitumorigenic) and “N2” (protumorigenic) phenotypes, whereas PMN-MDSCs consistently exhibit immunosuppressive and protumorigenic functions [17]. It has been proposed that PMN-MDSCs may represent a pre-tumoral subpopulation of TANs [16]. M-MDSCs, on the other hand, resemble monocytes and not only circulate systemically but also infiltrate tissues. In contrast, their more differentiated counterparts—macrophages and dendritic cells (DCs)—are primarily localized within tissues. In addition to these 2 subsets, another subtype, known as early MDSCs (e-MDSCs), has been recognized, which lack both macrophage and granulocyte markers [18]. In general, MDSCs expand in the bone marrow, enter the circulation, and accumulate in peripheral tissues, where they suppress antitumor immunity and facilitate tumor progression [19]. Recently, their role in shaping the TIME during stomach carcinogenesis has gained increasing recognition [20,21]. However, the specific chemokines and signaling pathways responsible for their recruitment remain poorly understood. Alongside MDSCs, T cell exhaustion is another barrier to effective antitumor immunity. Persistent inflammation and chronic antigen exposure drive the up-regulation of inhibitory receptors such as PD-1 on T cells, ultimately leading to a progressive loss of T cell function [22]. Understanding how T cell exhaustion emerges during stomach carcinogenesis is also vital for advancing immunotherapy.

Herein, we profiled the immune landscape across successive stages of stomach carcinogenesis and identified PMN-MDSCs as the most dynamically expanded immunosuppressive population. Mechanistically, malignant gastric epithelial cells secreted CCL26 to recruit PMN-MDSCs via the CX3CR1 receptor. In turn, CX3CR1^+^ PMN-MDSCs enhanced CCL26 expression through the transforming growth factor-β1 (TGF-β1)/SMAD2/3/SNAI1 pathway, forming a positive feedback loop. They also secreted TGF-β1 to inhibit mTOR signaling and competed with CD8^+^ T cells for glucose, impairing glycolysis and driving T cell exhaustion. Therapeutically, CX3CR1 blockade reduced PMN-MDSCs infiltration, improved anti-PD-1 efficacy, and suppressed stomach carcinogenesis. These findings underscore PMN-MDSCs and CX3CR1 as potential targets for immunoprevention and early intervention in GC.

## Results

### PMN-MDSCs exhibited prominent immune dysregulation during stomach carcinogenesis

**Fig. 1.**
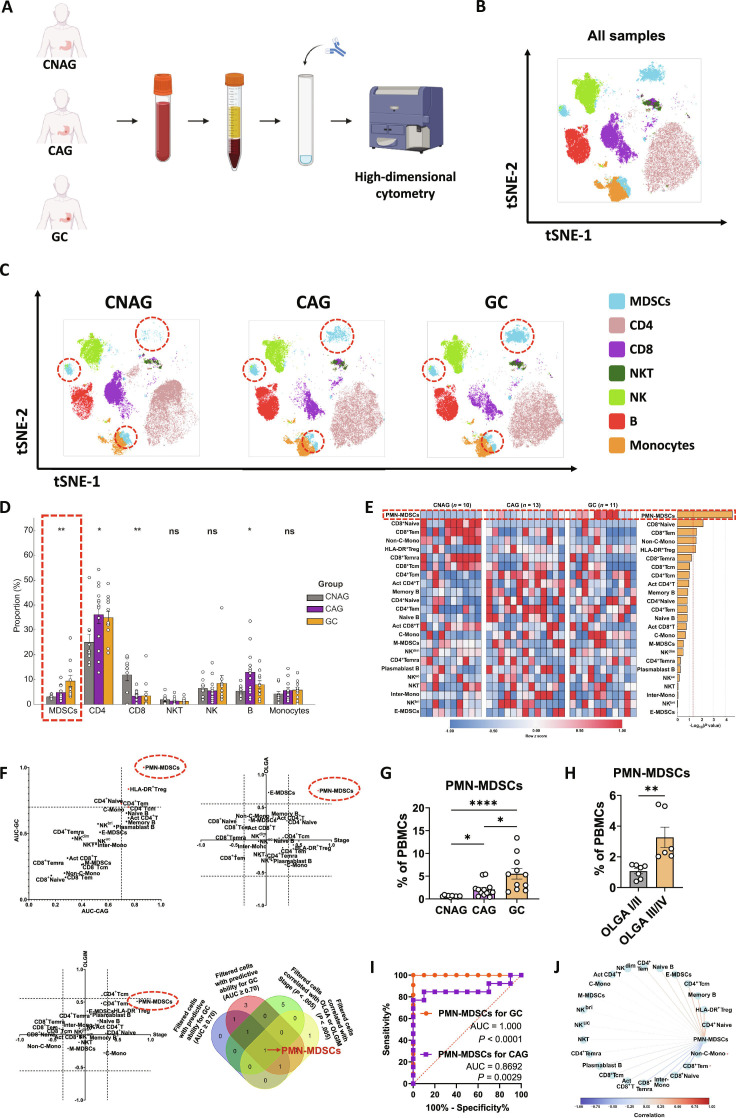
PMN-MDSCs exhibited prominent immune dysregulation during stomach carcinogenesis. (A) Schematic of the experimental design. Blood samples were collected from patients with CNAG, CAG, and GC. High-dimensional flow cytometry was used to analyze immune cell populations. (B) t-SNE plot of all samples showing the distribution of various immune cell subtypes across all groups. (C) t-SNE plots separated by disease group illustrating the immune cell landscape. (D) Proportions of different immune cell subsets across CNAG, CAG, and GC groups. (E) Heatmap illustrating the patterns of various immune cells across different groups. (F) The selection of PMN-MDSCs as immune cells correlated with GC. The top left panel displays a scatterplot, where the *x* axis represents AUC-CAG and the *y* axis represents AUC-GC. The top right panel shows the correlation between different immune cell populations and cancer stages (CNAG→CAG→GC) along with OLGA staging. The bottom left panel depicts the relationship between immune cells and cancer stages (CNAG→CAG→GC) along with OLGIM staging. The bottom right panel features a Venn diagram, demonstrating the filtering process that led to the selection of PMN-MDSCs based on their predictive ability for GC and their significant correlation with stage, OLGA, and OLGIM. (G) Quantification of PMN-MDSCs as a percentage of lymphocytes in CNAG, CAG, and GC groups. (H) Quantification of PMN-MDSCs as a percentage of lymphocytes between OLGA III/IV and OLGA I/II stages. (I) ROC curves for PMN-MDSCs in distinguishing between GC and CAG, with corresponding AUC values. (J) Correlation analysis of PMN-MDSCs and other immune cells. Data are presented as mean ± SEM for the bar chart. The sample sizes are as follows: CNAG (*n* = 10), CAG (*n* = 13), and GC (*n* = 11), with OLGA I/II (*n* = 7) and OLGA III/IV (*n* = 6). Data of (D) were analyzed using one-way ANOVA. Data of (F), (I), and (J) were analyzed by AUC and Spearman or Pearson correlation analysis. Data of (G) were compared using the Kruskal–Wallis test followed by Dunnett’s post hoc test. Data of (H) were compared using the Mann–Whitney test. *P < 0.05, **P < 0.01, ***P < 0.005, ****P < 0.0001, and ns for nonsignificant.

To elucidate the immune landscape across different stages of stomach carcinogenesis, we performed high-dimensional flow cytometry analysis on blood samples from patients with CNAG (*n* = 10), CAG (*n* = 13), and GC (*n* = 11) (Fig. 1A). The t-distributed stochastic neighbor embedding (t-SNE) analysis revealed distinct clusters of immune cell populations, including MDSCs, CD4^+^ T cells, CD8^+^ T cells, natural killer T (NKT) cells, NK cells, B cells, and monocytes (Fig. 1B), with the gating strategy described (Fig. S1). Further stratification of t-SNE plots by disease state demonstrated dynamic changes in the immune cell landscape throughout disease progression (Fig. 1C). As mentioned earlier, MDSCs exhibit compositional heterogeneity, primarily consisting of monocytic and polymorphonuclear subtypes, with a minor proportion of early type. Consequently, 3 distinct clusters of MDSCs were observed in the t-SNE analysis. MDSCs exhibited a significant increase during stomach carcinogenesis, accompanied by a notable elevation of CD4^+^ T cells and a marked down-regulation of CD8^+^ T cells (Fig. 1D). The subtypes of MDSCs, CD4^+^ T cells, CD8^+^ T cells, NK cells, B cells, and monocytes were further delineated (Figs. S2 to S4). Among all subtypes, PMN-MDSCs showed the most significant alteration, exhibiting a pronounced increase during stomach carcinogenesis (Fig. 1E). Furthermore, human leukocyte antigen (HLA)-DR^+^ regulatory T (Treg) cells were significantly up-regulated (Fig. 1E and Fig. S3D), while naive CD8^+^ T cells, effector memory CD8^+^ T cells (CD8^+^ Tem) (Fig. 1E and Fig. S3H), and non-classical monocytes (non-C-mono) (Fig. 1E and Fig. S2I) showed significant down-regulation.

To identify critical cell types associated with stomach carcinogenesis, receiver operating characteristic (ROC) analysis and correlation tests were performed on each immune cell. Subtypes with an area under the curve (AUC) ≥ 0.7 for diagnosing CAG and GC, and significantly correlated with stage and cancer risk indicators [operative link for gastritis assessment (OLGA) or operative link for gastric intestinal metaplasia assessment (OLGIM)], were considered as key immune cells. PMN-MDSCs were the only subset that met all the criteria (Fig. 1F). Their proportion significantly increased from CNAG to CAG and GC (Fig. 1G). PMN-MDSCs were also significantly elevated in CAG patients with higher OLGA stages (Fig. 1H). ROC curve analysis showed that PMN-MDSCs had an excellent predictive ability for diagnosing GC and a strong predictive capability for diagnosing CAG (Fig. 1I). The correlation plot (Fig. 1J) illustrated that PMN-MDSCs had a positive correlation with CD4^+^ T subtypes but a negative correlation with CD8^+^ T subtypes. These results suggested that PMN-MDSCs significantly increased during stomach carcinogenesis, correlated with disease progression, and served as key biomarkers for diagnosing and monitoring GC. In addition, they might exert a negative effect on CD8^+^ T cells.

### Increased infiltration of PMN-MDSCs in stomach mucosa during carcinogenesis

**Fig. 2.**
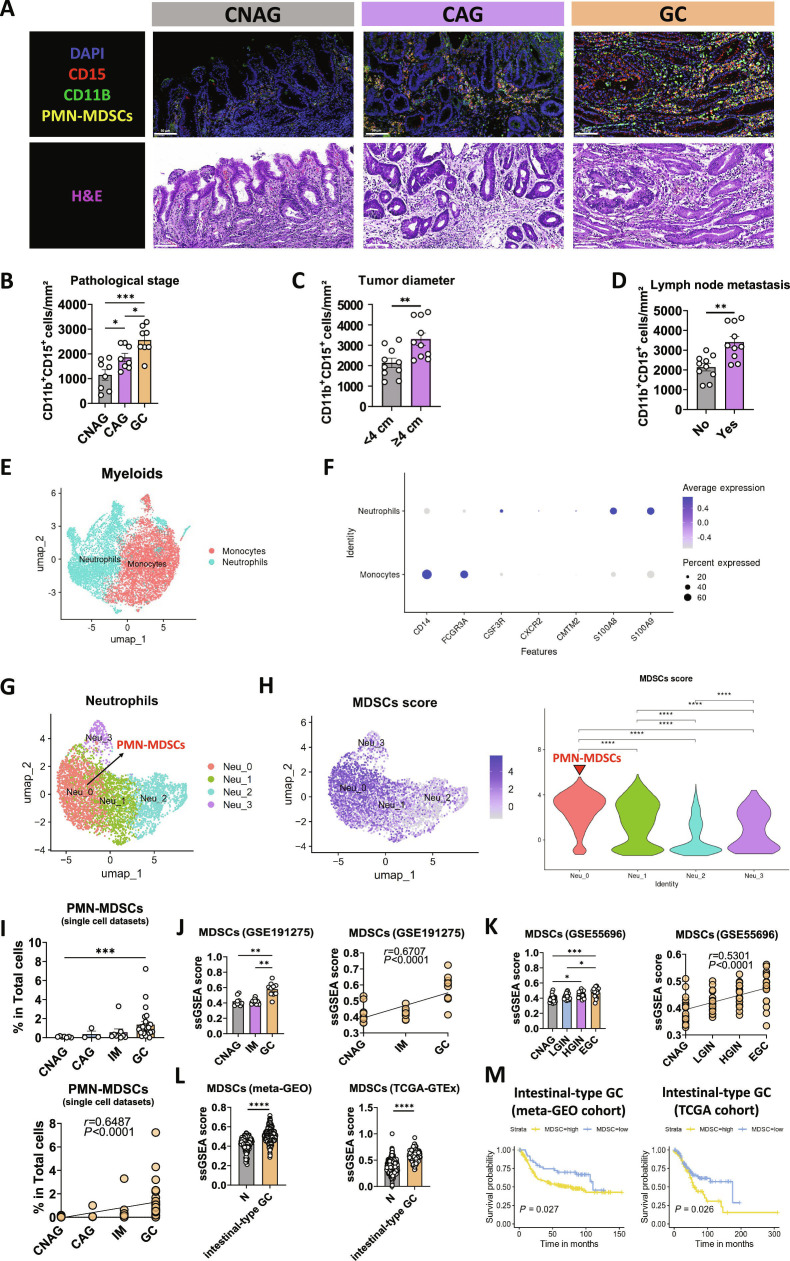
Increased infiltration of PMN-MDSCs in stomach mucosa during carcinogenesis. (A) Immunofluorescence and H&E staining of stomach mucosa sections from patients with CNAG, CAG, and GC. The upper panels show immunofluorescence images stained for CD15 (red) and CD11b (green), highlighting PMN-MDSCs infiltration. The lower panels show corresponding H&E staining. (B to D) Quantification of CD11b^+^ CD15^+^ PMN-MDSCs per mm^2^ in stomach mucosa across different pathological stages (CNAG, *n =* 8; CAG, *n =* 8; GC, *n =* 8) (B), tumor diameters (≥4 cm, *n =* 10; ≤4 cm, *n =* 10) (C), and in the presence or absence of lymph node metastasis (no, *n =* 10; yes, *n =* 10) (D). (E) UMAP plots illustrating neutrophils and monocytes in myeloids. Data were derived from 4 scRNA-seq datasets (GEO: GSE134520, GSE183904, GSE150290; dbGaP: phs001818.v2), totaling *n =* 44 patients. Processing was performed in Seurat (v4.3.0) with quality control (200 to 6,000 genes per cell; mitochondrial RNA ≤ 10%), LogNormalize normalization, and selection of 3,000 highly variable genes, followed by PCA (first 20 components). Datasets were integrated with Harmony for batch correction. Analyses were restricted to the myeloid compartment; clustering (resolution *r* = 0.1) and UMAP were performed on the Harmony-corrected principal components using dimensions 1 to 20. (F) Dot plot of neutrophils and monocyte markers in myeloids. Same datasets and preprocessing pipeline as in (E). Dot size indicates the percentage of cells expressing each gene, and color indicates average scaled expression. (G) UMAP plot showing the 4 subsets of neutrophils. Same datasets and preprocessing pipeline as in (E). Analyses were restricted to the neutrophil compartment. Clustering (resolution *r* = 0.1) and UMAP were performed on the Harmony-corrected principal components using dimensions 1 to 20. (H) UMAP plot and violin plot showing the MDSCs score of the 4 subsets of neutrophils. Scores were computed in Seurat using AddModuleScore with the MDSCs gene set (IL1B, CCR1, CXCL2, GRINA, IER3). (I) Quantification of relative PMN-MDSCs percentages in scRNA-seq datasets across CNAG (*n =* 7), CAG (*n =* 3), IM (*n =* 9), and GC (*n =* 25) groups. The correlation between PMN-MDSCs percentage and pathological stage is shown on the bottom. (J) ssGSEA scores for MDSCs in stomach mucosa across CNAG (*n =* 10), IM (*n =* 10), and GC (*n =* 10) samples from the GSE191275 dataset. The right panel shows the correlation of ssGSEA scores with disease progression. (K) ssGSEA scores for MDSCs in CNAG (*n =* 19), LGIN (*n =* 19), HGIN (*n =* 20), and EGC (*n =* 19) samples from the GSE55696 dataset. The right panel shows the correlation of ssGSEA scores with disease progression. (L) Comparison of ssGSEA scores for MDSCs between normal tissue and intestinal-type GC tissue in both meta-GEO (*n =* 133 versus *n =* 295) and TCGA-GTEx (*n =* 359 versus *n =* 165) cohorts. (M) Kaplan–Meier survival analysis showing overall survival of patients with intestinal-type GC stratified by MDSCs-high and MDSCs-low groups in both meta-GEO (*n =* 147 versus *n =* 148) and TCGA (*n =* 82 versus *n =* 83) cohorts. Data are presented as mean ± SEM for the bar charts. Data of (B) were analyzed using one-way ANOVA followed by Bonferroni’s post hoc test. Data of (C) and (D) were analyzed using Student’s *t* test. Bar plots of (I) to (L) were compared using the Kruskal–Wallis test followed by Dunnett’s post hoc test. Correlation analysis of (I) to (K) were performed using Spearman’s correlation. Data of (M) were analyzed using the log-rank test. *P < 0.05, **P < 0.01, ***P < 0.005, ****P < 0.0001, and ns for nonsignificant.

Although CD66b is also used to identify PMN-MDSCs, it exists in a soluble form that can be released and bind to CEACAM1 [23,24], introducing background noise and complicating assay interpretation. In contrast, CD15 does not have an analogous soluble form issue. To avoid the background noise caused by soluble CD66b and following the approach of Lin and colleagues [25], we utilized CD11B^+^CD15^+^ rather than CD11B^+^CD66b^+^ to identify PMN-MDSCs in immunofluorescence (Fig. 2A). Quantitative analysis revealed a progressive and significant increase in CD15^+^ CD11B^+^ cells/mm^2^ from CNAG to CAG and GC tissues (Fig. 2B). In GC, patients with larger tumors (≥4 cm) or lymph node metastasis exhibited substantially elevated PMN-MDSCs frequencies compared to those with smaller tumors (≤4 cm) or without metastasis (Fig. 2C and D).

Bioinformatics analysis further corroborated these findings. Single-cell RNA sequencing (scRNA-seq) data provided insights into the cellular composition of the gastric mucosa during carcinogenesis (Fig. S5A and B). Following methods from previous study [26], we identified PMN-MDSCs and M-MDSCs within myeloid cells. First, by dimensionality reduction, we identified neutrophils and monocytes within the myeloid cells (Fig. 2E and F). Next, we performed dimensionality reduction on the neutrophils and monocytes, identifying 4 distinct clusters (neutrophils: Neu_0, Neu_1 Neu_2 Neu_3, monocytes: Mono_0, Mono_1, Mono_2, Mono_3). Functional enrichment separated neutrophil subclusters into immunosuppression (Neu_0), antigen presentation (Neu_1), inflammation (Neu_2), and angiogenesis (Neu_3) (Fig. S6A), consistent with the functional classification of tumor-infiltrating neutrophils reported by Gao and colleagues [27]. Neu_0 showed the highest MDSCs and immunosuppressive scores, high OLR1 (LOX-1), and enriched CX3CR1, linking this subset mechanistically to our axis (Fig. S6B). Mono_2 (Fig. S6C and D) were found to have the highest MDSCs scores (Fig. S6E), along with high immunosuppressive scores (Fig. S6F). Taken together, Neu_0 were classified as PMN-MDSCs (Fig. 2G and H) and Mono_0 as M-MDSCs, respectively. During stomach carcinogenesis, the proportion of PMN-MDSCs (Fig. 2I) and M-MDSCs (Fig. S6H) gradually increases and shows a positive correlation with disease progression, with PMN-MDSCs exhibiting a more pronounced increase.

In bulk RNA-seq data, the single-sample gene set enrichment analysis (ssGSEA) score for MDSCs was analyzed to assess their association with stomach carcinogenesis and GC prognosis. In the GSE191275 dataset, MDSCs scores were significantly elevated in GC tissues compared to CAG and IM tissues, exhibiting a positive correlation with disease progression (Fig. 2J). Similarly, the GSE55696 dataset showed increased MDSCs scores from CNAG to early gastric cancer (EGC), with a positive correlation (Fig. 2K). Meta-GEO and TCGA-GTEx cohorts confirmed higher MDSCs scores in intestinal-type GC versus normal tissues (Fig. 2L). Kaplan–Meier analysis indicated that elevated MDSCs scores were correlated with reduced overall survival in both cohorts (Fig. 2M). These findings underscore the protumorigenic role of PMN-MDSCs within the microenvironment of stomach carcinogenesis.

### CCL26 induced PMN-MDSCs infiltration through CX3CR1 during stomach carcinogenesis

**Fig. 3.**
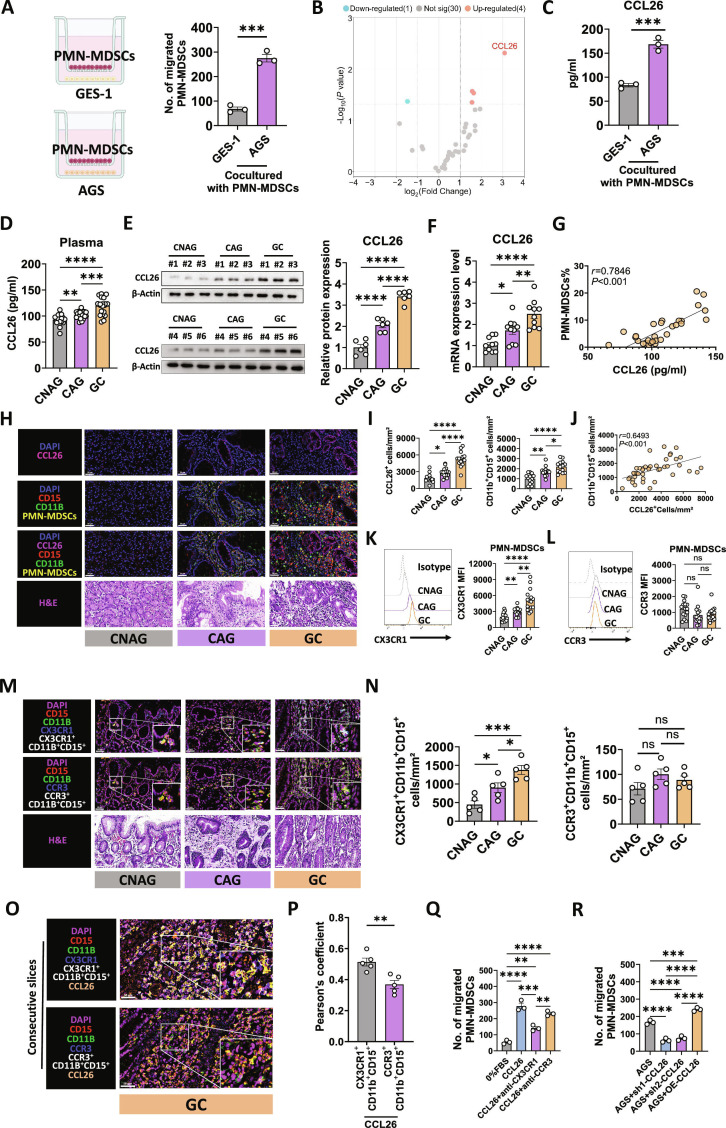
CCL26 induced PMN-MDSCs infiltration via CX3CR1 during stomach carcinogenesis. (A) Transwell coculture assay showing the migration of PMN-MDSCs to GES-1 and AGS cells. The right graph quantifies the number of migrated PMN-MDSCs. *n =* 3, biological replicates. (B) Volcano plot showing the differential expression of chemokines in AGS cells cocultured with PMN-MDSCs compared with GES-1 cells cocultured with PMN-MDSCs. *n =* 3, biological replicates. (C) CCL26 levels in coculture medium from GES-1 or AGS cells with PMN-MDSCs. *n =* 3, biological replicates. (D) Plasma CCL26 levels in patients with CNAG (*n =* 18), CAG (*n =* 23), and GC (*n =* 21) were quantified using ELISA. (E) Western blot analysis of CCL26 protein expression levels between CNAG, CAG, and GC groups. *n =* 6 per group. (F) RT-qPCR analysis was conducted to compare mRNA expression levels of CCL26 across CNAG, CAG, and GC groups. *n =* 10 per group. (G) Correlation between plasma CCL26 levels and the percentage of PMN-MDSCs. CNAG (*n =* 10), CAG (*n =* 10), and GC (*n =* 10). (H) Immunofluorescence staining of stomach tissues from CNAG, CAG, and GC groups for CCL26 (pink), CD15 (red), and CD11b (green). Corresponding H&E staining images are shown in the bottom panel. (I) Quantification of CCL26^+^ and CD11b^+^ CD15^+^ cells per mm^2^ in stomach mucosa from CNAG (*n =* 13), CAG (*n =* 16), and GC (*n =* 14) groups. (J) Correlation between CCL26^+^ cells and CD11b^+^ CD15^+^ cells in stomach mucosa. CNAG (*n =* 13), CAG (*n =* 16), and GC (*n =* 14). (K) Flow cytometry analysis of CX3CR1 expression in PMN-MDSCs from patients with CNAG, CAG, and GC. Bar graph on the right quantifies the MFI of CX3CR1 across the 3 groups. *n =* 15 per group. (L) Flow cytometry analysis of CCR3 expression in PMN-MDSCs from patients with CNAG, CAG, and GC. Bar graph on the right quantifies the MFI of CCR3 across the 3 groups. *n =* 15 per group. (M) Immunofluorescence analysis of CD15, CD11b, PMN-MDSCs, and CCR3 expression in gastric tissues from CNAG, CAG, and GC groups. (N) Boxplot representation of CX3CR1^+^ CD11b^+^ CD15^+^ and CCR3^+^ CD11b^+^ CD15^+^ cell populations across CNAG, CAG, and GC groups. *n =* 5 per group. (O) Quadruple immunofluorescence staining of gastric mucosa. Insets provide a magnified view of the regions highlighted by white boxes. (P) Pearson’s correlation analysis measuring the colocalization of CCL26 with CX3CR1^+^ CD11b^+^ CD15^+^ and CCR3^+^ CD11b^+^ CD15^+^ cell populations. *n =* 5 per group. (Q) Number of migrated PMN-MDSCs in response to conditioned media of 0% FBS, CCL26, CCL26 with anti-CX3CR1 antibodies, and CCL26 with anti-CCR3 antibodies. *n =* 3, biological replicates. (R) Number of migrated PMN-MDSCs in response to conditioned media of AGS cells, AGS cells transfected with sh26-1, AGS cells transfected with sh26-2, and AGS cells transfected with CCL26 overexpression plasmids. *n =* 3, biological replicates. Data are presented as mean ± SEM for the bar charts. Data of (A) and (C) were analyzed using Student’s *t* test. Data of (D) to (F), (I), (K), (L), (N), (Q), and (R) were analyzed using one-way ANOVA followed by Bonferroni’s post hoc test. Correlation analysis of (G) was performed using Pearson correlation. Correlation analysis of (J) was performed using Spearman’s correlation. *P < 0.05, **P < 0.01, ***P < 0.005, ****P < 0.0001, and ns for nonsignificant.

Chemokines are signaling proteins that serve a pivotal function in modulating the migration and recruitment of immune cells, including MDSCs [28]. Identifying key chemokines involved in MDSCs trafficking is essential for understanding their function within the TIME and uncovering potential therapeutic targets. To investigate the chemokines responsible for PMN-MDSC infiltration, migration assays were performed using GES-1 (a normal human gastric epithelial cell line) and AGS (a human GC cell line) in combination with PMN-MDSCs isolated from GC patients. We found that AGS possesses higher chemotactic ability for PMN-MDSCs compared with GES-1 (Fig. 3A). Luminex assay indicated that CCL26 exhibited the most significant up-regulated alteration in the AGS cocultured medium (Fig. 3B), which was confirmed by enzyme-linked immunosorbent assay (ELISA) (Fig. 3C). Bioinformatics analysis further supported our findings. We screened novel chemokines associated with PMN-MDSCs infiltration and disease progression in bulk sequence datasets (GSE55696, GSE191275, meta-GEO, and TCGA) based on the following criteria: (a) positively correlated with MDSCs, (b) up-regulated in GC and CAG, and (c) positively correlated with poor prognosis. The intersection of the above criteria identified CCL26 as the only chemokine of interest (Fig. S7A to D). In the scRNA-seq dataset of stomach carcinogenesis, CCL26 exhibited a significant up-regulation trend in cancer cells, with its expression levels positively correlating with disease progression (Fig. S7E). Besides, the mRNA expression level of CCL26 in cancer cells was positively linked to the proportions of PMN-MDSCs, while no such correlation was observed with M-MDSCs (Fig. S7F). The quantitative polymerase chain reaction (qPCR) and Western blotting (WB) results demonstrated an elevation of CCL26 mRNA (Fig. 3D) and protein levels (Fig. 3E) in the gastric mucosa during stomach carcinogenesis. Similarly, CCL26 plasma levels also demonstrated a marked increase (Fig. 3F), significantly correlated with the percentage of PMN-MDSCs (Fig. 3G).

Immunofluorescence staining of paraffin-embedded tissue sections revealed that CCL26 expression was weak in CNAG but increased markedly with advancing pathological stages (Fig. 3H and I). PMN-MDSCs also showed increased infiltration from CNAG to GC (Fig. 3H and I). In terms of localization, CCL26 was primarily expressed in stomach glands (epithelial cells), aligning with the up-regulation of CCL26 observed in cancer cells in our previous scRNA-seq analysis, whereas PMN-MDSCs preferentially infiltrated stromal areas. A strong positive correlation was observed between the number of CCL26^+^ cells and PMN-MDSCs (Fig. 3J). Collectively, these findings suggest that CCL26 may recruit PMN-MDSCs during stomach carcinogenesis.

CCL26 binds to CCR3 or CX3CR1 to exert chemotactic effects [29,30]. To clarify which receptor mediates CCL26-induced infiltration of PMN-MDSCs, we examined CX3CR1 and CCR3 expression in PMN-MDSCs derived from the peripheral blood of patients at different stages of stomach carcinogenesis. Flow cytometry revealed a progressive increase in CX3CR1 mean fluorescence intensity (MFI) from CNAG to GC, with significant differences across stages (Fig. 3K). In contrast, CCR3 expression remained unchanged (Fig. 3L). Matched transcriptomic and data-independent acquisition (DIA) proteomic datasets consistently show progressive up-regulation of CX3CR1 in patient PMN-MDSCs across the CNAG, CAG, and GC stages (Fig. S8). scRNA-seq Uniform Manifold Approximation and Projection (UMAP) localize CX3CR1 predominantly to the Neu_0 (PMN-MDSCs) cluster among neutrophil subtypes and to PMN-MDSCs within all major cell populations (Fig. S6B and I). These results reconcile prior reports by demonstrating a disease stage-dependent, PMN-MDSCs-biased CX3CR1 increase. By contrast, neither CX3CR1 nor CCR3 expression varied significantly in M-MDSCs across stages (Fig. S7I to L). To evaluate receptor-specific infiltration in tissue, we performed immunofluorescence staining for CX3CR1^+^ CD11B^+^ CD15^+^ and CCR3^+^ CD11B^+^ CD15^+^ cells across CNAG, CAG, and GC stages (Fig. 3M). Quantitative analysis revealed a significant increase in CX3CR1^+^ CD11B^+^ CD15^+^ cells in CAG and GC compared with CNAG (Fig. 3N), whereas CCR3^+^ CD11B^+^ CD15^+^ cells showed no difference. To explore spatial relationships, consecutive GC tissue sections were stained for CCL26 alongside these cell subsets (Fig. 3O). Pearson’s correlation analysis demonstrated significant colocalization of CCL26 with CX3CR1^+^ CD11B^+^ CD15^+^ cells, but weaker overlap with CCR3^+^ CD11B^+^ CD15^+^ cells (Fig. 3P). Collectively, these results suggest that CCL26 drives PMN-MDSCs migration primarily through interaction with CX3CR1, rather than CCR3.

To further investigate CCL26-mediated PMN-MDSCs migration, PMN-MDSCs isolated from patients with GC were exposed to conditioned media. The number of migrated PMN-MDSCs showed a significant increase when exposed to CCL26 relative to the control (Fig. 3Q). Blocking CX3CR1 significantly reduced CCL26-induced migration. Conversely, blocking CCR3 had no significant effect, indicating that CCL26-mediated migration was dependent on CX3CR1 (Fig. 3Q). Additionally, migration assays were performed using conditioned media from AGS cells, AGS cells with CCL26 knockdown (sh-CCL26), and AGS cells overexpressing CCL26 (OE-CCL26). The results showed significantly higher PMN-MDSCs migration in the OE-CCL26 group and significantly lower migration in the sh-CCL26 group (Fig. 3R), confirming the role of CCL26 in promoting PMN-MDSCs migration. Furthermore, PMN-MDSCs from the spleens of tumor-bearing wild-type (WT) and *Cx3cr1^−/−^* mice were also subjected to migration assays. The number of migrated PMN-MDSCs was markedly elevated in WT mice versus *Cx3cr1^−/−^* mice when exposed to CCL26 (Fig. S9A), strongly indicating that CX3CR1 is crucial for CCL26-mediated PMN-MDSCs migration.

### CX3CR1^+^ PMN-MDSCs secreted TGF-β1 to promote CCL26 expression in GC cells

**Fig. 4.**
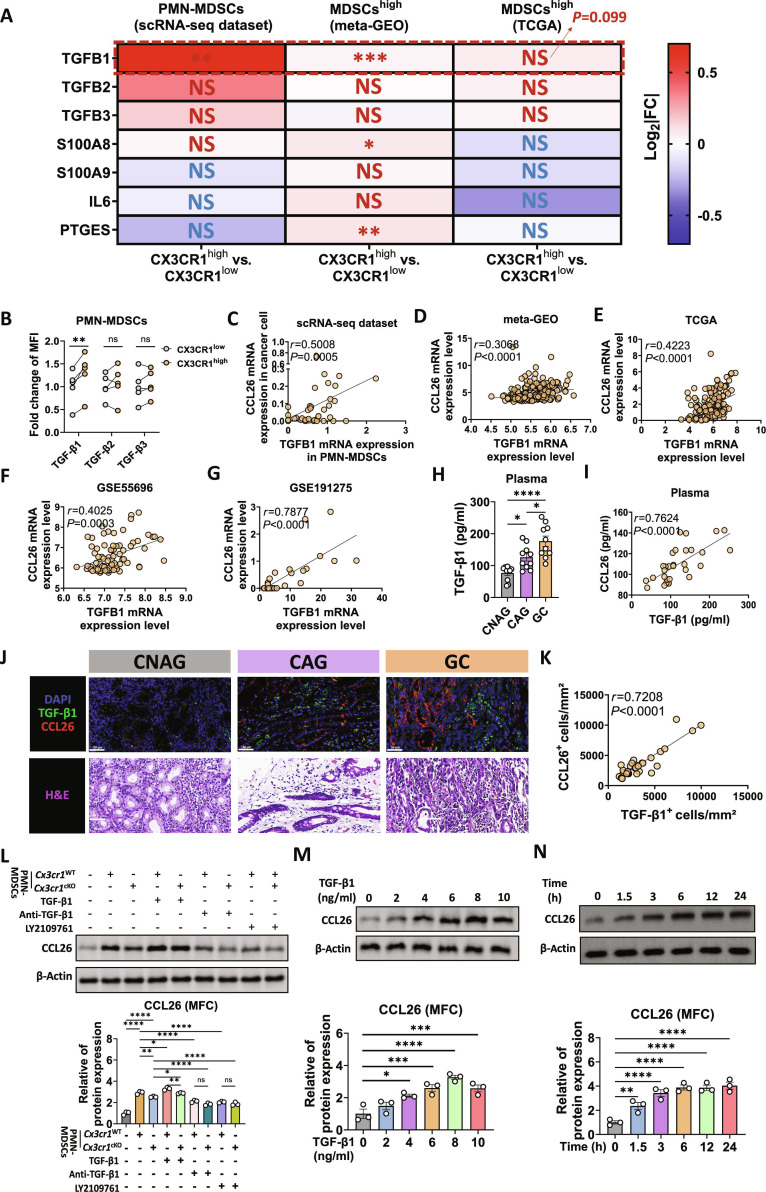
PMN-MDSCs secreted TGF-β1 to promote CCL26 expression in GC cells. (A) Heatmap showing the log_2_ fold change (Log_2_|FC|) and *P* value of protumorigenic and immunosuppressive factors, including TGFB1, TGFB2, TGFB3, S100A8, S100A9, IL6, and PTGES. Gene expression analysis was performed to compare CX3CR1^high^ (*n =* 12) and CX3CR1^low^ MDSCs (*n =* 13) in scRNA-seq datasets, as well as CX3CR1^high^ and CX3CR1^low^ subgroups within MDSCs^high^ populations across meta-GEO (*n =* 147 versus *n =* 148) and TCGA (*n =* 82 versus *n =* 83) datasets. (B) Fold change in the MFI of TGF-β1, TGF-β2, and TGF-β3 expression between CX3CR1^low^ PMN-MDSCs and CX3CR1^high^ PMN-MDSCs in the peripheral blood of GC patients. *n =* 5 per group. (C) Correlation between CCL26 mRNA expression in cancer cells and TGFB1 mRNA expression in PMN-MDSCs, as analyzed in the scRNA-seq dataset of stomach carcinogenesis. CNAG (*n =* 7), CAG (*n =* 3), IM (*n =* 9), and GC (*n =* 25). (D) Correlation between CCL26 and TGFB1 mRNA expression levels in meta-GEO dataset (*n =* 295). (E) Correlation between CCL26 and TGFB1 mRNA expression levels in TCGA dataset (*n =* 165). (F) Correlation between CCL26 and TGFB1 mRNA expression levels in GSE55696 dataset. CNAG (*n =* 19), LGIN (*n =* 19), HGIN (*n =* 20), and EGC (*n =* 19). (G) Correlation between CCL26 and TGFB1 mRNA expression levels in GSE191275 dataset. CNAG (*n =* 10), IM (*n =* 10), and GC (*n =* 10). (H) TGF-β1 levels in plasma from patients with CNAG (*n =* 10), CAG (*n =* 10), and GC (*n =* 10). (I) Correlation between plasma levels of TGF-β1 and CCL26. CNAG (*n =* 10), CAG (*n =* 10), and GC (*n =* 10). (J) Representative immunofluorescence staining images of stomach mucosa from CNAG, CAG, and GC groups for TGF-β1 (green) and CCL26 (red). Corresponding H&E staining images are shown below. (K) Correlation between TGF-β1^+^ cells and CCL26^+^ cells in stomach mucosa. CNAG (*n =* 10), CAG (*n =* 11), and GC (*n =* 11). (L) Western blot analysis of CCL26 expression in MFC cells cocultured with *Cx3cr1*^cKO^ and *Cx3cr1*^WT^ PMN-MDSCs and treated with either anti-TGF-β1 antibody or TGFβRI/II inhibitor (LY210976). *n =* 3, biological replicates. (M and N) Western blot analysis showing the dose-dependent (M) and time-dependent (N) effects of TGF-β1 on CCL26 expression in MFC cells. *n =* 3, biological replicates. Data are presented as mean ± SEM for the bar charts. Data of (A) were analyzed using the Student’s *t* test. Data of (B) were analyzed using paired *t* test. Correlation analyses of (C) to (G), (I), and (K) were performed using Spearman’s correlation. Data of (H) and (L) to (N) were analyzed using one-way ANOVA followed by Bonferroni’s post hoc test. *P < 0.05, **P < 0.01, ***P < 0.005, ****P < 0.0001, and ns for nonsignificant.

To examine the function of CX3CR1^+^ PMN-MDSCs in the TIME, we compared factors in MDSCs^high^ samples between CX3CR1^high^ and CX3CR1^low^ groups in public datasets (Fig. 4A). The factors of primary interest were those secreted by MDSCs, which have been previously reported to be both protumorigenic and immunosuppressive, including TGFB1, TGFB2, TGFB3 [31–33], S100A8, S100A9 [34,35], IL6 [36–38], and PTGES [39–41]. In the scRNA-seq datasets, the up-regulation of TGFB1 in CX3CR1^high^ MDSCs was confirmed, while no significant changes were observed for the other factors. In the meta-GEO cohort, TGFB1, S100A8, and PTGES were significantly up-regulated in CX3CR1^high^ samples, while TGFB2, TGFB3, S100A9, and IL6 did not show significant changes. In the TCGA cohort, no significant differences were observed for any of the factors, while TGFB1 showed a near-statistical significance increase in CX3CR1^high^ samples. Collectively, the consistent up-regulation of TGFB1 across the datasets suggested its potential role in CX3CR1^high^ PMN-MDSCs. In addition, PMN-MDSCs from the spleens of tumor-bearing WT and *Cx3cr1^−/−^* mice were isolated and subjected to RNA-seq analysis. Among above factors, *Tgfb1* was significantly down-regulated in PMN-MDSCs from *Cx3cr1^−/−^* mice (Fig. S9B). The qPCR results further validated this significant down-regulation of *Tgfb1* mRNA expression in PMN-MDSCs from *Cx3cr1^−/−^* mice (Fig. S9C), while no significant changes were observed in *Tgfb2* and *Tgfb3*. Additionally, in the peripheral blood of GC patients, TGF-β1 expression was much higher on CX3CR1^high^ PMN-MDSCs than on CX3CR1^low^ PMN-MDSCs (Fig. 4B), while no significant differences were observed for TGF-β2 and TGF-β3. These findings suggested that TGF-β1 was the key factor through which CCL26-recruited PMN-MDSCs interfere with the GC TIME.

Much crosstalk occurs between MDSCs and tumor cells [42]. Bioinformatics analysis across multiple datasets indicated a positive link between the expression levels of TGFB1 and CCL26 (Fig. 4C to G). The concentration of TGF-β1 in plasma significantly increased as the disease progressed (Fig. 4H) and showed a positive correlation with CCL26 (Fig. 4I). Immunofluorescence staining of tissue sections from CNAG, CAG, and GC samples revealed that the coexpression of TGF-β1 (green) and CCL26 (red) increased progressively from CNAG to GC (Fig. 4J and K). Regarding localization, CCL26 was primarily expressed in stomach glands, while TGF-β1 was primarily expressed in the stromal areas. Then, we generated *Cx3cr1^fl/fl^S100a8^cre^* (*Cx3cr1*^cKO^) mice and isolated *Cx3cr1*^cKO^ versus *Cx3cr1*^WT^ PMN-MDSCs for ex vivo assays. CX3CR1 sufficiency enhanced tumor cell CCL26 induction; this effect was attenuated by TGF-β1 blockade and reduced in *Cx3cr1*^cKO^ PMN-MDSCs (Fig. 4L). Direct stimulation of mouse forestomach carcinoma cell line (MFC) cells with TGF-β1 revealed a dose- and time-dependent increase in CCL26 expression (Fig. 4M and N). Collectively, these findings demonstrated that CX3CR1^+^ PMN-MDSCs secrete TGF-β1, which in turn promotes CCL26 expression in GC cells.

### TGF-β1 promoted CCL26 expression by activating the SMAD2/3/SNAI1 pathway in GC cells

To explore the mechanism by which TGF-β1 promotes CCL26 expression, we identified shared genes that were positively correlated with TGFB1, CCL26, pathological stage, and poor prognosis, resulting in an intersection of 24 genes (Fig. S10A). Enrichment analysis revealed significant pathways related to the “transcriptional activity of SMAD2/SMAD3/SMAD4 heterotrimer”, “TGF-beta receptor signaling activates SMADs”, and “SMAD2/SMAD3/SMAD4 heterotrimer regulates transcription” (Fig. S10B). These findings suggest that downstream transcription factors (TFs) of SMADs may be activated. It has been reported that the expression of TFs such as SNAI1, SLUG, ZEB1, and ZEB2 is promoted when TGF-β1 binds to the TGF-β receptor and activates the SMAD signaling pathway [43]. Therefore, we utilized similar strategy to screen key TFs involved and finally SNAI1 was identified (Fig. S10C). Accordingly, we hypothesized that TGF-β1 promotes CCL26 expression in GC cells by activating the SMAD2/3/SNAI1 pathway.

**Fig. 5.**
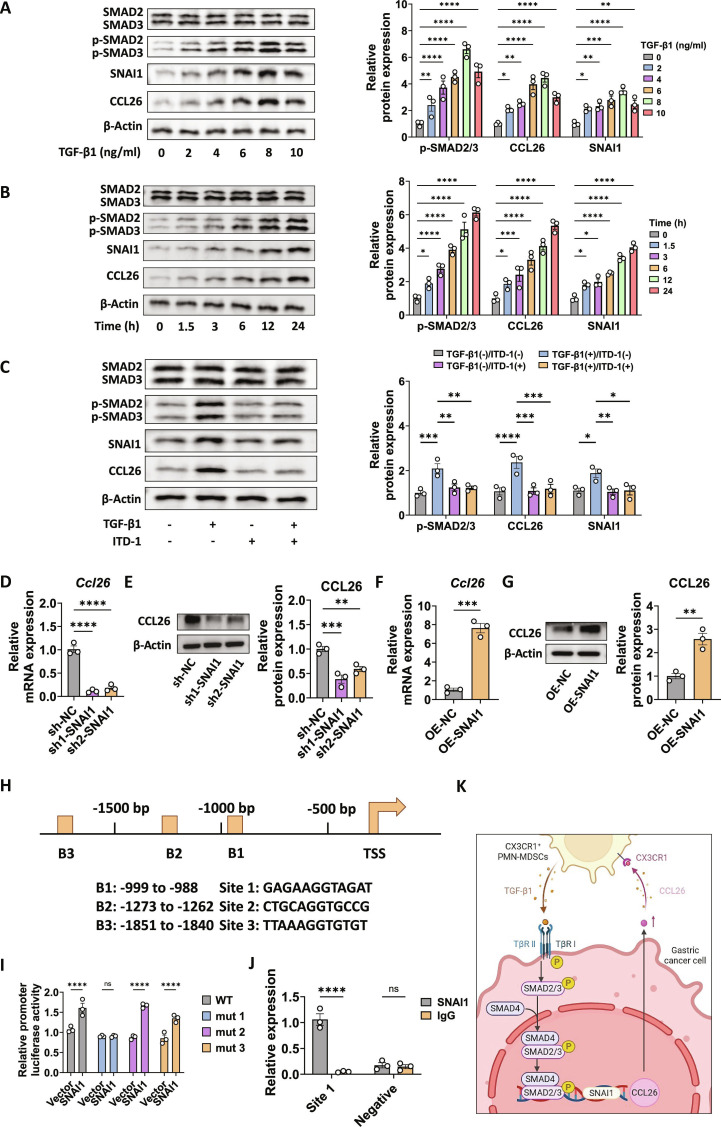
TGF-β1 promoted CCL26 expression by activating the SMAD2/3/SNAI1 pathway in GC cells. (A) Western blot analysis of SMAD2, SMAD3, p-SMAD2, p-SMAD3, SNAI1, and CCL26 in MFC cells treated with increasing concentrations of TGF-β1 (0, 2, 4, 6, 8, and 10 ng/ml). (B) Time-course Western blot analysis showing the levels of SMAD2, SMAD3, p-SMAD2, p-SMAD3, SNAI1, and CCL26 in MFC cells treated with TGF-β1 over a period of 24 h. (C) Western blot analysis of SMAD2, SMAD3, p-SMAD2, p-SMAD3, SNAI1, and CCL26 in MFC cells treated with TGF-β1 in the presence or absence of a SMAD2/3 inhibitor (ITD-1). (D) Relative *Ccl26* mRNA levels in MFC cells with SNAI1 knockdown (sh1-SNAI1 and sh2-SNAI1) compared to a negative control (sh-NC). (E) Western blot analysis of CCL26 in GC cells with SNAI1 knockdown (sh1-SNAI1 and sh2-SNAI1) compared to a negative control (sh-NC). (F) Relative *Ccl26* mRNA levels in MFC cells with SNAI1 overexpression (OE-SNAI1) compared to a negative control (OE-NC). (G) Western blot analysis of CCL26 in MFC cells with SNAI1 overexpression (OE-SNAI1) compared to a negative control (OE-NC). (H) Schematic diagram of the *Ccl26* promoter region. Three potential binding sites (B1, B2, and B3) and the locations of these binding sites relative to the transcription start site are shown. (I) Luciferase reporter assay showing the relative promoter activity of WT and mutant (mut) promoters with mutations at 3 binding sites (mut 1, mut 2, and mut 3). (J) ChIP-qPCR analysis showing relative binding of SNAI1 at site 1 compared to an NC region. (K) Schematic illustration of the mechanism that TGF-β1 secreted by PMN-MDSCs promotes CCL26 expression in GC cells through the activation of the SMAD2/3/SNAI1 pathway. Data are presented as mean ± SEM for the bar charts. All groups had *n =* 3 biological replicates. Data of (A) to (C), (I), and (J) were analyzed using 2-way ANOVA followed by Bonferroni’s post hoc test. Data of (D) and (E) were analyzed using one-way ANOVA followed by Bonferroni’s post hoc test. Data of (F) and (G) were analyzed using Student’s *t* test. *P < 0.05, **P < 0.01, ***P < 0.005, ****P < 0.0001, and ns for nonsignificant.

Then, we stimulated MFC cells with TGF-β1 and found that p-SMAD2 and p-SMAD3 protein levels increased in a time- and dose-dependent manner, along with up-regulations of SNAI1 and CCL26 (Fig. 5A and B). When the SMAD2/3 inhibitor ITD-1 was applied [44], the up-regulation of CCL26 by TGF-β1 was abolished (Fig. 5C). The regulatory relationship between SNAI1 and CCL26 was further investigated. Firstly, we observed that both SNAI1 and CCL26 mRNA and protein levels were significantly up-regulated in human GC cell lines compared to the normal gastric epithelial cell line GES-1 (Fig. S11A to D), suggesting a potential regulatory relationship between them. Subsequently, we knocked down SNAI1 in MFC cells (Fig. S12A), which resulted in a significant down-regulation of both *Ccl26* mRNA and its protein levels (Fig. 5D and E). Conversely, overexpression of SNAI1 (OE-SNAI1) in MFC cells (Fig. S12B) led to a notable increase in *Ccl26* mRNA and its protein levels (Fig. 5F and G). These findings suggest that SNAI1 may promote *Ccl26* transcription. To determine whether SNAI1 directly regulates *Ccl26* transcription, we analyzed the *Ccl26* promoter region for potential SNAI1 binding sites (Table S8) and conducted a luciferase reporter assay. The *Ccl26* promoter contains 3 putative SNAI1 binding sites (sites 1 to 3) (Fig. 5H). Mutation analysis of these sites in the luciferase reporter showed that only mutations at site 1 significantly reduced promoter activity in SNAI1-overexpressing cells (Fig. 5I), indicating that this site is crucial for SNAI1-mediated activation of the *Ccl26* promoter. Chromatin immunoprecipitation (ChIP)-qPCR results further confirmed significant enrichment of SNAI1 at site 1 of the *Ccl26* promoter compared to the immunoglobulin G (IgG) control (Fig. 5J), establishing site 1 as the binding site for SNAI1. Together with the findings from Fig. 4, these results suggest that TGF-β1 secreted by CX3CR1^+^ PMN-MDSCs promotes CCL26 expression in GC cells through activation of the SMAD2/3/SNAI1 pathway (Fig. 5K).

### CX3CR1^+^ PMN-MDSCs induced CD8^+^ T cell exhaustion through TGF-β1/mTOR signaling and competitive glucose utilization

**Fig. 6.**
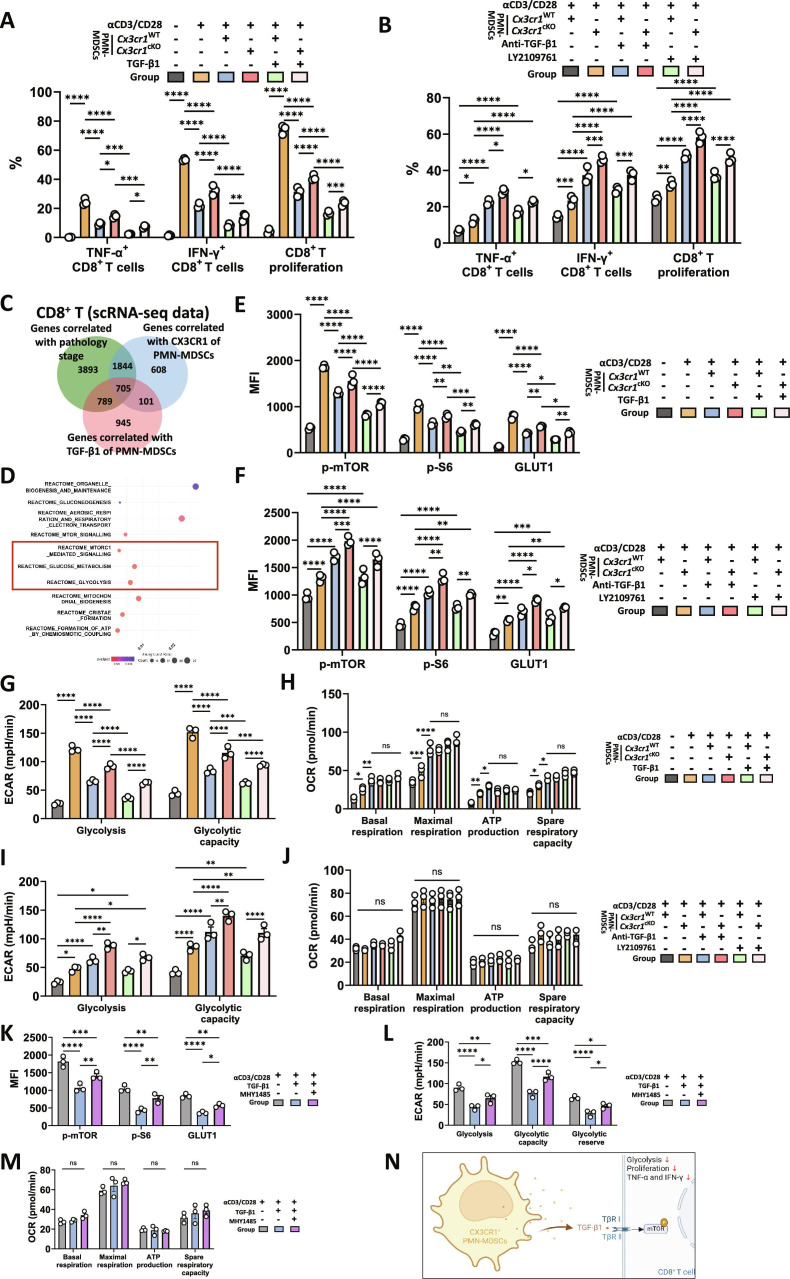
CX3CR1^+^ PMN-MDSCs promoted CD8^+^ T cell exhaustion partially through TGF-β1/mTOR signaling. (A) TNF-α and IFN-γ expression, as well as proliferation (measured by CFSE dilution assay), were assessed in activated CD8^+^ T cells cocultured with *Cx3cr1*^cKO^ and *Cx3cr1*^WT^ PMN-MDSCs, and treated with TGF-β1, compared to unactivated CD8^+^ T cells. The analysis was performed using flow cytometry. (B) TNF-α and IFN-γ expression, as well as proliferation (measured by CFSE dilution assay), were assessed in activated CD8^+^ T cells cocultured with *Cx3cr1*^cKO^ and *Cx3cr1*^WT^ PMN-MDSCs, and treated with either anti-TGF-β1 or the TGFβRI/II inhibitor LY2109761. The analysis was performed using flow cytometry. (C) Venn diagram showing genes significantly correlated with pathology stage, CX3CR1 expression in PMN-MDSCs, and TGF-β1 expression in PMN-MDSCs. Data were derived from 4 scRNA-seq datasets (GEO: GSE134520, GSE183904, GSE150290; dbGaP: phs001818.v2), including a total of *n =* 44 patients. Analyses were focused on the PMN-MDSCs and CD8^+^ T cell. (D) Pathway enrichment analysis highlighting key pathways, with reference to Reactome pathway database. (E) MFI of p-mTOR, p-S6, and GLUT1 in CD8^+^ T cells cocultured with *Cx3cr1*^cKO^ and *Cx3cr1*^WT^ PMN-MDSCs, and treated with TGF-β1, compared to unactivated CD8^+^ T cells. The analysis was performed using flow cytometry. (F) MFI of p-mTOR, p-S6, and GLUT1 in CD8^+^ T cells cocultured with *Cx3cr1*^cKO^ and *Cx3cr1*^WT^ PMN-MDSCs, and treated with either anti-TGF-β1 or the TGFβRI/II inhibitor LY2109761. The analysis was performed using flow cytometry. (G) ECAR values for glycolysis and glycolytic capacity in CD8^+^ T cells cocultured with *Cx3cr1*^cKO^ and *Cx3cr1*^WT^ PMN-MDSCs, and treated with TGF-β1, were compared to those in unactivated CD8^+^ T cells. (H) OCR values for basal respiration, maximal respiration, adenosine triphosphate (ATP) production, and spare respiratory capacity in CD8^+^ T cells cocultured with *Cx3cr1*^cKO^ and *Cx3cr1*^WT^ PMN-MDSCs, and treated with TGF-β1, were compared to those in unactivated CD8^+^ T cells. (I) ECAR values for glycolysis and glycolytic capacity in CD8^+^ T cells cocultured with *Cx3cr1*^cKO^ and *Cx3cr1*^WT^ PMN-MDSCs, and treated with either anti-TGF-β1 or the TGFβRI/II inhibitor LY2109761. (J) OCR values for basal respiration, maximal respiration, ATP production, and spare respiratory capacity in CD8^+^ T cells cocultured with *Cx3cr1*^cKO^ and *Cx3cr1*^WT^ PMN-MDSCs, and treated with either anti-TGF-β1 or the TGFβRI/II inhibitor LY2109761. (K) MFI of p-mTOR, p-S6, and GLUT1 in CD8^+^ T cells treated with TGF-β1 alone or TGF-β1 in combination with the mTOR activator MHY1485. The analysis was performed using flow cytometry. (L) ECAR values for glycolysis and glycolytic capacity in CD8^+^ T cells treated with TGF-β1 alone or TGF-β1 in combination with the mTOR activator MHY1485. (M) OCR values for basal respiration, maximal respiration, ATP production, and spare respiratory capacity in CD8^+^ T cells treated with TGF-β1 alone or TGF-β1 in combination with the mTOR activator MHY1485. (N) Schematic illustration of the mechanism that CX3CR1^+^ PMN-MDSCs promoted CD8^+^ T cell exhaustion through TGF-β1/mTOR signaling in stomach carcinogenesis. Data are presented as mean ± SEM for the bar charts. All groups had *n =* 3 biological replicates. Data of (A), (B), and (E) to (M) were analyzed using 2-way ANOVA followed by Bonferroni’s post hoc test. *P < 0.05, **P < 0.01, ***P < 0.005, ****P < 0.0001, and ns for nonsignificant.

PMN-MDSCs contribute to CD8^+^ T cell exhaustion through cytokine secretion and nutrient competition [14,45]. Herein, we investigated the immunosuppressive role of CX3CR1^+^ PMN-MDSCs. We generated *Cx3cr1^fl/fl^S100a8^cre^* (*Cx3cr1*^cKO^) mice and isolated *Cx3cr1*^cKO^ versus *Cx3cr1*^WT^ PMN-MDSCs for ex vivo assays. CX3CR1 sufficiency enhanced CD8^+^ T cell suppression; this effect was attenuated by TGF-β1 blockade and reduced in *Cx3cr1*^cKO^ PMN-MDSCs, as evidenced by increased tumor necrosis factor-α (TNF-α) and interferon-γ (IFN-γ) production and enhanced CD8^+^ T cell proliferation (Fig. 6A and B). Subsequently, we explored the signaling pathways and biological processes in CD8^+^ T cells influenced by TGF-β1 secreted from CX3CR1^+^ PMN-MDSCs. A total of 705 genes significantly correlated with pathology stage, CX3CR1 expression in PMN-MDSCs, and TGF-β1 expression in PMN-MDSCs were identified (Fig. 6C). Enrichment analysis revealed prominent activation of mTOR signaling, glucose metabolism, and glycolysis pathways (Fig. 6D). Given that mTOR signaling regulates glycolysis, which is critical for T cell activation and effector functions [46], we hypothesized that CX3CR1^+^ PMN-MDSCs suppress CD8^+^ T cells through the TGF-β1/mTOR–glycolysis axis. Consistently, CX3CR1 sufficiency enhanced the suppression of p-mTOR, p-S6, and GLUT1 expression in CD8^+^ T cells (Fig. 6E); this effect was attenuated by TGF-β1 blockade and reduced in *Cx3cr1*^cKO^ PMN-MDSCs (Fig. 6F). Extracellular acidification rate (ECAR)/oxygen consumption rate (OCR) profiling demonstrated TGF-β1-dependent inhibition of CD8^+^ T cell glycolysis through mTOR signaling, whereas mitochondrial respiration remained unchanged (Fig. 6G to M and Fig. S13A to F). These findings indicate that CX3CR1^+^ PMN-MDSCs inhibit glycolysis in CD8^+^ T cells through TGF-β1/mTOR signaling.

However, despite TGF-β1 modulation, both the cytotoxic and proliferative activities (Fig. 6A and B), as well as mTOR activity (Fig. 6E and F) and glycolytic capacity (Fig. 6G and I and Fig. S13A and C) of CD8^+^ T cells cocultured with *Cx3cr1*^WT^ PMN-MDSCs, remained lower than those cocultured with *Cx3cr1*^cKO^ PMN-MDSCs, indicating that additional mechanisms contribute to this suppression. As reported, PMN-MDSCs, being highly glycolytic cells, may further impair T cell function by competing for glucose [47,48]. To investigate this, we added GLUT1 and 2-NBDG readouts to examine glucose utilization in CX3CR1^+^ PMN-MDSCs and CD8^+^ T cells, revealing the reduced glucose uptake in *Cx3cr1*^cKO^ PMN-MDSCs and a reciprocal increase in CD8^+^ T cells; TGF-β1 blockade selectively restored glucose uptake in CD8^+^T cells (Fig. S14A and B).

Taken together, CX3CR1^+^ PMN-MDSCs not only suppress CD8^+^ T cell glycolysis through TGF-β1/mTOR signaling (Fig. 6N) but also directly compete for glucose (Fig. S14C) in the GC TIME.

### Specific knockout of CX3CR1 on PMN-MDSCs combined with αPD-1 inhibited GC formation

We generated a GC model in C57BL/6J mice by implanting MFC cells (Fig. S15A) and observed elevated levels of CCL26 in the plasma (Fig. S15B). Consistently, both spleen and tumor tissues from MFC tumor-bearing mice showed significant accumulation of total MDSCs and their subsets (Fig. S15C). CX3CR1 up-regulation was detected in splenic and tumor-infiltrating MDSCs across both murine tumor models. In MFC tumor-bearing mice, CX3CR1 expression was markedly up-regulated in MDSCs and PMN-MDSCs within the spleen and tumor tissues compared to the spleens of naïve mice, whereas no significant changes were observed in M-MDSCs (Fig. S15D).

To investigate the function of CX3CR1, *Cx3cr1^−/−^* mice were generated (Fig. S16A to C). These mice showed reduced tumor growth and weight compared to WT mice (Fig. S16D and E). In the tumor-bearing state, *Cx3cr1^−/−^* mice exhibited a significant reduction in total MDSCs and their subsets in both spleen and tumor (Fig. S16F and G), along with down-regulation of tumor-associated macrophages (TAMs), while DCs remained unaffected (Fig. S16H and I). The reduction in MDSCs, especially PMN-MDSCs, was most pronounced. Conversely, CX3CR1 knockout increased the percentage of CD8^+^ T cells and IFN-γ^+^ TNF-α^+^ CD8^+^ T cells (Fig. S16J and K).

**Fig. 7.**
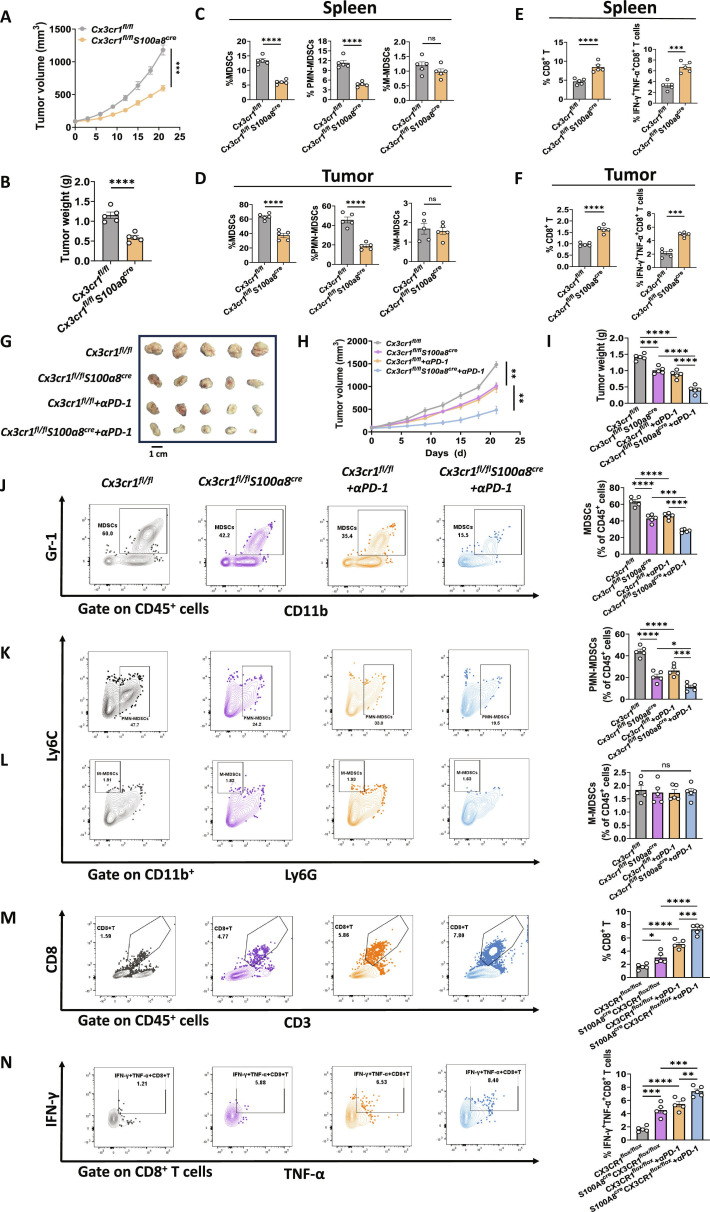
Specific knockout of CX3CR1 in PMN-MDSCs combined with anti-PD-1 inhibited GC formation. (A and B) Comparison of tumor volumes (A) and weights (B) from *Cx3cr1^fl/fl^* and *Cx3cr1^fl/fl^S100a8^cre^* mice. (C) Flow cytometry analysis of CX3CR1 expression on splenic MDSCs from *Cx3cr1^fl/fl^* and *Cx3cr1^fl/fl^S100a8^cre^* mice. (C and D) Comparison of the percentage of MDSCs, PMN-MDSCs, and M-MDSCs in the spleens (C) and tumors (D) of *Cx3cr1^fl/fl^* and *Cx3cr1^fl/fl^S100a8^cre^* mice. (E and F) Comparison of the percentage of CD8^+^ T cells and IFN-γ^+^ TNF-α^+^ CD8^+^ T cells in the spleens (E) and tumors (F) of *Cx3cr1^fl/fl^* and *Cx3cr1^fl/fl^S100a8^cre^* mice. (G) Image showing tumors from *Cx3cr1^fl/fl^*, *Cx3cr1^fl/fl^S100a8^cre^* mice, and those treated with αPD-1. (H and I) Comparison of tumor volumes (H) and weights (I) from *Cx3cr1^fl/fl^*, *Cx3cr1^fl/fl^S100a8^cre^* mice, and those treated with αPD-1. (J to N) Flow cytometry plots and corresponding quantitative analysis of MDSCs (J), PMN-MDSCs (K), M-MDSCs (L), CD8^+^ T cells (M), and IFN-γ^+^ TNF-α^+^ CD8^+^ T cells (N) in tumors of *Cx3cr1^fl/fl^*, *Cx3cr1^fl/fl^S100a8^cre^* mice, and mice treated with αPD-1. Data are presented as mean ± SEM for the bar charts. All groups had *n =* 5 biological replicates. Data of (A) to (F) and (H) were analyzed using Student’s *t* test. Data of (I) to (N) were analyzed using one-way ANOVA followed by Bonferroni’s post hoc test. *P < 0.05, **P < 0.01, ***P < 0.005, ****P < 0.0001, and ns for nonsignificant.

To further investigate the role of CX3CR1 in PMN-MDSCs, we crossed *Cx3cr1^fl/fl^* mice with *S100a8^cre^* mice to generate *Cx3cr1^fl/fl^S100a8^cre^* mice (Fig. S17A and B). Sorted PMN-MDSCs from *Cx3cr1^fl/fl^S100a8^cre^* tumor-bearing mice show marked *Cx3cr1* reduction at mRNA and surface protein levels (qPCR, flow MFI; Fig. S17C and D). These data verify efficient, lineage-relevant deletion. Similar to *Cx3cr1^−/−^* mice, CX3CR1 knockout in PMN-MDSCs inhibited tumor growth (Fig. 7A and B) and reduced both total MDSCs and PMN-MDSCs proportions (Fig. 7C and D), while M-MDSCs remained unchanged. CD8^+^ T cells and IFN-γ^+^ TNF-α^+^ CD8^+^ T cells were up-regulated (Fig. 7E and F). These findings suggest that CX3CR1 deficiency in PMN-MDSCs limits their infiltration and enhances the cytotoxic function of CD8^+^ T cells in GC.

Recent studies have reported PD-1 up-regulation in the stomach during tumorigenesis [49]. Based on scRNA-seq and flow cytometry analysis, we observed that PD-1 expression on T cells was up-regulated during stomach carcinogenesis (Fig. S18A to E). Since PD-1 is known to inhibit T cell activation and our finding that CX3CR1 is essential for PMN-MDSCs migration and CD8^+^ T cell suppression, we hypothesized that dual blockade of PD-1 and CX3CR1 may enhance anti-GC efficacy. Tumor-bearing *Cx3cr1^fl/fl^S100a8^cre^* and *Cx3cr1^fl/fl^* mice were treated with or without αPD-1 antibody, with *Cx3cr1^fl/fl^S100a8^cre^* mice treated with αPD-1 showing smaller tumor volumes, slower growth, and reduced tumor weights compared to those receiving single treatments (Fig. 7G to I), indicating a synergistic effect between CX3CR1 knockout and PD-1 blockade. Flow cytometry analysis showed that compared with the *Cx3cr1^fl/fl^* group, the percentage of MDSCs and PMN-MDSCs were significantly down-regulated in the *Cx3cr1^fl/fl^S100a8^cre^*, *Cx3cr1^fl/fl^* + αPD-1, and *Cx3cr1^fl/fl^S100a8^cre^* + αPD-1 groups, with the combination therapy showing the most pronounced reduction (Fig. 7J and K and Fig. S19A and B). However, the proportion of M-MDSCs remained unchanged (Fig. 7L and Fig. S19C). Concurrently, the percentage of CD8^+^ T cells and IFN-γ^+^ TNF-α^+^ CD8^+^ T cells were most significantly up-regulated in the combination therapy group (Fig. 7M and N and Fig. S19D and F). These results suggest that combining CX3CR1 inhibition in PMN-MDSCs with αPD-1 treatment synergistically improves antitumor immunity in GC.

## Discussion

This study identified the CCL26–CX3CR1 axis as a regulator of PMN-MDSCs infiltration to the TIME during stomach carcinogenesis. CCL26, secreted by GC cells, chemotaxed PMN-MDSCs through binding to CX3CR1, which in turn enhanced CCL26 expression via the TGF-β1/SMAD2/3/SNAI1 signaling pathway. This self-reinforcing cycle enabled persistent infiltration of immunosuppressive PMN-MDSCs, triggering CD8^+^ T cell exhaustion via the TGF-β1/mTOR pathway and fostering tumorigenesis. Therapeutically, CX3CR1 inhibition in PMN-MDSCs within a mouse GC model substantially diminished their prevalence and potentiated anti-PD-1 therapy response.

Substantial evidence underscores the pivotal function of immune cell infiltration in the TIME of stomach carcinogenesis [50]. For instance, activation of the β-catenin/T cell factor (TCF) signaling pathway results in CCL28 up-regulation, which subsequently triggers the infiltration of Tregs, thereby enhancing immune suppression within the GC microenvironment [51]. Additionally, GC cells secrete chemokines such as monocyte chemoattractant protein-1 (MCP-1/CCL2) and macrophage inflammatory protein-2α (MIP-2α/CXCL2), mediating the recruitment of CD11B^+^ myeloid cells from the bloodstream into the TIME [52]. These recruited cells then evolve into TAMs characterized by high expression of anti-inflammatory mediators [interleukin-10 (IL-10) and IL-4], fostering an immune-evasive niche [52–54]. Furthermore, during the development of spasmolytic polypeptide-expressing metaplasia, Sonic hedgehog signaling induced the infiltration of SLFN4^+^ MDSCs into the stomach [55]. These MDSCs secreted regulatory miR-130b to suppress T cell activity [56]. In this research, we identified CCL26 as being up-regulated during stomach carcinogenesis and found that it chemotactically recruited PMN-MDSCs through CX3CR1. CCL26, also known as eotaxin-3, is a member of the CC chemokine family that was initially identified as a chemoattractant for eosinophils through CCR3 binding [57], but recently has also been recognized as a functional ligand for CX3CR1 [30,58], aligning with our findings. In oral squamous cell carcinoma (OSCC) [59] and colorectal cancer (CRC) [60], tumor cells exhibit high expression of CCL26, with elevated levels promoting epithelial–mesenchymal transition (EMT) and correlating with poorer survival. In Hodgkin lymphoma and cutaneous T cell lymphoma (CTCL), CCL26 recruits CCR3^+^ T lymphocytes into TIME, where they secrete high levels of IL-4—a hallmark of a T helper 2 (Th2)-dominant microenvironment that is closely associated with CTCL development [61]. These findings underscore the broader role of CCL26 in cancer biology and tumor immunity. CX3CR1 has been identified as a poor prognostic factor linked to metastasis in multiple malignancies, including GC [62,63]. A recent study demonstrated that CX3CR1^+^ PMN-MDSCs are markedly up-regulated in the intraperitoneal TIME following GC surgery [64], where they exhibit immunosuppressive activity and foster tumor progression. Based on our findings of a tumor cell-derived CCL26 and CX3CR1^+^ PMN-MDSCs interaction axis in GC, we propose that this pathway holds translational potential both as a noninvasive biomarker for stomach carcinogenesis, potentially reducing the need for repeated endoscopic examinations, and as a therapeutic target to disrupt tumor-myeloid cross-talk, thereby limiting immunosuppression and tumor progression. However, diagnostic thresholds across disease stages remain to be established. Meanwhile, CX3CR1 is also expressed on CD8^+^ T cells and NK cells, where it mediates recruitment and cytotoxic activation via CX3CL1 and is linked to improved patient outcomes [65]. Thus, future clinical interventions should be designed to selectively target CX3CR1^+^ PMN-MDSCs, in order to avoid impairing antitumor immunity while maximizing therapeutic benefit.

PMN-MDSCs exhibit complex crosstalk with both cancer cells and T cells [21,66]. Notably, TGFB1, TGFB2, TGFB3, S100A8, S100A9, IL6, and PTGES secreted by PMN-MDSCs exhibit both protumorigenic and immunosuppressive properties [36,67–69], playing significant roles in promoting tumor growth and evading immune responses. We found that CX3CR1^+^ PMN-MDSCs primarily secreted TGF-β1, which interfered with the GC TIME. TGF-β1 is a key cytokine involved in various cellular processes, and its signaling pathways play a crucial role in cancer progression and immune regulation [70]. For examples, in lung adenocarcinoma, Hdc^+^ PMN-MDSCs express TGF-β1, which induces EMT in cancer cells at metastatic sites [71]. Similarly, in melanoma mouse models, PMN-MDSCs were found to express TGF-β1, facilitating EMT [72]. In ovarian cancer, PMN-MDSCs have also been reported to release TGF-β1 and may present a considerable obstacle to the effectiveness of antitumor responses elicited by immunotherapy [73]. However, the role of TGF-β1 in stomach carcinogenesis remains unclear, necessitating further investigation. Pathway enrichment and experimental analysis revealed that TGF-β1 up-regulates p-SMAD2/3 and SNAI1 in GC cells. It has been previously reported that TGF-β1 binds to its receptors, which activates SMAD2 and SMAD3 through phosphorylation by TGF-β receptors I and II. Upon phosphorylation, SMAD2 and SMAD3 create trimeric complexes with SMAD4, subsequently undergoing nuclear translocation where they collaborate with DNA-binding transcriptional factors to modulate SNAI1 expression for EMT promotion [74,75]. Contemporary research has additionally emphasized SNAI1’s function in immune regulation. For example, SNAI1 transcribes CXCL1 and CXCL2 to regulate MDSCs infiltration in breast cancer [76]. In present study, we found that TGF-β1 released by PMN-MDSCs activated SNAI1 in GC cells via SMAD2/3. SNAI1 then transcribed CCL26 by directly binding to its promoter, leading to its up-regulation and the chemotaxis of PMN-MDSCs. These observations indicate the existence of a positive feed-forward regulatory circuit between GC cells and PMN-MDSCs across stomach carcinogenesis.

PMN-MDSCs are suppressive regulatory factors that can impair CD8^+^ T cell function [21]. As one of the effector molecules produced by PMN-MDSCs, TGF-β1 negatively impacts cytotoxic T cell functionality [77,78]. During stomach carcinogenesis, we observed that TGF-β1 secreted by PMN-MDSCs inhibited CD8^+^T proliferation and cytotoxic activity. scRNA-seq analysis and experimental results suggested that TGF-β1 led to CD8^+^ T cell exhaustion by impairing mTOR signaling and glycolysis. It has been reported that the cytotoxicity of CD8^+^ T cells is primarily dependent on glycolysis [79] and mTOR serves as a central metabolic regulator, responding to both intracellular and extracellular signals to adapt cellular metabolism [80]. High mTOR activity promotes aerobic glycolysis, which supports rapid cell growth and immune function by generating key metabolic intermediates [81]. This process is primarily mediated by mTOR’s regulation of Myc and HIF-1α, which activate downstream transcriptional programs critical for glycolysis [82]. Research has demonstrated that TGF-β treatment of CD8^+^ T cells leads to reduced mTOR activation and impairs the expression of glycolytic genes [83,84]. Inhibition of mTORC1 in IL-2-maintained cytotoxic T lymphocytes also results in reduced glycolysis [85]. Moreover, loss of mTORC1 activity has been associated with functional and metabolic decline in exhausted T cells [86–88]. Similarly, TGF-β limits glycolysis in NK cells by down-regulating mTOR activity [89]. These observations highlight the essential role of TGF-β-mTOR signaling in suppressing the metabolic activity and functional capacity of cytotoxic immune cells. Our study found that CX3CR1^+^ PMN-MDSCs promote CD8^+^ T cell exhaustion, partially through the TGF-β1/mTOR signaling pathway. However, whether TGF-β1 was added or its signaling was blocked, the immunosuppressive function of *Cx3cr1*^CKO^ PMN-MDSCs on CD8^+^ T cells remained consistently weaker than that of *Cx3cr1*^WT^ PMN-MDSCs, suggesting the involvement of additional mechanisms. Previous reports indicate that PMN-MDSCs, as highly glycolytic cells, can suppress T cell function by directly competing for glucose [47,48], which was also confirmed in our findings. These results highlight that CX3CR1^+^ PMN-MDSCs inhibit CD8^+^ T cells through multiple pathways.

It has been reported that PD-1 expression increases during stomach carcinogenesis [49], and TGF-β1 has been shown to up-regulate PD-1 expression on CD8^+^ T cells via the SMAD3 pathway [90]. This suggests the potential necessity of PD-1 blockade. As reported by Kim et al. [49], early anti-PD-1 treatment inhibited gastric tumor growth, reduced MDSCs, and triggered robust antitumor immunity in gastrin-deficient (GAS-KO) mice. However, the same treatment was ineffective in late-stage tumors, which were characterized by a robust infiltration of PMN-MDSCs, correlating with a lack of response to anti-PD-1 therapy. They proposed that combining PD-1 blockade with MDSC-targeting strategies may help overcome resistance to immune checkpoint blockade. Complementarily, our findings identified CX3CR1 as a novel target for PMN-MDSCs intervention, supporting the efficacy of combining PD-1 blockade with targeting PMN-MDSCs in stomach carcinogenesis. However, several critical issues must be addressed when considering the future application of CX3CR1-targeted strategies in combination with PD-1 blockade for GC. First, no CX3CR1 inhibitors are approved for oncology currently, and existing preclinical data rely on murine-specific antibodies [91], underscoring the need for fully humanized agents and rigorous safety testing. Second, as CX3CR1 is expressed on monocytes, NK cells, and microglia, systemic inhibition may cause off-target toxicities [92]. Stratification with biomarkers such as plasma CCL26 or intratumoral CX3CR1^+^ PMN-MDSCs density could help identify patients most likely to benefit. Third, the optimal treatment schedule remains undefined. While concurrent administration has been tested in preclinical models [91], sequential approaches could be further explored to better balance efficacy with safety. Fourth, GC comprises 4 molecular subtypes—Epstein–Barr virus (EBV)-positive, microsatellite instability (MSI), chromosomal instability (CIN), and genomically stable (GS)—each with distinct immune features [93,94]; identifying the subgroups most likely to benefit is therefore essential. Finally, because CX3CL1–CX3CR1 signaling can also recruit and activate effector T cells and NK cells [65,95], indiscriminate blockade of CX3CR1 may blunt beneficial antitumor immunity. Taken together, the clinical translation of CX3CR1 and PD-1 combination therapy will require a biomarker-driven, personalized approach with careful patient selection, dosing, and scheduling to maximize therapeutic benefit while minimizing risk.

In this research, we also observed that anti-PD-1 treatment alone could also lead to a reduction in MDSCs. One possible explanation is that anti-PD-1 treatment activates CD8^+^ T cells, enhancing their cytotoxic activity against tumor cells, which reduces tumor burden and consequently inhibits tumor cell-mediated MDSCs migration [96]. Another possibility is that MDSCs express PD-1 [97,98], and anti-PD-1 treatment may induce enhanced activation of extracellular signal-regulated kinase (ERK1/2), mTORC1, and signal transducer and activator of transcription 1 (STAT1) in GMPs, which serve critical roles in myeloid cell differentiation and maturation while blocking the formation of immature, immunosuppressive MDSCs [99–102].

In summary, CCL26 secreted by GC cells binds to CX3CR1 and promotes the infiltration of PMN-MDSCs into the TIME. This infiltration further amplifies CCL26 production through the TGF-β1/SMAD2/3/SNAI1 signaling pathway. Additionally, CX3CR1^+^ PMN-MDSCs secreted TGF-β1 to suppress mTOR signaling and simultaneously compete with them for glucose, disrupting glycolysis and leading to T cell exhaustion. Importantly, inhibition of CX3CR1 in PMN-MDSCs significantly reduces their accumulation and enhances the effectiveness of anti-PD-1 therapy, thereby inhibiting stomach carcinogenesis. These findings suggest that targeting PMN-MDSCs and their receptor CX3CR1 may represent a promising strategy for the prevention of stomach carcinogenesis.

## Materials and Methods

### Reagents and antibodies

The reagents and antibodies used in this research were listed in Table S1.

### Patient population

All patient samples were obtained from the First Affiliated Hospital of Guangzhou University of Chinese Medicine, China. The diagnostic criteria of CNAG, CAG, and GC were based on previous studies [103,104]. All human studies were sanctioned by the ethics committee of the First Affiliated Hospital of Guangzhou University of Chinese Medicine (K-2023-086). Written informed consent was procured from all patients. The clinical characteristics of recruited patient were shown in Table S2.

### Peripheral blood mononuclear cell isolation and plasma collection

Following ethics committee approval, participants including patients with CNAG, CAG, and GC, alongside healthy donors, were enrolled at the First Hospital of Guangzhou University of Chinese Medicine between July 2023 and July 2024. Blood samples of 2 ml were procured from each participant. Peripheral blood mononuclear cells were procured from fresh blood specimens through Ficoll–Hypaque density gradient centrifugation methodology. Plasma specimens were harvested and preserved at −80 °C for subsequent analysis.

### Cell culture

MFC (catalog no.: CL-0156, RRID:CVCL_5J48), AGS (catalog no.: CL-0022, RRID:CVCL_0139), HGC-27 (catalog no.: CL-0107, RRID:CVCL_1279), MKN-45 (catalog no.: CL-0292, RRID:CVCL_0434), and NCI-N87 (catalog no.: CL-0169, RRID:CVCL_1603) cell lines were acquired from Wuhan Procell Life Science & Technology Co. Ltd. on 2023 September 1. GES-1 (catalog no.: ZQ0905, RRID:CVCL_EQ22) was purchased from Shanghai Zhong Qiao Xin Zhou Biotechnology Co. Ltd on 2023 September 1. All cell lines were confirmed to be free from contamination. AGS cells were maintained in Ham’s F-12 medium. GES-1 cells were maintained in Dulbecco’s modified Eagle’s medium (DMEM). MFC, HGC-27, MKN-45, and NCI-N87 cells were maintained in RPMI 1640 medium. All culture media were composed of 10% fetal bovine serum (FBS) (Gibco) and 1% penicillin–streptomycin. Cell cultures were maintained in a humidified atmosphere containing 5% CO_2_ at 37 °C. Multiple factors were considered when selecting MFC, AGS, HGC-27, MKN-45, NCI-N87, and GES-1 as experimental cell lines. MFC represents a cell line derived from forestomach carcinoma that developed in *N*-methyl-*N*′-nitro-*N*-nitrosoguanidine (MNNG)-treated mice, frequently utilized in GC research studies. It has good tumorigenicity, including in C57BL/6J mice with Cre-LoxP system [105], which facilitates in vivo experiments. GES-1 represents a human gastric epithelial cell line frequently employed as a control when compared to GC cell lines. AGS, HGC-27, MKN-45, and NCI-N87 represent human GC cell lines exhibiting epithelial cell characteristics, frequently utilized in GC-related experiments.

### Establishment of knockdown/overexpression cell lines

To stably knock down SNAI1 and CCL26, short hairpin RNA (shRNA) targeting *Snai1* (mouse, sh1#: 5′-GCCACCTTCTTTGAGGTACAA-3′ and sh2#: 5′-CCACTCGGATGTGAAGAGATA-3′) and CCL26 (human, sh1#: 5′-GCTGTGATATTCACTACCAAA-3′ and sh2#: 5′-CTGCTTCCAATACAGCCACAA-3′) and a negative control (NC) shRNA (5′-GGGCGAGGAGCTGTTCACCG-3′) were designed, synthesized, and cloned into the pFB-8 vector by Focus Bioscience Inc. (Shanghai, China). This vector stably expresses shRNA and a puromycin resistance gene along with pMD2.G and psPAX2. pFB-8, pMD2.G, and psPAX2 were mixed together and introduced into human embryonic kidney (HEK)-293T cells utilizing Lipofectamine 2000 (Invitrogen, Shanghai, China) per the supplier’s protocol. Following a 48-h transfection period, the recombinant viruses were then harvested.

To stably overexpress SNAI1 and CCL26, full-length SNAI1 and CCL26 cDNA were cloned into the pSin-puro vector. Lentiviral transfer plasmids underwent cotransfection with pMD.2G and psPAX2 in HEK-293T cells for 48 h to achieve virus packaging. The resulting recombinant viruses were harvested, and following polybrene-mediated infection, puromycin selection was employed to isolate positive clones. The established stable cell lines included sh1-SNAI1, sh2-SNAI1, sh1-CCL26, sh2-CCL26, sh-NC, OE-SNAI1, OE-CCL26, and OE-NC.

### Animal studies

WT C57BL/6J mice, *Cx3cr1* knockout (*Cx3cr1^−/−^*) mice, and *Cx3cr1^fl/fl^* mice were obtained from Cyagen Biosciences. Recent studies have highlighted S100A8 as a distinctive marker for PMN-MDSCs [35,106]. To generate mice with specific *Cx3cr1* deletion in PMN-MDSCs, *Cx3cr1^fl/fl^* mice were crossed with *S100a8^cre^* mice and generated *Cx3cr1^fl/fl^S100a8^cre^* mice. All animal research protocols were sanctioned by the Institutional Animal Ethics Committee of Guangzhou University of Chinese Medicine. MFC cells (1 × 10^7^) were injected subcutaneously into the flanks of mice. Tumor development was assessed every 3 days, with tumor volume determined through the following equation: *V* = *D* × *d*^2^ (*V*, volume; *D*, longest diameter; *d*, shortest diameter). Animals underwent euthanasia upon experiment completion. αPD-1 (RMP1-14, BioXCell) was prepared in 200 μl of phosphate-buffered saline (PBS) and delivered intraperitoneally at 200 μg per dose twice weekly.

### Preparation of single-cell suspensions from mouse tissues

Single-cell suspensions were prepared from spleen and tumor samples. Spleens were excised and mechanically dissociated through 70-μm cell strainers (Biosharp). Following centrifugation, splenocyte suspensions underwent erythrocyte depletion using red blood cell (RBC) lysis buffer (Biosharp). Tumor tissues were minced into fragments and enzymatically digested in 2 ml of RPMI 1640 (Gibco) containing 1 mg/ml collagenase IV (Biosharp) at 37 °C for 3 h. Resulting suspensions were filtered through 70-μm strainers, followed by RBC elimination with lysis buffer.

### Flow cytometry and fluorescence-activated cell sorting

Specimens were pre-incubated with anti-CD16/32 (BD Pharmingen) at 4 °C for 30 min to prevent nonspecific binding. Subsequent surface antigen detection involved direct antibody staining. For intracellular TNF-α/IFN-γ analysis, cells underwent stimulation with a cocktail containing protein transport inhibitor (TONBO), followed by fixation/permeabilization kit (eBioscience) and intracellular antibody labeling. Indirect detection utilized primary antibodies plus phycoerythrin (PE)-conjugated goat anti-rabbit IgG secondary antibodies (Abcam). Samples underwent analysis using a BD LSRFortessa (BD Biosciences), BD FACSVerse (BD Biosciences), or FACSAria III cell sorter (BD Biosciences) with BD FACSDiva software (v9.0, BD Biosciences). FlowJo V10 software (TreeStar) was utilized for data analysis.

### In vitro coculture of PMN-MDSCs and MFC cells

*Cx3cr1*^cKO^ and *Cx3cr1*^WT^ PMN-MDSCs were isolated from the spleens of MFC-bearing *Cx3cr1^fl/fl^S100a8^cre^* and *Cx3cr1^fl/fl^* mice, respectively, with tumor volumes matched across groups. The *Cx3cr1*^cKO^ and *Cx3cr1*^WT^ PMN-MDSCs were cocultured with the MFC cell line at a 1:1 cell ratio in a Transwell device (24-well, 0.4-μm pore size), with PMN-MDSCs in the upper chamber and MFC cells in the lower chamber for 48 h. When indicated, the following treatments were applied: TGF-β1 (10 ng/ml, R&D Systems), LY2109761 (a TGF-βRI/II inhibitor, 5 μM, Selleck), or anti-TGF-β1 neutralizing antibody (1 μg/ml, BioLegend). After the coculture period, MFC cells were digested for further analysis.

### CD8^+^ T cell suppression assay

CD8^+^ T cells underwent activation through anti-CD3/CD28-coated Dynabeads (Thermo Fisher Scientific). For proliferation assessment, cells received labeling with carboxyfluorescein succinimidyl ester (CFSE; TONBO). Cells were cocultured with *Cx3cr1*^cKO^ and *Cx3cr1*^WT^ PMN-MDSCs at a 1:1 ratio in Transwell plates (24-well, 0.4-μm pore size) for 48 h, with PMN-MDSCs in the upper chamber and CD8^+^ T cells in the lower chamber. In some conditions, additional treatments were applied: TGF-β1 (10 ng/ml, R&D Systems), LY2109761 (a TGF-βRI/II inhibitor, 5 μM, Selleck), anti-TGF-β1 neutralizing antibody (1 μg/ml, BioLegend), and MHY1485 (an mTORC1 activator, 10 μM, Selleck). Flow cytometry was used to measure changes in CFSE signal, the proportion of CD8^+^ T cells producing TNF-α and IFN-γ, and the MFI of p-mTOR, p-S6, and GLUT1. For glucose uptake measurement, 2-(*N*-(7-nitrobenz-2-oxa-1,3-diazol-4-yl)amino)-2-deoxyglucose (2-NBDG) was added directly to the coculture wells for the last hour of the experiment at a final concentration of 35 μM. Following incubation, cells were harvested, extensively washed, and subsequently analyzed for 2-NBDG fluorescence intensity using flow cytometry.

### Transwell migration assay

Cell migration was evaluated utilizing Transwell plates (24-well plates, 3-μm pore size). PMN-MDSCs (1 × 10^5^) from patients with GC were loaded in the upper chamber and subjected to various treatments: 0% FBS (NC), CCL26 (PeproTech), CCL26 with anti-CX3CR1 (Torrey Pines), CCL26 with anti-CCR3 (R&D Systems), and AGS or modified AGS cells (sh26#1, sh26#2, and CCL26-OE). Additionally, PMN-MDSCs (1 × 10^5^) from the spleens of WT and *Cx3cr1^−/−^* tumor-bearing mice were also tested for CCL26-directed migration. After 24-h incubation (37 °C, 5% CO₂), transmigrated cells were enumerated.

### ChIP assay

MFC cells underwent incubation with 1% formaldehyde at 37 °C for 10 min, with cross-linking termination achieved by adding 125 mM glycine followed by an additional 10-min incubation. Following pelleting, cells were washed using PBS and lysed using cell lysis buffer, and lysates underwent sonication to fragment chromatin into DNA pieces. The extracts underwent overnight incubation at 4 °C with 2 μg of SNAI1 antibody (Cell Signaling) for the experimental group or with 1 μg of control IgG for the NC group. Immunocomplexes underwent precipitation using protein G agarose beads, followed by washing, elution, and dissociation. The purified DNA obtained from protein–DNA complexes served as a template in qPCR for amplifying the target DNA.

### Luciferase assay

HEK-293 cells were plated in 6-well plates at a density of 2 × 10^5^ cells per well and subjected to transfection with 0.8 μg of luciferase reporter plasmid combined with 8 ng of pRL-CMV that encodes *Renilla* luciferase. Following a 36-h transfection, luciferase activity was quantified utilizing the Dual-Luciferase Kit (Promega).

### Hematoxylin and eosin staining

Gastric mucosa tissue specimens from patients diagnosed with CNAG, CAG, and GC were obtained and preserved in 10% neutral buffered formalin, followed by dehydration using a graduated series of alcohol solutions. The tissues were then paraffin-embedded and cut into 4-μm-thick sections. Hematoxylin and eosin (H&E) staining was applied to these sections, which were then subjected to microscopic examination for histological evaluation.

### Immunofluorescence

Formalin-fixed and paraffin-embedded gastric mucosa specimens were sectioned at 4-μm thickness, subsequently subjected to rehydration, antigen retrieval, and blocking procedures. The slides underwent overnight incubation with primary antibodies at 4 °C, followed by treatment with horseradish peroxidase-conjugated secondary antibodies for 50 min at room temperature. Fluorophore-conjugated tyramides were then applied for signal amplification in multiplex immunofluorescence. After antibody stripping and antigen retrieval, subsequent rounds of antibodies were incubated. Nuclei underwent counterstaining with 4′,6-diamidino-2-phenylindole (DAPI), followed by mounting of slides with antifade medium and coverslip placement before fluorescence microscopic examination. Image analysis was performed using ImageJ software.

### Western blotting

Protein extraction was performed using radioimmunoprecipitation assay (RIPA) lysis buffer supplemented with protease and phosphatase inhibitor cocktail. Protein concentrations were determined using a bicinchoninic acid (BCA) kit (CWBIO) and normalized with RIPA buffer. The supernatant was then combined with loading buffer and boiled at 100 °C for 5 min. Protein separation was achieved on sodium dodecyl sulfate (SDS)-polyacrylamide gels followed by electrotransfer to polyvinylidene difluoride (PVDF) membranes. Membrane blocking was conducted with Quick Block blocking buffer for 1 h, followed by overnight incubation at 4 °C with primary antibodies (1:1,000 dilution), and subsequent incubation with secondary antibodies for 1 h at room temperature. After additional tris-buffered saline with Tween (TBST) washing, membranes were treated with developing solution and visualized using a high-sensitivity chemiluminescence imaging system. Results were analyzed using ImageJ software.

### RNA extraction and qPCR

Total RNA was extracted from cells using the EZ-press RNA Purification Kit (EZBioscience). Reverse transcription was performed using 4× Reverse Transcription Master Mix (EZBioscience), and qPCR was performed using 2× SYBR Green qPCR Master Mix (EZBioscience), per the supplier’s protocols.

### Enzyme-linked immunosorbent assay

Plasma/supernatant was assessed utilizing Human CCL26, Human TGF-β1, and Mice CCL26 Quantikine ELISA Kits (MEIMIAN), per the supplier’s protocols.

### RNA sequencing

PMN-MDSCs isolated from the peripheral blood of CNAG, CAG, and GC patients, as well as from the tumor tissue of MFC-bearing WT and *Cx3cr1^−/−^* mice, were subjected to RNA extraction using TRIzol reagent (Invitrogen). The quality and concentration of total RNA samples were evaluated utilizing an Agilent 2100 RNA Nano 6000 Assay Kit (Agilent Technologies, Santa Clara, CA, USA).

Purified mRNA was fragmented, after which first-strand cDNA synthesis occurred using random hexamer primers. Second-strand synthesis utilized buffer, deoxynucleotide triphosphates (dNTPs), ribonuclease (RNase) H, and DNA polymerase I, with subsequent purification performed using QIAQuick PCR kits and EB buffer elution. The double-stranded cDNA experienced end repair, A-base addition, and sequencing adaptor ligation, then was recovered through agarose gel for target fragments, and PCR amplified to finalize library preparation.

Following library construction, Qubit 2.0 was employed for initial quantification, with the library diluted to 1 ng/μl. Agilent 2100 confirmed the insert size, and upon meeting expectations, qPCR was utilized to precisely determine the effective concentration (>10 nM) for quality assurance. Qualified libraries were mixed based on required concentration for Illumina HiSeq PE150 sequencing and target data yield.

### DIA proteomic analysis

An appropriate number of biological samples were weighed and transferred into 2-ml centrifuge tubes, to which steel balls and a lysis solution containing 8 M urea, 50 mM tris–HCl, and protease inhibitor cocktail (Roche, final concentration 1×) were added. The tubes were placed on ice for 5 min before homogenization using a tissue lyser at 60 Hz for 2 min. The homogenates were then centrifuged at 20,000*g* for 15 min at 4 °C, and the supernatants were collected. Dithiothreitol (DTT) was added to each tube to a final concentration of 10 mM, followed by incubation in a 37 °C water bath for 1 h. Then, iodoacetamide (IAA) was added to a final concentration of 20 mM, and the tubes were incubated in the dark for 30 min. For protein quantification, the Bradford method was used: 0, 2, 4, 6, 8, 12, 14, 16, and 18 μl of bovine serum albumin (BSA) standard solution (0.2 μg/μl) were added into wells of a 96-well plate, followed by 20, 18, 16, 14, 12, 10, 8, 6, 4, and 2 μl of pure water, respectively, to make up a constant volume. After thorough mixing, 180 μl of Coomassie Brilliant Blue G-250 working solution was added to each well. Absorbance at 595 nm (OD_595_) was measured using a microplate reader, and a standard curve was constructed. For sample measurements, 20 μl of protein solution was mixed with 180 μl of the same working solution, and the OD_595_ values were recorded to calculate the protein concentration based on the standard curve. To evaluate protein integrity, SDS-polyacrylamide gel electrophoresis (PAGE) was performed: 10 μg of protein from each sample was mixed with an appropriate volume of loading buffer, boiled at 95 °C for 5 min, and centrifuged at 20,000*g* for 5 min. The supernatant was loaded into the wells of a 4% to 12% SDS-PAGE gel and electrophoresed at 80 V for 20 min, followed by 120 V for 60 min. After electrophoresis, the gel was stained, decolorized, and imaged. For digestion, 150 μg of protein per sample was taken and mixed with 3 μg of trypsin to achieve a protein-to-enzyme ratio of 50:1, and digestion was carried out at 37 °C for 14 to 16 h. The resulting peptides were desalted using Waters solid-phase extraction cartridges, vacuum-dried, and reconstituted in pure water before storage at −20 °C. For DIA quantification, dried peptides were reconstituted in 0.1% formic acid and centrifuged at 20,000*g* for 10 min. Peptide separation was carried out using a Thermo Fisher Scientific Vanquish Neo UHPLC system with an ES906 HPLC column (150 mm) and an 8-min gradient at 2.5 μl/min flow rate. The mobile phases were as follows: A (100% water, 0.1% formic acid) and B (80% acetonitrile). The gradient program was as follows: 0 to 4 min, linear increase from 4% to 25% B, 4 to 5.8 min, from 25% to 35% B, 5.8 to 6.2 min, from 35% to 99% B, 6.2 to 6.9 min, hold at 99% B. The separated peptides were analyzed using a Thermo Fisher Scientific Astral mass spectrometer operating in DIA mode, with normalized collision energy of 25%, default charge state of 2, resolution of 240,000, scan interval of 0.6 s, scan range from 380 to 980 mass/charge ratio (*m*/*z*), automatic gain control (AGC) setting of 500%, and fragmentation ion scans recorded with a maximum scan time of 3 ms. The DIA method used 300 scanning windows with 2-Th width across the 380 to 980 *m*/*z* range. For protein identification and quantification, the raw DIA data were analyzed using DIA-NN software in library-free mode. Mass spectrometry (MS)/MS data were searched against the UniProt protein sequence database with the following parameters: enzyme = Trypsin/P, maximum missed cleavages = 2, fixed modification = carbamidomethylation (C), variable modifications = oxidation (M) and acetylation (protein N-term), precursor mass tolerance = 20 ppm, fragment mass tolerance = 0.05 Da. Identifications were filtered at 1% false discovery rate (FDR), and only protein groups passing this threshold were used for downstream analysis.

### Cell culture supernatant Luminex assay

Supernatants of GES-1 or AGS cells cocultured with PMN-MDSCs were collected. Then, 37 chemokines were quantified using a magnetic Luminex Assay (R&D Systems) per the supplier’s protocol.

### ECAR and OCR measurement

After treatment, CD8^+^ T cells were resuspended in XF medium (nonbuffered RPMI 1640 comprising 10 mM glucose, 2 mM l-glutamine, and 1 mM sodium pyruvate) at a density of 1.5 × 10^5^ cells per well. Subsequently, ECAR and OCR were measured using an XFe96 Extracellular Flux Analyzer (Agilent Technologies, Santa Clara, CA). For ECAR measurement, sequential additions of metabolic modulators were performed: 10 mM glucose, 1 μM oligomycin, and 50 mM 2-deoxyglucose. For OCR measurement, the following metabolic modulators were added sequentially: 1 μM oligomycin, 1 μM carbonyl cyanide 4-(trifluoromethoxy) phenylhydrazone (FCCP), and 50 mM rotenone/antimycin A (ROT/AA). ECAR and OCR data were collected and analyzed using Wave software (Agilent Technologies), with values normalized to cell count.

### Acquisition and analysis of scRNA-seq data of stomach carcinogenesis

In total, 44 samples, including 7 CNAG, 3 CAG, 9 IM, and 25 intestinal-type GC samples, were analyzed in this study (Table S3). Four scRNA-seq datasets were procured, including 3 from the Gene Expression Omnibus (GEO) database (accession numbers GSE134520, GSE183904, and GSE150290) and 1 dataset from the Database of Genotypes and Phenotypes (dbGaP) (accession number phs001818.v2). Normalization was performed using Seurat’s NormalizeData function and followed by Harmony algorithm integration for batch effect correction. Principal components analysis (PCA) was conducted on the top 3,000 variable genes, with the top 20 principal components selected and a resolution parameter of 0.1 applied for dimensionality reduction. Reduced dimensions were projected via RunUMAP. Cluster-specific differentially expressed genes were identified using FindAllMarkers, annotating cell types through established marker genes (Table S4). The AddModuleScore function was applied to compute the MDSCs score and immunosuppressive score of myeloid cells, based on the reference gene set [26] (Tables S5 and S6).

### Acquisition and analysis of bulk RNA-seq data of stomach carcinogenesis

Gene expression profiles with clinical metadata from GEO datasets (GSE62254, GSE15459, GSE34942, GSE57303) were integrated into a meta-GEO cohort (295 intestinal-type GC/100 normal controls). Complementary TCGA data (359 GC samples) and GTEx controls (165 normals) established the TCGA-GTEx cohort, with batch effects corrected via “combat” algorithm (“sva” R package). Stomach carcinogenesis datasets were also downloaded, including GSE55696 (19 chronic gastritis, 19 LGIN, 20 HGIN, 19 EGC) and GSE191275 (10 chronic gastritis, 10 IM, 10 GC). MDSCs abundance was assessed via ssGSEA utilizing a specified gene signature [107] (Table S7) with the “GSVA” package in R.

### Pathway enrichment analysis

Functional annotation was executed via REACTOME pathway enrichment. The statistical significance of pathway enrichment was evaluated using the clusterProfiler (with adjusted *P* < 0.05 as threshold).

### Statistical analysis

All analyses employed GraphPad Prism (v.8.4.3, https://www.graphpad.com/) and R (v4.0.3). Student’s *t* test or the Mann–Whitney test was applied to assess differences between 2 groups, based on data distribution patterns. For multiple group comparisons, 1-way analysis of variance (ANOVA) or 2-way ANOVA with Bonferroni post hoc test was conducted for normally distributed data, while the Kruskal–Wallis test with Dunn post hoc test was applied for non-normally distributed data. Pearson’s or Spearman’s correlation analyses were executed to assess statistical correlations based on data distribution characteristics. Survival analysis was conducted using the log-rank test. ROC curve analysis was conducted to assess the diagnostic performance of the selected biomarkers. Significance was established at *P* < 0.05 (*P < 0.05, **P < 0.01, ***P < 0.005, ****P < 0.0001, and ns for nonsignificant) with data denoted as mean ± SEM.

## Ethical Approval

The study was conducted following the Declaration of Helsinki (as revised in 2013), and written consent for clinical specimens’ donation was obtained from each patient. The protocol was approved by the Institutional Review Board of First Affiliated Hospital of Guangzhou University of Chinese Medicine (approval nos. K-2025-108, K-2024-041, and K-2023-086). The protocols were approved by the Animal Research Ethics Board of Guangzhou University of Chinese Medicine (approval no. 20240326004, approved 2024 March 26) following the Guidelines for the Care and Use of Animals. The study involving animals was conducted following the Basel Declaration.

## Data Availability

Data and materials are available from the corresponding author on reasonable request.
