# Supplementary material for: CCL26–CX3CR1 Axis Mediates a Feedback Loop between Cancer Cell and PMN-MDSCs to Promote CD8+ T Cell Exhaustion during Stomach Carcinogenesis
Source: Research (Wash D C). 2025 Nov 21;8:1002. doi: 10.34133/research.1002 (PMC12635414; doi:10.34133/research.1002)
Supplement: Supplementary 1 — Tables S1 to S8 Figs. S1 to S19 [file research.1002.f1.docx]

**Title: CCL26-CX3CR1 axis mediates a feedback loop between cancer cell and PMN-MDSCs to promote CD8^+^ T-cell exhaustion during stomach carcinogenesis**

**Running title: CCL26-CX3CR1 axis: Feedback loop in GC cell and PMN-MDSCs interaction**

**Xiaotao Jiang^1,2#^, Hui Wu^1,2#^, Ning Yan^1,2#^, Anzhou Wu^1,2^, Xianzhe Wang^1,2^, Yi Wen^3^**, **Jinqi An^1,2^, Jingming Chen^1,2^, Jiaxing Yan^1,2^, Changlong Wang^1,2^, Yushan Zou^3^, Yuancheng Huang^4^, Wei Wang^2,5^*, Peiwu Li^9,^*, Kunhai Zhuang^3,6^*, Yufeng Liu^8,^*, Fengbin Liu^6,7^***

^1^ First Clinical Medical College, Guangzhou University of Chinese Medicine, Guangzhou, 510405, Guangdong, China

^2^ Lingnan Medical Research Center, Guangzhou University of Chinese Medicine, Guangzhou, 510405, Guangdong, China

^3^ The First Affiliated Hospital of Guangzhou University of Chinese Medicine, Guangzhou, 510405, Guangdong, China

^4^ Department of Oncology, Dongguan People’s Hospital (The Tenth Affiliated Hospital of Southern Medical University), Dongguan, 523000, Guangdong, China

^5^ Department of Gastrointestinal Surgery, The First Affiliated Hospital of Guangzhou University of Chinese Medicine, Guangzhou, 510405, Guangdong, China

^6^ Baiyun Hospital of The First Affiliated Hospital of Guangzhou University of Chinese Medicine, Guangzhou, 510470, Guangdong, China

^7^ Lingnan Institute of Spleen and Stomach Diseases, The First Affiliated Hospital of Guangzhou University of Chinese Medicine, Guangzhou, 510405, Guangdong, China

^8^ Center for medical research on innovation and translation, Guangzhou First People’s Hospital, Guangzhou, 510180, Guangdong, China.

^9^ Department of hepatobiliary diseases, The First Affiliated Hospital of Guangzhou University of Chinese Medicine, Guangzhou, 510405, Guangdong, China

*** Correspondence:**
Wei Wang, [wangwei164000@163.com](mailto:wangwei164000@163.com)

Peiwu Li, [doctorlipw2@163.com](mailto:doctorlipw2@163.com)

Kunhai Zhuang, sea-zhuang007@163.com

Yufeng Liu, eyyufengliu@scut.edu.cn

Fengbin Liu, liufengbin163@163.com

**Table S1. Summary of reagents and materials.**

| **Antibodies (Flow Cytometry)** | **Fluorophore** | **Company** | **IDENTIFIER** |
| --- | --- | --- | --- |
| **Human** |  |  |  |
| CD3 | PE-cy7 | TONBO | 60-0037 |
| CD4 | BUV496 | BD | 612936 |
| CD8 | PerCP-cy5.5 | TONBO | 65-0086 |
| CD19 | PE-Cy5 | BD | 555412 |
| CD14 | APC | BD | 561383 |
| CD15 | BV605 | Biolegend | 323031 |
| CD16 | BV786 | BD | 563690 |
| HLA-DR | APC-cy7 | TONBO | 259952 |
| CCR7 | AF700 | Biolegend | 353243 |
| CD45RA | BV510 | Biolegend | 304141 |
| CD127 | BV421 | Biolegend | 351309 |
| CD56 | PE594 | BD | 562289 |
| CD38 | PE | TONBO | 600388 |
| CD11b | BUV396 | BD | 563839 |
| CD25 | FITC | BD | 563840 |
| CD11c | BV711 | Biolegend | 301630 |
| CD3 | BV510 | BD | 563109 |
| CD8 | APC-cy7 | BD | 561945 |
| TNF-α | V450 | BD | 561311 |
| IFN-γ | APC | BD | 562017 |
| TGF-β1 | FITC | Biolegend | 300009 |
| TGF-β2 (in-direct staining) | / | Invitrogen | 710276 |
| TGF-β3 (in-direct staining) | / | Biorbyt | orb11470 |
| Goat Anti-Rabbit IgG H&L  (for in-direct staining) | PE | Abcam | ab72465 |
| PD-1 | FITC | Biolegend | 329904 |
| CX3CR1 | BV421 | Biolegend | 341620 |
| CCR3 | PE | Biolegend | 310706 |
| Rat IgG2b, κ Isotype Ctrl | BV421 | Biolegend | 400639 |
| Mouse IgG2b, κ Isotype Ctrl | PE | Biolegend | 401207 |
| **Mouse** |  |  |  |
| CD45 | BV510 | Biolegend | 103138 |
| CD11B | BV605 | Biolegend | 101257 |
| GR-1 | APC | Biolegend | 108412 |
| Ly-6G | APC-cy7 | Biolegend | 127624 |
| Ly-6C | FITC | Biolegend | 128006 |
| F4/80 | PE | Biolegend | 123110 |
| CD11c | PE594 | Biolegend | 117348 |
| MHC-II | BV421 | Biolegend | 107632 |
| CX3CR1 | AF700 | Biolegend | 149036 |
| IgG2a, κ Isotype Ctrl | AF700 | Biolegend | 400247 |
| 7-AAD | PerCP-cy5.5 | Biolegend | 420404 |
| CD3 | PE-Cy5 | Biolegend | 100274 |
| CD8 | BV650 | Biolegend | 100742 |
| TNF-α | PE | BD | 561063 |
| IFN-γ | APC | BD | 554413 |
| p-mTOR | FITC | Invitrogen | MA5-37146 |
| p-S6 | BV421 | Biolegend | 608609 |
| GLUT1 | PE | Novus | NB110-39113PE |
| Zombie | BV421 | Biolegend | 423113 |
| **Antibodies (Immunoblotting)** | **Company** | **IDENTIFIER** | |
| β-Actin Rabbit mAb | SAB | 52901-1 | |
| CCL26 Polyclonal Antibody | R&D Systems | bs-15513R | |
| Anti-SNAIL antibody | abcam | ab216347 | |
| SMAD2/3 Antibody | Cell signaling | 3102 | |
| Phospho-SMAD2 (Ser465/467)/SMAD3 (Ser423/425) Rabbit mAb | Cell signaling | 8828 | |
| **Antibodies (Immunofluorescence)** | **Company** | **IDENTIFIER** | |
| CD11b Antibody (ICRF44) | Thermo | 14-0118-82 | |
| CD15 Antibody | Immunoway | YT0726 | |
| CKR-3 Antibody | Immunoway | YT5289 | |
| Fractalkine Receptor Antibody | Immunoway | YT5112 | |
| Human CCL26/Eotaxin-3 Antibody | R&D Systems | AF653 | |
| TGF beta 1 Polyclonal Antibody | Bioss | BS-0086R | |
| **Antibodies (Neutralization)** | **Company** | **IDENTIFIER** | |
| Rabbit Anti-human CX3CR1 | Torrey Pines | TP502 | |
| Human CCR3 Antibody | R&D Systems | MAB155 | |
| Ultra-LEAF™ Purified anti-human/mouse TGF-β1 Antibody | Biolegend | 521707 | |
| Human IgG Control | R&D Systems | 1-001-A | |
| **Antibodies (ChIP Assay)** | **Company** | **IDENTIFIER** | |
| Snail (C15D3) Rabbit mAb | Cell signaling | 3879 | |
| Rabbit (DA1E) mAb IgG XP® Isotype Control | Cell signaling | 3900 | |
| **Recombinant protein** | **Company** | **IDENTIFIER** | |
| Recombinant Mouse TGF-beta 1 Protein | R&D Systems | 7666-MB | |
| Recombinant Human Eotaxin-3 (CCL26) (also interacting with Mouse) | PeproTech | 300-48 | |
| **Primers** | **Company** | **IDENTIFIER** | |
| Human β-actin forward primer  5’-CACCATTGGCAATGAGCGGTTC-3’ | Tsingke Biotechnology | customized | |
| Human β-actin reverse primer  5’-AGGTCTTTGCGGATGTCCACGT-3’ | Tsingke Biotechnology | customized | |
| Human CCL26 forward primer  5’-ATACAGCCACAAGCCCCTTC-3’ | Tsingke Biotechnology | customized | |
| Human CCL26 reverse primer  5’-TGGTAGTGAATATCACAGCCCG-3’ | Tsingke Biotechnology | customized | |
| Human SNAI1 forward primer  5’-TCGGAAGCCTAACTACAGCGA-3’ | Tsingke Biotechnology | customized | |
| Human SNAI1 reverse primer  5’-AGATGAGCATTGGCAGCGAG-3’ | Tsingke Biotechnology | customized | |
| Mouse *β-actin* forward primer  5’-CATTGCTGACAGGATGCAGAAGG-3’ | Tsingke Biotechnology | customized | |
| Mouse *β-actin* reverse primer  5’-TGCTGGAAGGTGGACAGTGAGG-3’ | Tsingke Biotechnology | customized | |
| Mouse *Tgfb1* forward primer  5’-GTCCAAACTAAGGCTCGCCA-3’ | Tsingke Biotechnology | customized | |
| Mouse *Tgfb1* reverse primer  5’-ATAGATGGCGTTGTTGCGGT-3’ | Tsingke Biotechnology | customized | |
| Mouse *Tgfb2* forward primer  5’-TTGTTGCCCTCCTACAGACTGG-3’ | Tsingke Biotechnology | customized | |
| Mouse *Tgfb2* reverse primer  5’-GTAAAGAGGGCGAAGGCAGCAA-3’ | Tsingke Biotechnology | customized | |
| Mouse *Tgfb3* forward primer  5’-AAGCAGCGCTACATAGGTGGCA-3’ | Tsingke Biotechnology | customized | |
| Mouse *Tgfb3* reverse primer  5’-GGCTGAAAGGTGTGACATGGAC-3’ | Tsingke Biotechnology | customized | |
| Mouse *Cx3cr1* forward primer  5’-GAGCATCACTGACATCTACCTCC-3’ | Tsingke Biotechnology | customized | |
| Mouse *Cx3cr1* reverse primer  5’-AGAAGGCAGTCGTGAGCTTGCA-3’ | Tsingke Biotechnology | customized | |
| Mouse *Ccl26* forward primer  5’-TCGCTATGTCCTGCTGCCCTAA-3’ | Tsingke Biotechnology | customized | |
| Mouse *Ccl26* reverse primer  5’-CTGGACACAGAATTGCTTACCTG-3’ | Tsingke Biotechnology | customized | |
| Mouse *Snai1* forward primer  5’-TGTCTGCACGACCTGTGGAAAG-3’ | Tsingke Biotechnology | customized | |
| Mouse *Snai1* reverse primer  5’-CTTCACATCCGAGTGGGTTTGG-3’ | Tsingke Biotechnology | customized | |
| **shRNAs** | **Company** | **IDENTIFIER** | |
| Human sh1-CCL26  5’-GCTGTGATATTCACTACCAAA-3’ | Focus Bioscience | customized | |
| Human sh2-CCL26  5’-CTGCTTCCAATACAGCCACAA-3’ | Focus Bioscience | customized | |
| Mouse sh1-SNAI1  5’-GCCACCTTCTTTGAGGTACAA-3’ | Focus Bioscience | customized | |
| Mouse sh2-SNAI1  5’-CCACTCGGATGTGAAGAGATA-3’ | Focus Bioscience | customized | |
| **activator or inhibitors** | **Company** | **IDENTIFIER** | |
| ITD-1 (Smad2/3 inhibitor) | Selleck | S6713 | |
| LY2109761 (TGFβRI/II inhibitor) | Selleck | S2704 | |
| MHY1485 (mTORC1 activator) | Selleck | S7811 | |

**Table S2. Clinical characteristics of patients.**

|  | **N** | **Age (±SEM)** | **Gender (F/M)** | **OLGA (I/II/III/IV)** | **OLGIM (I/II/III/IV)** |
| --- | --- | --- | --- | --- | --- |
| CNAG | 10 | 55.90(1.85) | 6/4 | / | / |
| CAG | 13 | 58.31(2.41) | 8/5 | 4/3/4/2 | 5/4/3/1 |
| GC | 11 | 59.64(3.16) | 5/6 | / | / |

**Table S3. Information of scRNA-seq datasets related to gastritis-cancer transformation.**

| **Dataset** | **CNAG (n)** | **CAG (n)** | **IM (n)** | **Intestinal-type GC (n)** |
| --- | --- | --- | --- | --- |
| GSE134520 | 3 | 3 | 6 | 1 |
| GSE183904 | 1 | / | / | 14 |
| GSE150290 | 3 | / | 2 | 9 |
| phs001818.v2 | / | / | 1 | 1 |
| total | 7 | 3 | 9 | 25 |

**Table S4.** **The markers for cell lineages in stomach.**

| **Cell lineages** | **Marker genes** |
| --- | --- |
| Pit mucous cell | EPCAM, MUC5AC |
| Gland mucous cell | EPCAM, MUC6 |
| Cancer cell | EPCAM, CEACAM5, CEACAM6 |
| Endocrine | EPCAM, GAST, GHRL, SST |
| Endothelial cell | PECAM1, VWF, ENG, MCAM |
| Fibroblast | FAP, PDPN, COL1A2, DCN, PDGFRA |
| Myeloids | CD68, CD14, CXCR2, CSF3R, CMTM2 |
| Neutrophil | CSF3R, CXCR2, CMTM2, S100A8, S100A9 |
| Monocyte | CD14, FCGR3A |
| Mast cell | TPSAB1, TPSB2, CPA3 |
| T/NK | CD3D, CD3E, CD3G, NKG7, KLRD1, GNLY |
| Treg | CD3D, CD3E, CD3G, CD4, FOXP3, IL2RA, IKZF2 |
| NK | XCL1, FCGR3A, KLRD1, KLRF1, GNLY, NKG7 |
| CD4^+^T naïve | CD3D, CD3E, CD3G, CD4, TCF7, SELL, LEF1, CCR7 |
| CD8^+^T | CD3D, CD3E, CD3G, CD8A, CD8B |
| Plasma B cells | IGHG4, MZB1, CD79A |
| Follicular B cells | CD19, CD79A, MS4A1 |
| Pericyte | GJA4, RGS5 |

**Table S5. Reference gene set used for score calculation to differentiate MDSCs from other myeloid cell populations in scRNA-seq analysis.**

| **Type** | **Markers^[1]^** |
| --- | --- |
| MDSCs | IL1B, CCR1, CXCL2, GRINA, IER3 |

[1] Tsutsumi C, Ohuchida K, Katayama N, Yamada Y, Nakamura S, Okuda S, Otsubo Y, Iwamoto C, Torata N, Horioka K, Shindo K, Mizuuchi Y, Ikenaga N, Nakata K, Nagai E, Morisaki T, Oda Y, Nakamura M. Tumor-infiltrating monocytic myeloid-derived suppressor cells contribute to the development of an immunosuppressive tumor microenvironment in gastric cancer. Gastric Cancer. 2024 Mar;27(2):248-262. doi: 10.1007/s10120-023-01456-4. Epub 2024 Jan 13. PMID: 38217732.

**Table S6. Reference gene set used for the calculation of the immunosuppressive score in scRNA-seq analysis.**

| **Type** | **Markers** |
| --- | --- |
| Immunosuppressive score | ARG1, S100A8, S100A9, TGFB1, TGFB2, TGFB3, IL10, CD274, IDO1, NOS2 |

**Table S7. The markers of MDSCs used for ssGSEA in bulk RNA-seq analysis.**

| **Cell type** | **Markers^[1]^** |
| --- | --- |
| MDSCs | ARG1, CEACAM1, FKBP5, HMGB2, OLR1, LCN2, MMP8, MMP9 |

[1] Ghosh S, Huang J, Inkman M, Zhang J, Thotala S, Tikhonova E, Miheecheva N, Frenkel F, Ataullakhanov R, Wang X, DeNardo D, Hallahan D, Thotala D. Radiation-induced circulating myeloid-derived suppressor cells induce systemic lymphopenia after chemoradiotherapy in patients with glioblastoma. Sci Transl Med. 2023 Jan 25;15(680):eabn6758. doi: 10.1126/scitranslmed.abn6758. Epub 2023 Jan 25. PMID: 36696484; PMCID: PMC10501302.

**Table S8. Potential binding sites of SNAI1 in the promoter region of *Ccl26*.**

**
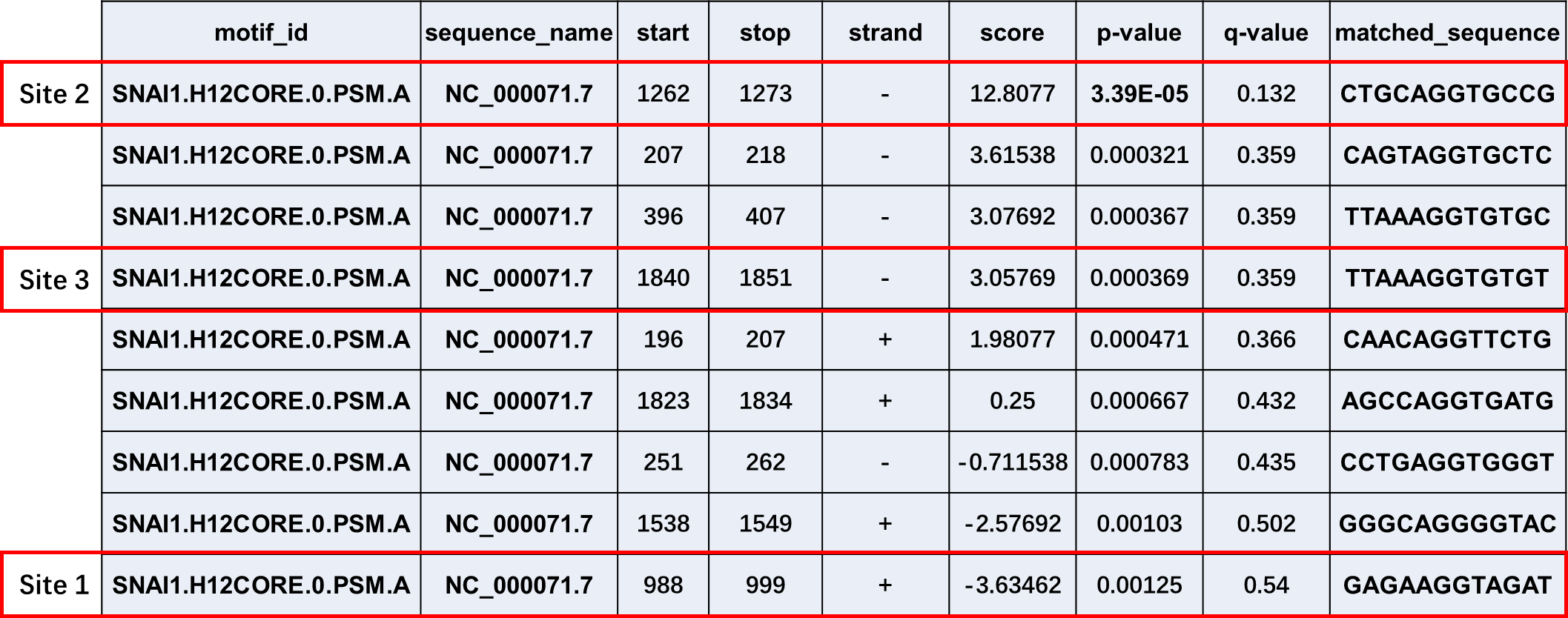
**

**Fig. S1**


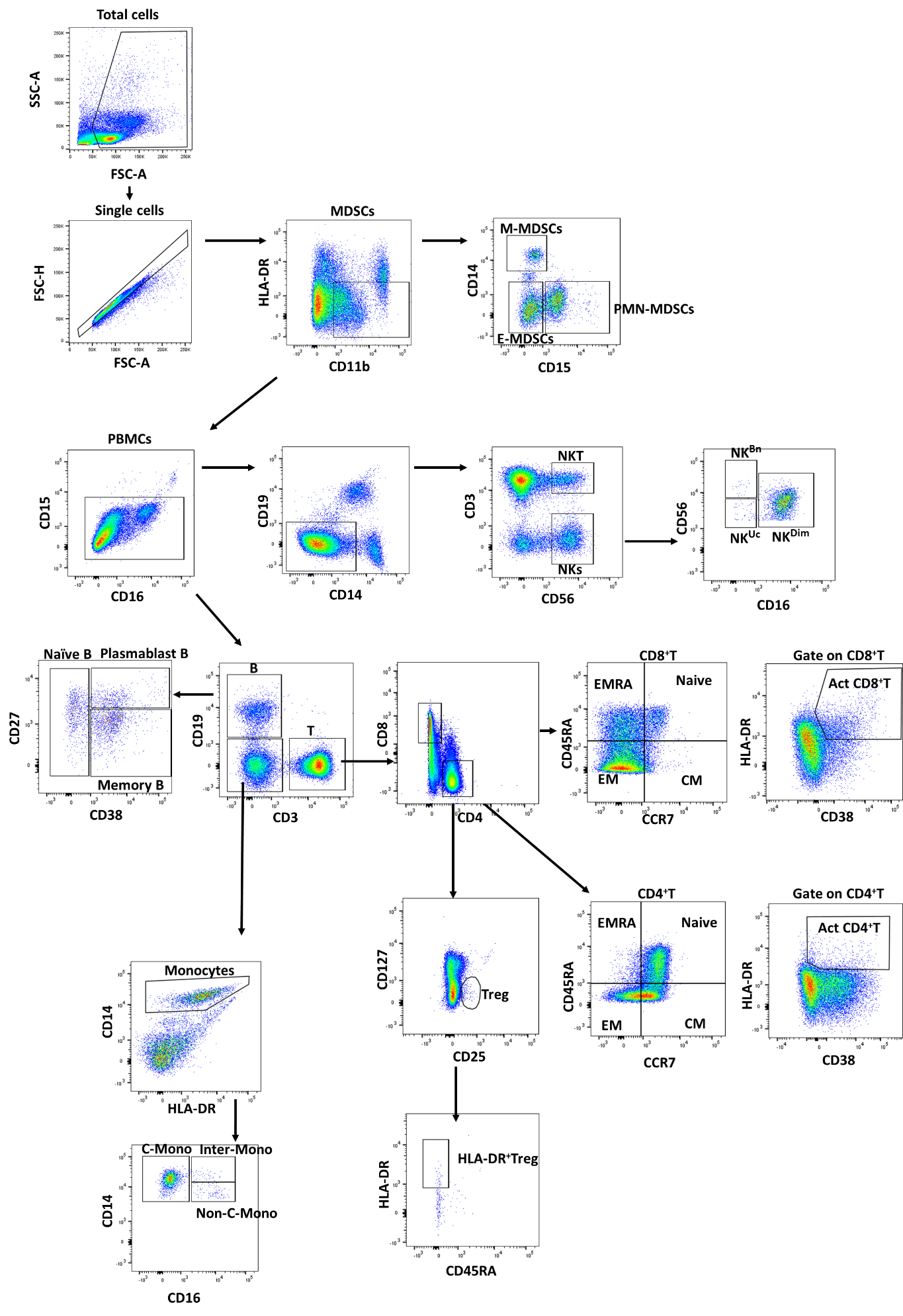


**Fig. S1: Gating strategy in peripheral human blood samples.**

**Fig. S2**

**
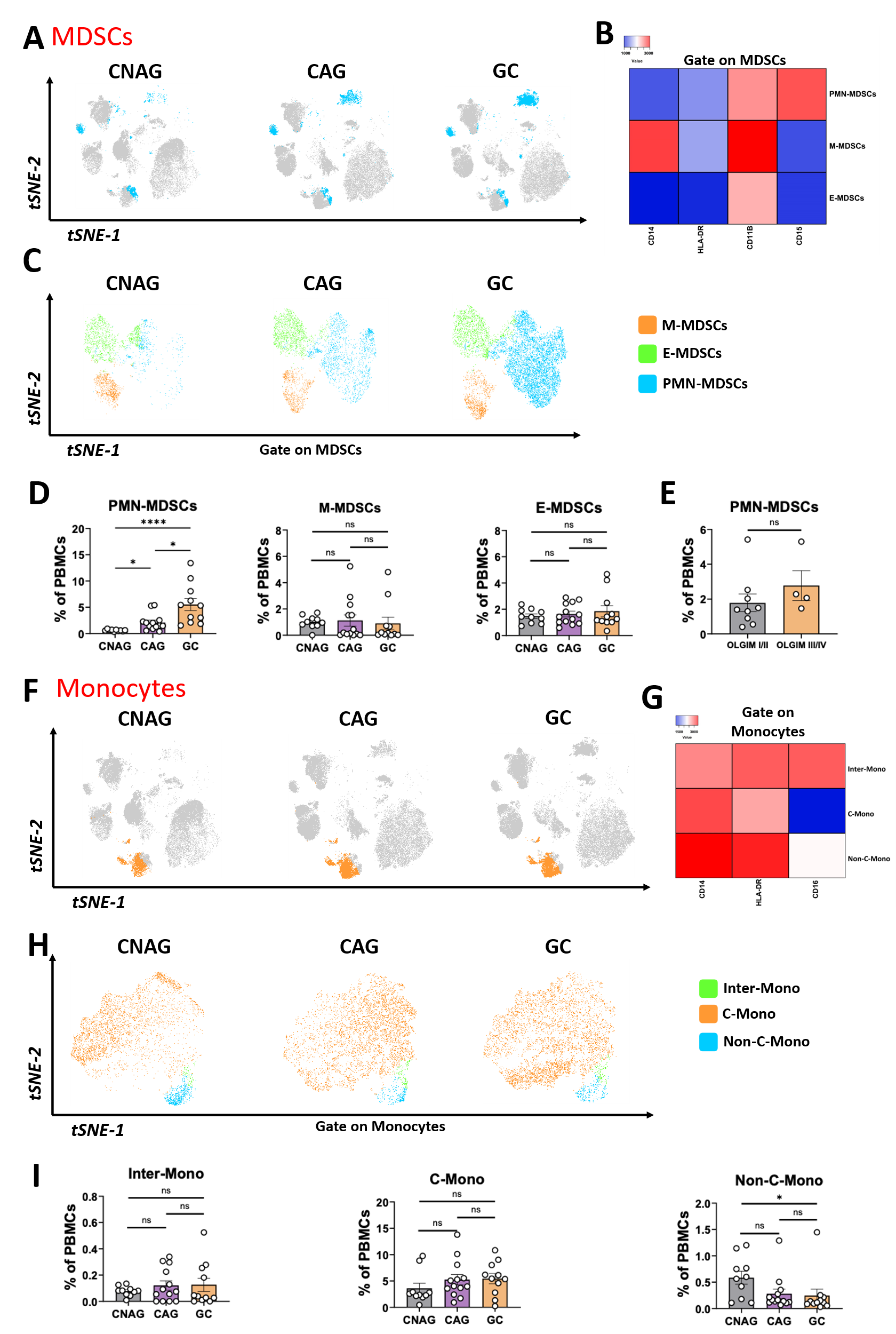
**

**Fig. S2: Dimensionality reduction analysis of MDSCs and Monocytes.**

A: T-SNE plot of MDSCs of PBMCs from CNAG, CAG and GC group.

B: Heatmap of major subsets in MDSCs, clustered by their relative expression of the markers.

C: T-SNE plot of MDSCs subsets gated on MDSCs. Cells are colored based on cell types.

D: Percentage of subset of MDSCs from CNAG, CAG and GC group.

E: Percentage of subset of MDSCs from OLGIM I/II and OLGIM III/IV in CAG group.

F: T-SNE plot of Monocytes of PBMCs from CNAG, CAG and GC group.

G: Heatmap of major subsets in Monocytes, clustered by their relative expression of the markers.

H: T-SNE plot of Monocytes subsets gated on MDSCs. Cells are colored based on cell types.

I: Percentage of subset of Monocytes from CNAG, CAG and GC group.

Data are presented as mean ± SEM for the bar chart. The sample sizes are as follows: CNAG (n = 10), CAG (n = 13), and GC (n = 11), with OLGIM I/II (n = 9) and OLGIM III/IV (n = 4). Data of (E) were compared using the Mann-Whitney test. Data of (D) and (I) were compared using the Kruskal-Wallis test followed by Dunnett's post hoc test. **P* < 0.05, ***P* < 0.01, ****P* < 0.005, *****P* < 0.0001, and ns for non-significant.

**Fig. S3**

**
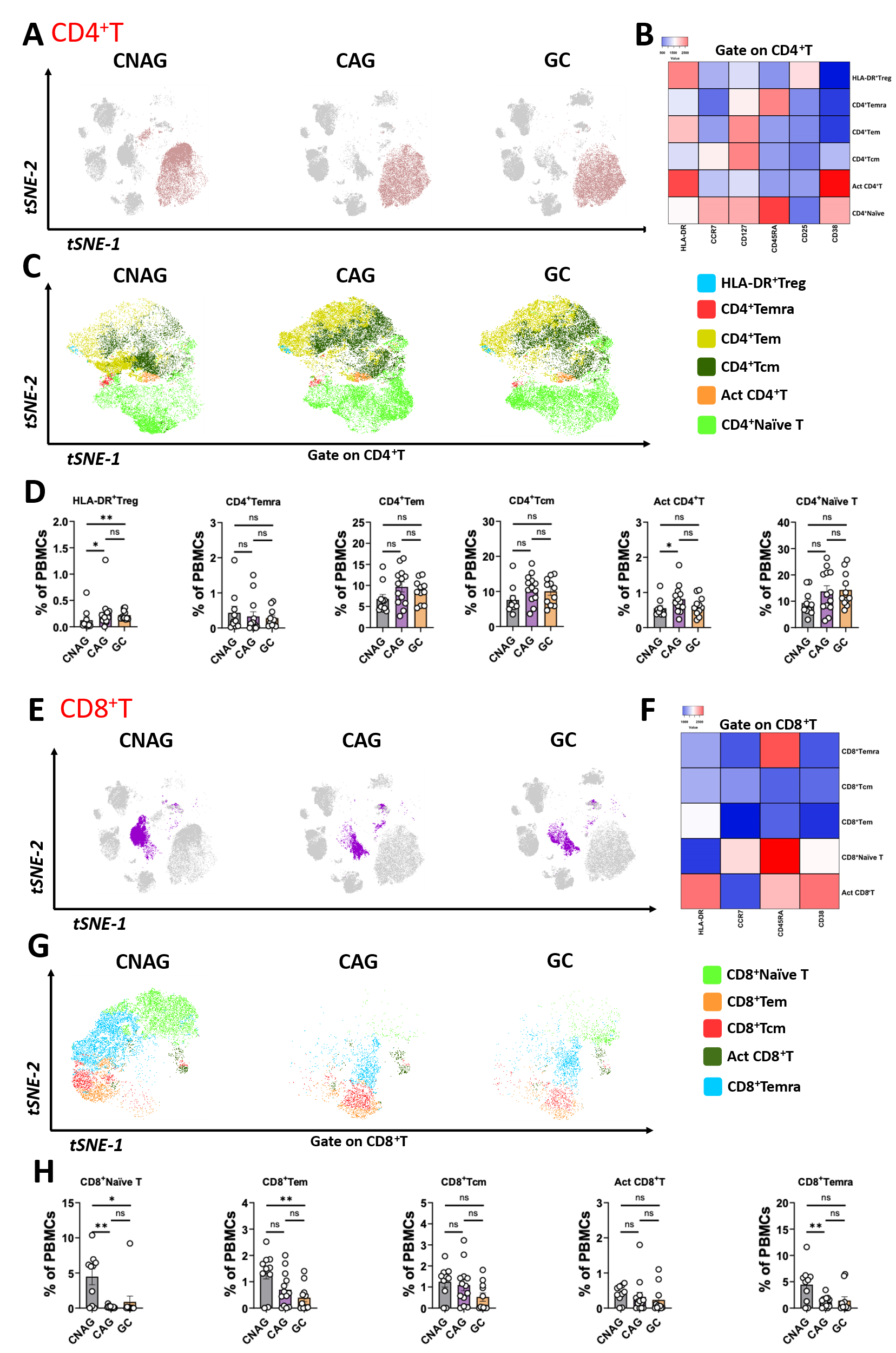
**

**Fig. S3: Dimensionality reduction analysis of** **CD4^+^T cells and CD8^+^T cells.**

A: T-SNE plot of CD4^+^T cells of PBMCs from CNAG, CAG and GC group.

B: Heatmap of major subsets in CD4^+^T cells, clustered by their relative expression of the markers.

C: T-SNE plot of CD4^+^T cells subsets gated on CD4^+^T cells. Cells are colored based on cell types.

D: Percentage of subset of CD4^+^T cells from CNAG, CAG and GC group.

E: T-SNE plot of CD8^+^T cells of PBMCs from CNAG, CAG and GC group.

F: Heatmap of major subsets in CD8^+^T cells, clustered by their relative expression of the markers.

G: T-SNE plot of CD8^+^T cells subsets gated on CD8^+^T cells. Cells are colored based on cell types.

H: Percentage of subset of CD8^+^T cells from CNAG, CAG and GC group.

Data are presented as mean ± SEM for the bar chart. Data of (D) and (H) were compared using the Kruskal-Wallis test followed by Dunnett's post hoc test. **P* < 0.05, ***P* < 0.01, ****P* < 0.005, *****P* < 0.0001, and ns for non-significant.

**Fig. S4**

**
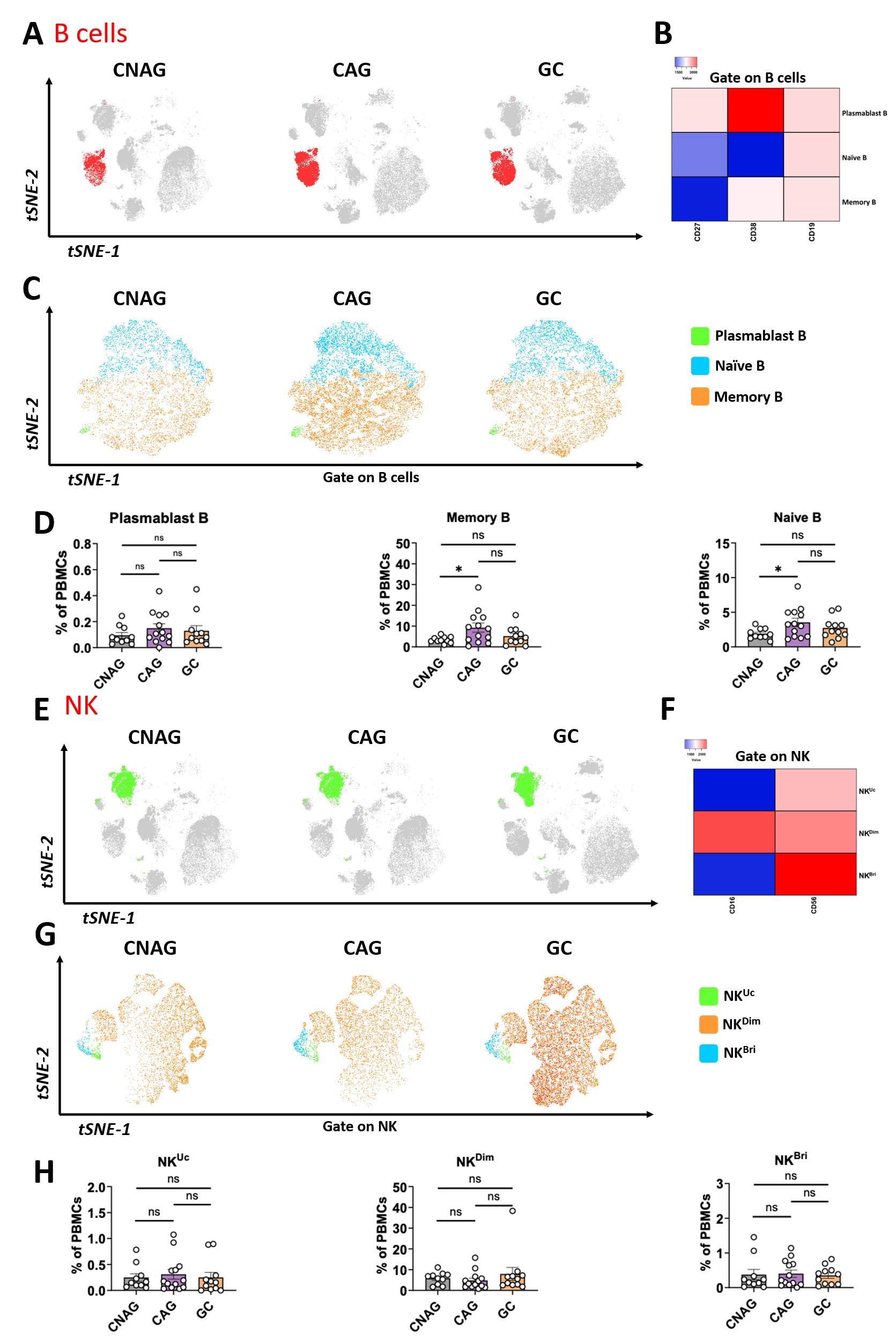
**

**Fig. S4: Dimensionality reduction analysis of B cells and NK cells.**

A: T-SNE plot of B cells of PBMCs from CNAG, CAG and GC group.

B: Heatmap of major subsets in B cells, clustered by their relative expression of the markers.

C: T-SNE plot of B cells subsets gated on B cells. Cells are colored based on cell types.

D: Percentage of subset of B cells from CNAG, CAG and GC group.

E: T-SNE plot of NK cells of PBMCs from CNAG, CAG and GC group.

F: Heatmap of major subsets in NK cells, clustered by their relative expression of the markers.

G: T-SNE plot of NK cells subsets gated on NK cells. Cells are colored based on cell types.

H: Percentage of subset of NK cells from CNAG, CAG and GC group.

Data of (D) and (H) were compared using the Kruskal-Wallis test followed by Dunnett's post hoc test. **P* < 0.05, ***P* < 0.01, ****P* < 0.005, *****P* < 0.0001, and ns for non-significant.

**Fig. S5**

**
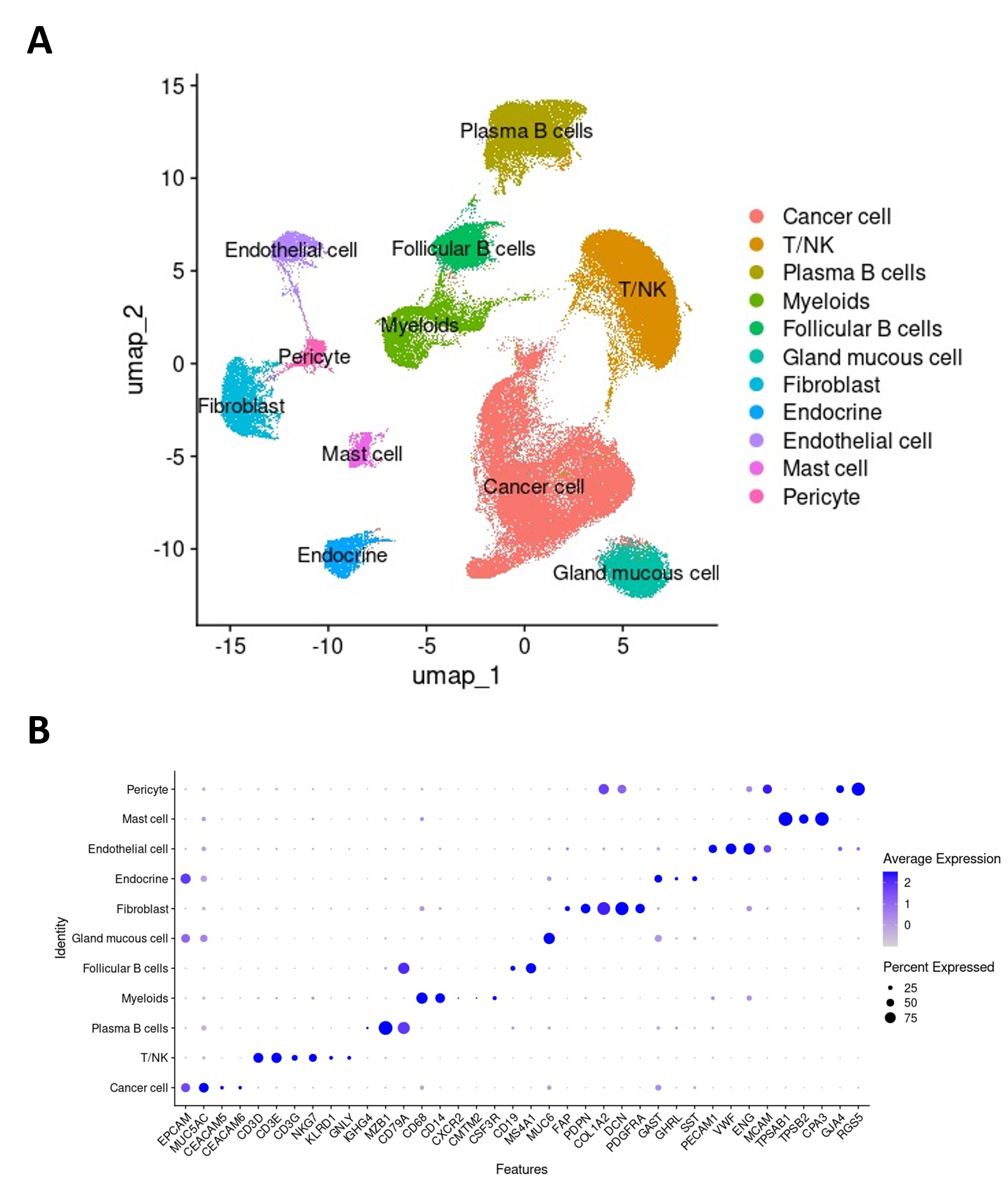
**

**Fig. S5: Analysis of scRNA-seq data of stomach carcinogenesis.**

A: UMAP plot showing the distribution of different cell types during stomach carcinogenesis.

B: Dot plot of cell types markers across different cell types.

Data were derived from four scRNA-seq datasets (GEO: GSE134520, GSE183904, GSE150290; dbGaP: phs001818.v2), comprising a total of n = 44 patients. Processing was conducted in Seurat (v4.3.0), with quality control (200-6,000 genes per cell; mitochondrial RNA ≤ 10%), LogNormalize normalization, and selection of 3,000 highly variable genes, followed by PCA using the first 20 components. Datasets were integrated using Harmony for batch correction. Clustering (resolution r = 0.1) and UMAP were performed on the Harmony-corrected principal components, using dimensions 1-20.

**Fig. S6**

**
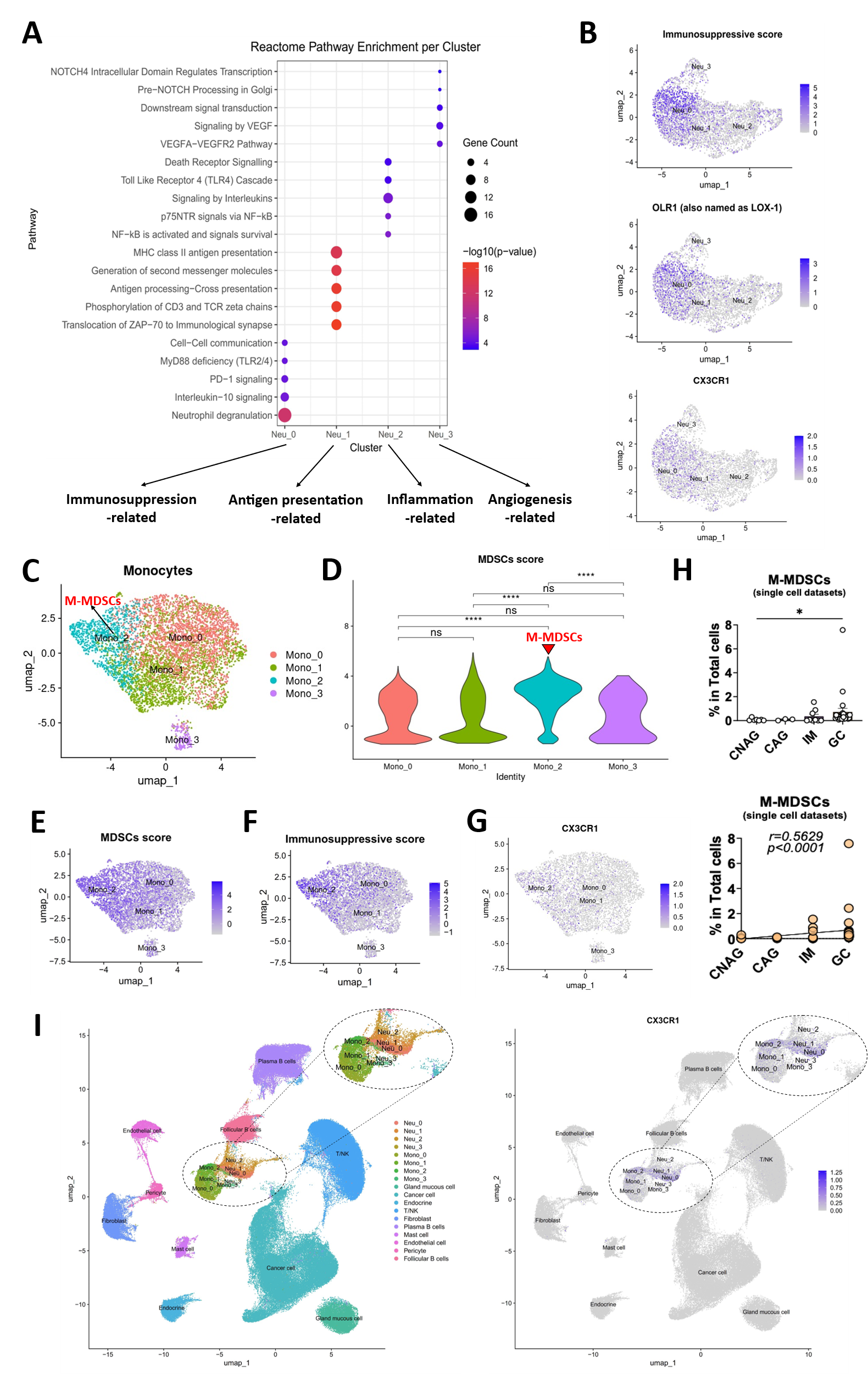
**

**Fig. S6:** **Analysis of scRNA‐seq data of stomach carcinogenesis.**

A. Functional enrichment analysis of the four neutrophil subsets.
B. UMAP plots showing the distribution of immunosuppressive scores, OLR1 (also known as LOX-1), and CX3CR1 expression in neutrophils.
C. UMAP plot of the four monocyte subsets.
D. Violin plot showing MDSC scores across the four monocyte subsets.
E. UMAP plots illustrating the distribution of MDSC scores across the four monocyte subsets.
F. UMAP plot showing the distribution of immunosuppressive scores and OLR1 expression across the four monocyte subsets.
G. UMAP plot depicting the distribution of CX3CR1 expression across the four monocyte subsets.
H. Quantification of relative M-MDSC percentages in scRNA-seq datasets across the CNAG (n = 7), CAG (n = 3), IM (n = 9), and GC (n = 25) groups. The correlation between M-MDSC percentage and pathological stage is shown below.
I. UMAP plot illustrating the distribution of different cell types during stomach carcinogenesis and CX3CR1 expression.

Data were derived from four scRNA-seq datasets (GEO: GSE134520, GSE183904, GSE150290; dbGaP: phs001818.v2), comprising a total of n = 44 patients. Processing was conducted in Seurat (v4.3.0), with quality control (200-6,000 genes per cell; mitochondrial RNA ≤ 10%), LogNormalize normalization, and selection of 3,000 highly variable genes, followed by PCA using the first 20 components. Datasets were integrated using Harmony for batch correction. Clustering (resolution r = 0.1) and UMAP were performed on the Harmony-corrected principal components, using dimensions 1-20.

**Fig. S7**

**
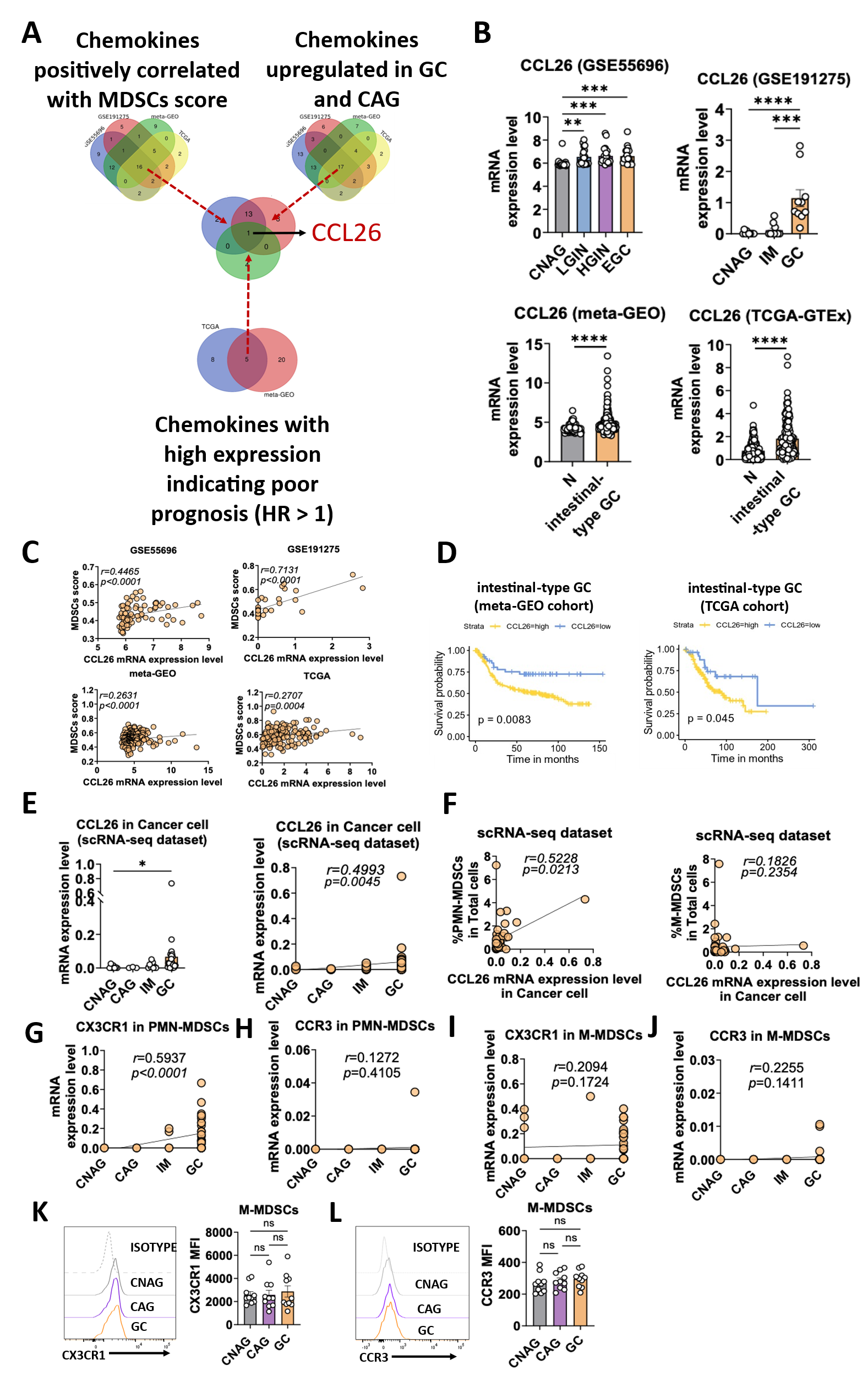
**

**Fig. S7:** **The correlation of CCL26 with pathological stages, prognosis, and MDSCs, along with the expression analysis of CX3CR1 and CCR3 in PMN-MDSCs and M-MDSCs.**

A: Venn diagram identifying CCL26 as a key factor in stomach carcinogenesis and MDSCs chemotaxis.

B: The expression levels of CCL26 in GSE55696, GSE191275, meta-GEO, and TCGA datasets.

C: Scatter plot showing the correlation between CCL26 expression levels and MDSCs score.

D: Kaplan-Meier analysis showing the overall survival of CCL26 in patients with intestinal-type GC in meta-GEO, and TCGA datasets.

E: The expression of CCL26 in cancer cells during stomach carcinogenesis and its correlation with pathological stages in scRNA-seq data.

F: Correlation of the expression of CCL26 in cancer cell with the proportions of PMN-MDSCs and M-MDSCs in scRNA-seq data.

G: Correlation of the expression of CX3CR1 in PMN-MDSCs with pathological stages.

H: Correlation of the expression of CCR3 in PMN-MDSCs with pathological stages.

I: Correlation of the expression of CX3CR1 in M-MDSCs with pathological stages.

J: Correlation of the expression of CCR3 in M-MDSCs with pathological stages.

K: Flow cytometry analysis of CX3CR1 expression in M-MDSCs from patients with CNAG, CAG, and GC. Bar graph on the right quantifies the MFI of CX3CR1 across the three groups.

L: Flow cytometry analysis of CCR3 expression in M-MDSCs from patients with CNAG, CAG, and GC. Bar graph on the right quantifies the MFI of CCR3 across the three groups.

Data are presented as mean ± SEM for the bar charts. Sample sizes are as follows: CNAG (n = 10), IM (n = 10), and GC (n = 10) for GSE191275; CNAG (n = 19), LGIN (n = 19), HGIN (n = 20), and EGC (n = 19) for GSE55696; meta-GEO (normal control, n = 133 vs. intestinal-type GC, n = 295); and TCGA-GTEx (normal control, n = 359 vs. intestinal-type GC, n = 165). Bar plots of (B) and (E) were compared using the Kruskal-Wallis test followed by Dunnett's post hoc test or the Mann-Whitney test. Correlation analysis of (C), (E), (F), (G), (H), (I), and (J) was performed using Spearman’s correlation. Data in (D) were analyzed using the log-rank test. Data in (K) and (L) were analyzed using one-way ANOVA followed by Bonferroni’s post hoc test. **P* < 0.05, ***P* < 0.01, ****P* < 0.005, *****P* < 0.0001, and ns for non-significant.

**Fig. S8**


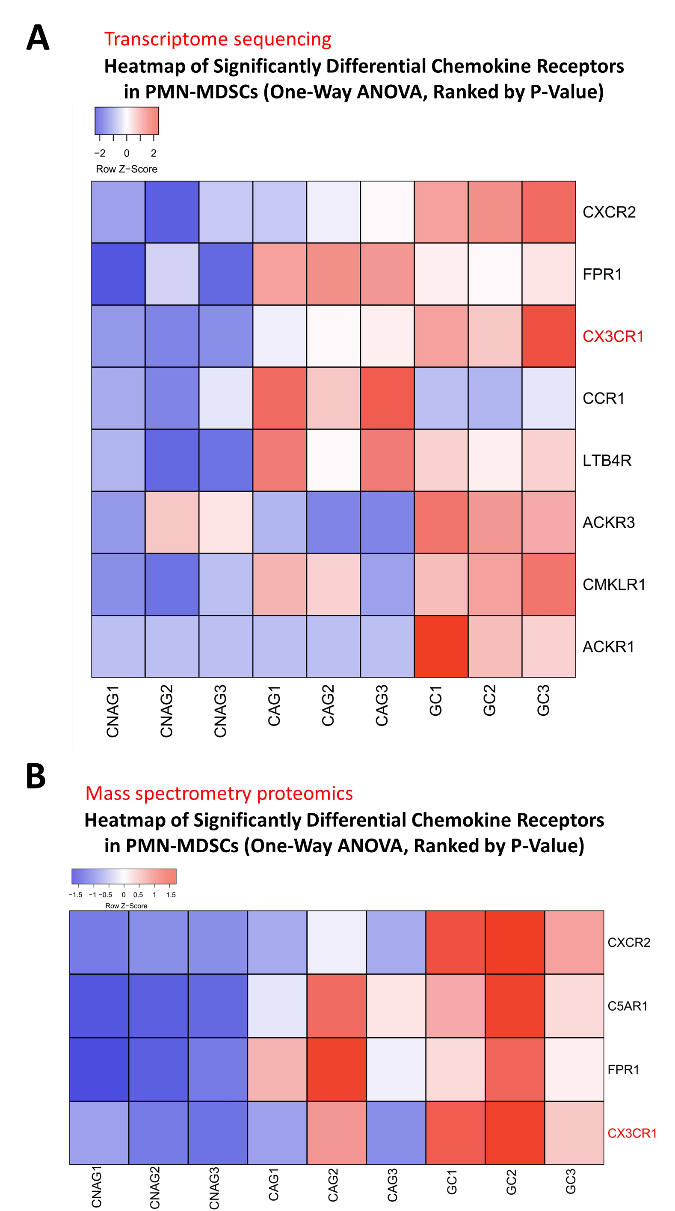


**Fig. S8:** **Transcriptomic and Proteomic analysis of differentially expressed chemokine receptors in PMN-MDSCs.**

A: Heatmap showing the relative expression of significantly differentially expressed chemokine receptors (CXCR2, FPR1, CX3CR1, CCR1, LTB4R, ACKR3, CMKLR1, and ACKR1) in PMN-MDSCs across different stages (CNAG, CAG, and GC) based on transcriptome sequencing data. Z-scores are represented with a color gradient, where blue indicates lower expression and red indicates higher expression.
B: Heatmap showing the relative protein expression of significantly differentially expressed chemokine receptors (CXCR2, C5AR1, FPR1, and CX3CR1) in PMN-MDSCs across different stages (CNAG, CAG, and GC) based on mass spectrometry proteomics data. The color gradient represents protein abundance, with blue indicating lower levels and red indicating higher levels.

**Fig. S9**

**
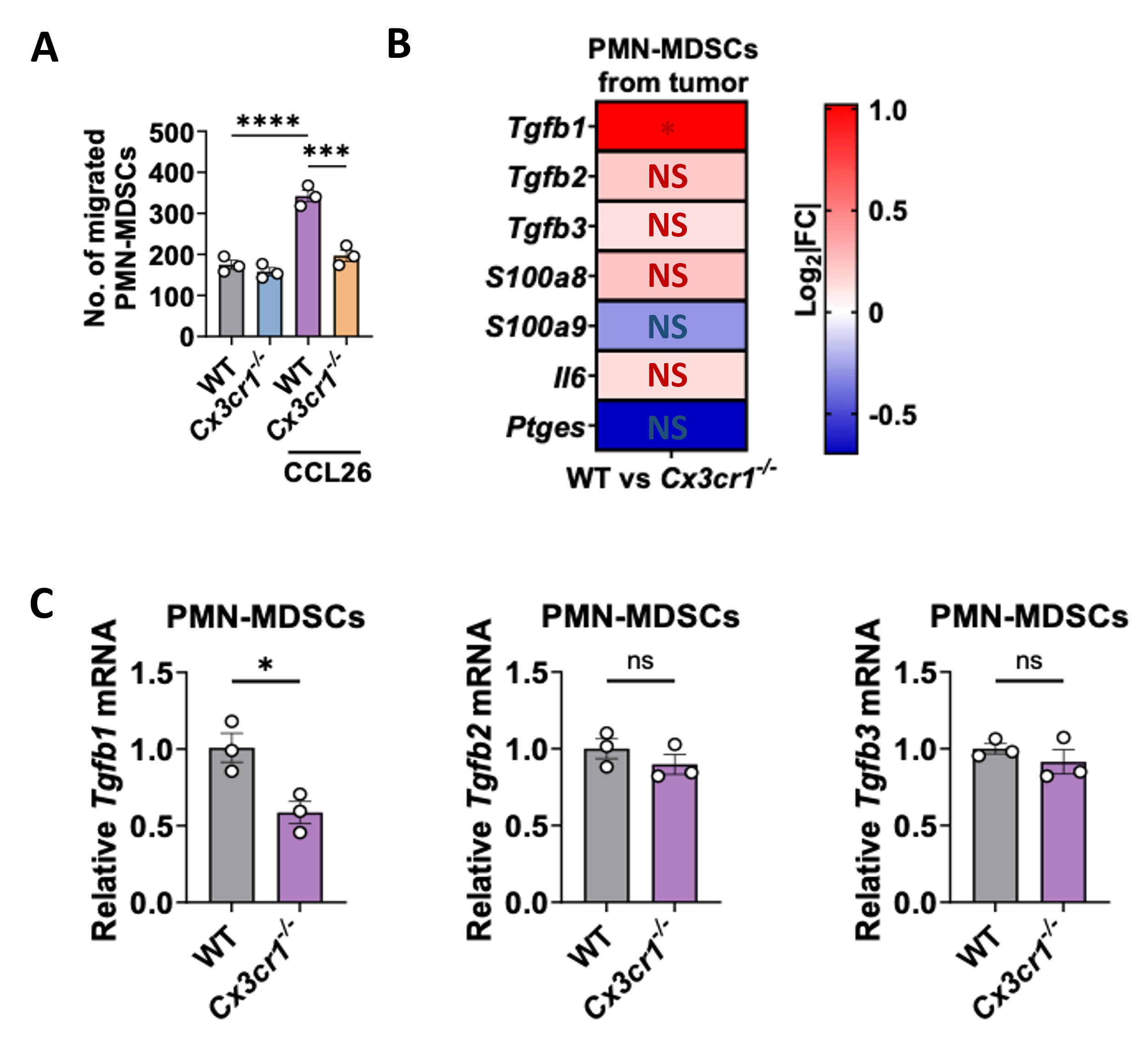
**

**Fig. S9: Migration assays and gene expression profiling of PMN-MDSCs from WT and *Cx3cr1^-/-^* mice.**

A: Number of migrated PMN-MDSCs from WT and *Cx3cr1^-/-^* mice in response to conditioned media with or without CCL26.

B. Heatmap showing the log2 fold change (Log2|FC|) and p-value of pro-tumorigenic and immunosuppressive factors, including *Tgfb1*, *Tgfb2*, *Tgfb3*, *S100a8*, *S100a9*, *Il6*, and *Ptges*. Gene expression comparisons were performed between WT and *Cx3cr1^-/-^* PMN-MDSCs from tumors.
C. Relative mRNA levels of *Tgfb1*, *Tgfb2*, and *Tgfb3* in WT and *Cx3cr1^-/-^* PMN-MDSCs from tumors.

Data are presented as mean ± SEM for the bar charts. All groups had n = 3 biological replicates. Data of (A) were analyzed using one-way ANOVA followed by Bonferroni’s post hoc test. Data of (C) were analyzed using Student’s t-test. **P* < 0.05, ***P* < 0.01, ****P* < 0.005, *****P* < 0.0001, and ns for non-significant.

**Fig. S10**

**
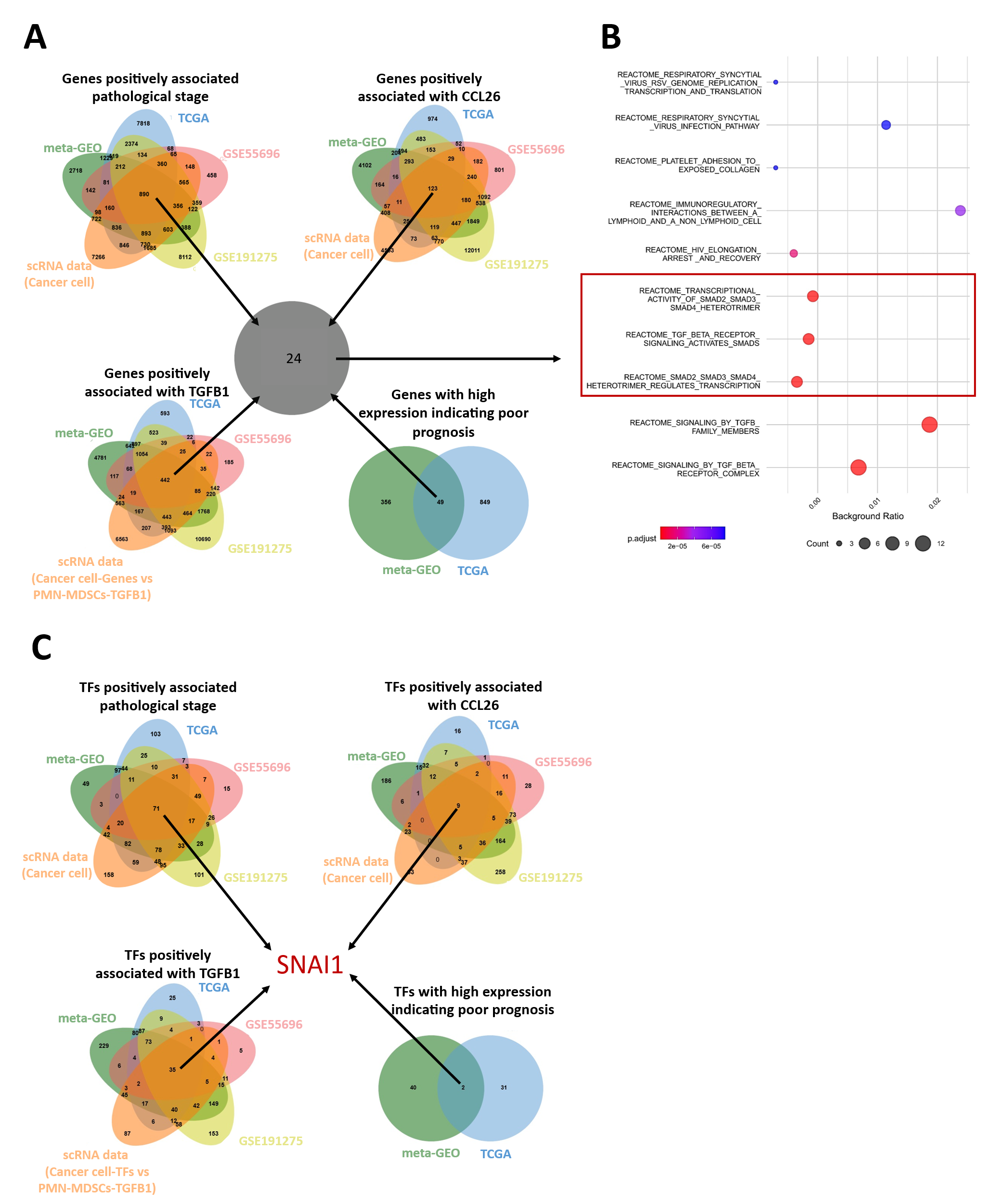
**

**Fig. S10: Key pathways and transcription factors associated with TGFB1 and CCL26.**

A: Venn diagram identifying 24 genes associated with stomach carcinogenesis, poor prognosis, TGFB1 and CCL26 expression levels.

B: Reactome enrichment analysis of 24 identified genes.

C: Venn diagram identifying SNAI1 associated with stomach carcinogenesis, poor prognosis, TGFB1 and CCL26 expression levels.

**Fig. S11**


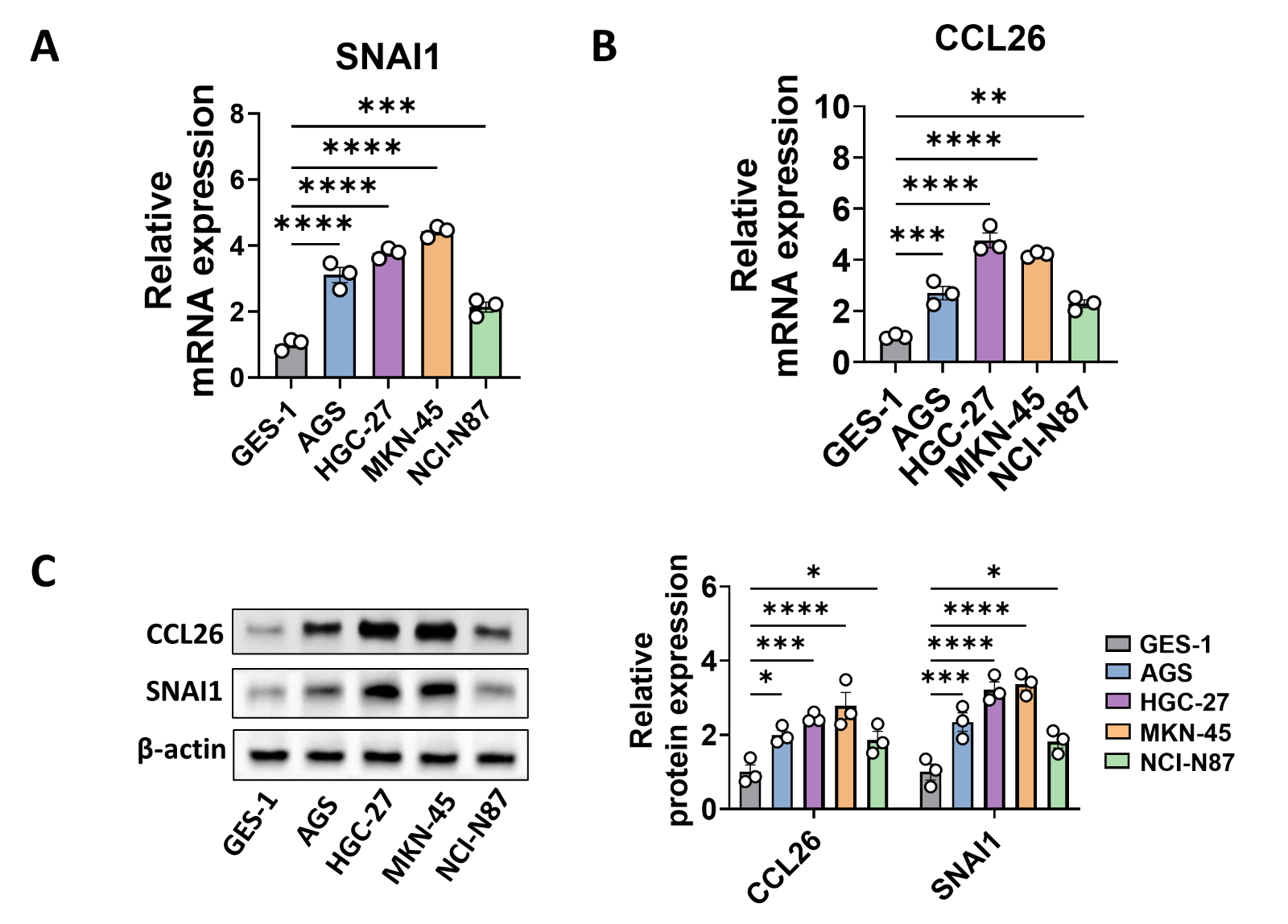


**Fig. S11: Comparative expression of SNAI1 and CCL26 at the mRNA and protein levels in normal and human malignant gastric epithelial cells.**

(A) Relative SNAI1 mRNA levels in various gastric cell lines.

(B) Relative CCL26 mRNA levels in various gastric cell lines.

(C) Western blot analysis of CCL26 and SNAI1 across various gastric cell lines.

Data are presented as mean ± SEM for the bar charts. All groups had n = 3 biological replicates. Data of (A) and (B) were analyzed using one-way ANOVA followed by Bonferroni’s post hoc test. Data of (C) were analyzed using two-way ANOVA followed by Bonferroni’s post hoc test. **P* < 0.05, ***P* < 0.01, ****P* < 0.005, *****P* < 0.0001, and ns for non-significant.

**Fig. S12**

**
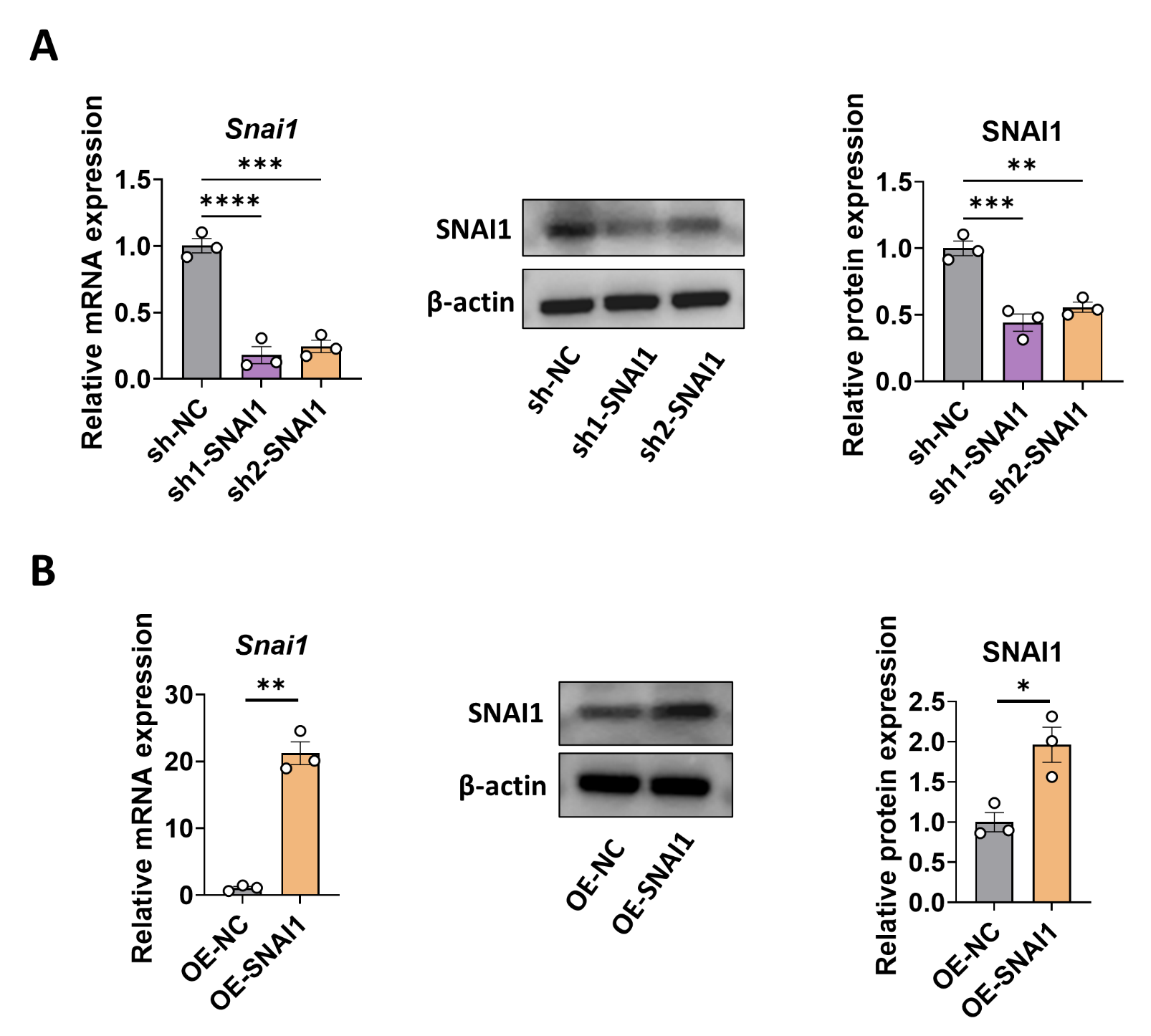
**

**Fig. S12: Confirmation of SNAI1 knockdown and overexpression in MFC cells.**

A: Validation of SNAI1 knockdown in MFC cells at both mRNA and protein levels.

B: Validation of SNAI1 overexpression in MFC cells at both mRNA and protein levels.

Data are presented as mean ± SEM for the bar charts. All groups had n = 3 biological replicates. Data of (A) were analyzed using one-way ANOVA followed by Bonferroni’s post hoc test. Data of (B) were analyzed using Student’s t-test. **P* < 0.05, ***P* < 0.01, ****P* < 0.005, *****P* < 0.0001, and ns for non-significant.

**Fig. S13**

**
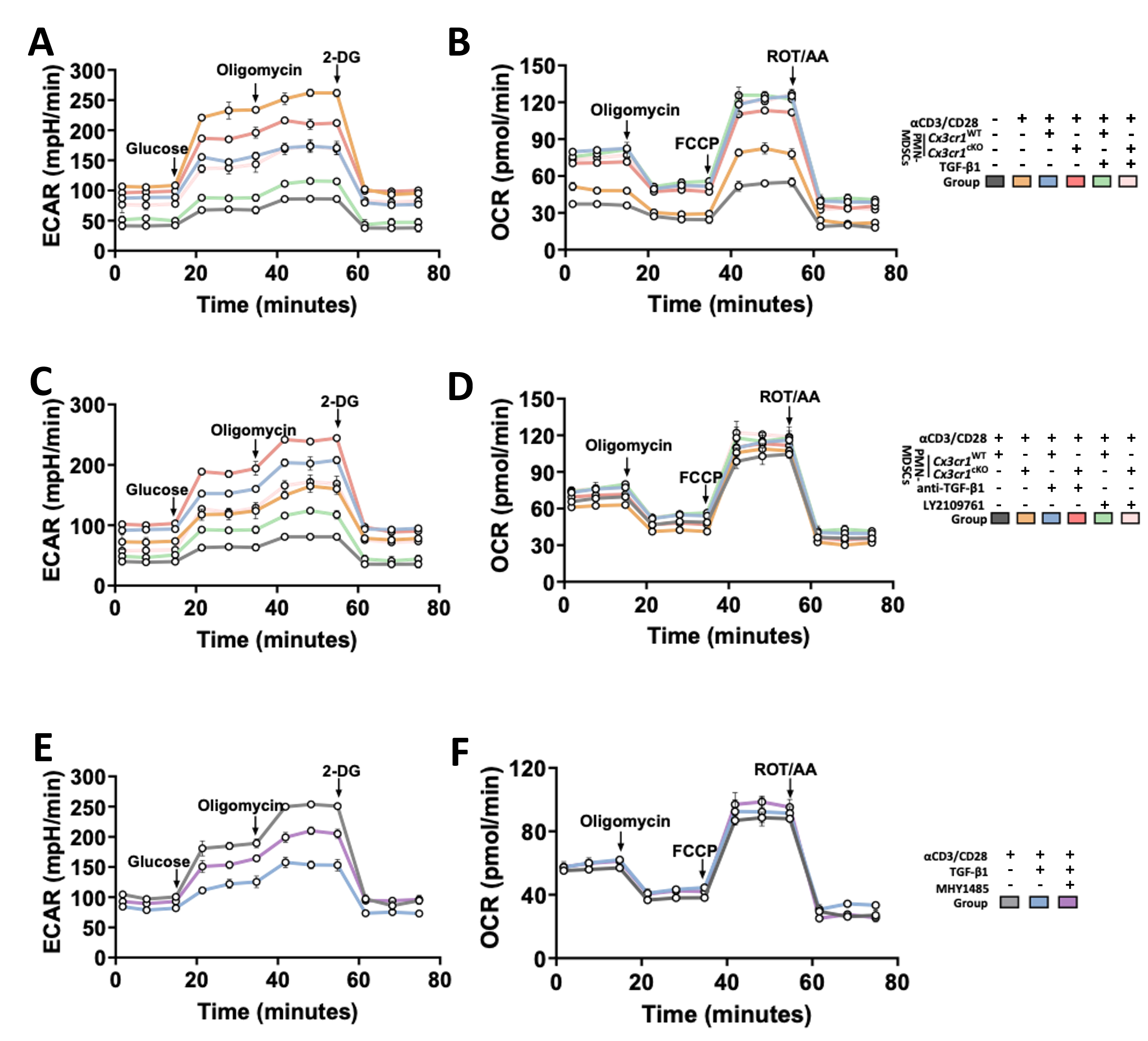
**

**Fig. S13: Metabolic profiling of CD8⁺ T cells under various stimulation and inhibitory conditions.**

A: Time-course measurement of ECAR in CD8⁺ T cells co-cultured with *Cx3cr1*^cKO^ and *Cx3cr1*^WT^ PMN-MDSCs, and treated with TGF-β1, following sequential stimulation with glucose, oligomycin, and 2-DG.

B: Time-course measurement of OCR in CD8⁺ T cells co-cultured with *Cx3cr1*^cKO^ and *Cx3cr1*^WT^ PMN-MDSCs, and treated with TGF-β1, following sequential stimulation with oligomycin, FCCP and ROT/AA.

C: Time-course measurement of ECAR in CD8⁺ T cells co-cultured with *Cx3cr1*^cKO^ and *Cx3cr1*^WT^ PMN-MDSCs, and treated with either anti-TGF-β1 or the TGFβRI/II inhibitor LY2109761, following sequential stimulation with glucose, oligomycin, and 2-DG.

D: Time-course measurement of OCR in CD8⁺ T cells co-cultured with *Cx3cr1*^cKO^ and *Cx3cr1*^WT^ PMN-MDSCs, and treated with either anti-TGF-β1 or the TGFβRI/II inhibitor LY2109761, following sequential stimulation with oligomycin, FCCP and ROT/AA.

E: Time-course measurement of ECAR in CD8⁺ T cells treated with TGF-β1 alone or TGF-β1 in combination with the mTOR activator, MHY1485, following sequential stimulation with glucose, oligomycin, and 2-DG.

F: Time-course measurement of OCR in CD8⁺ T cells treated with TGF-β1 alone or TGF-β1 in combination with the mTOR activator, MHY1485, following sequential stimulation with oligomycin, FCCP and ROT/AA.

**Fig. S14**

**
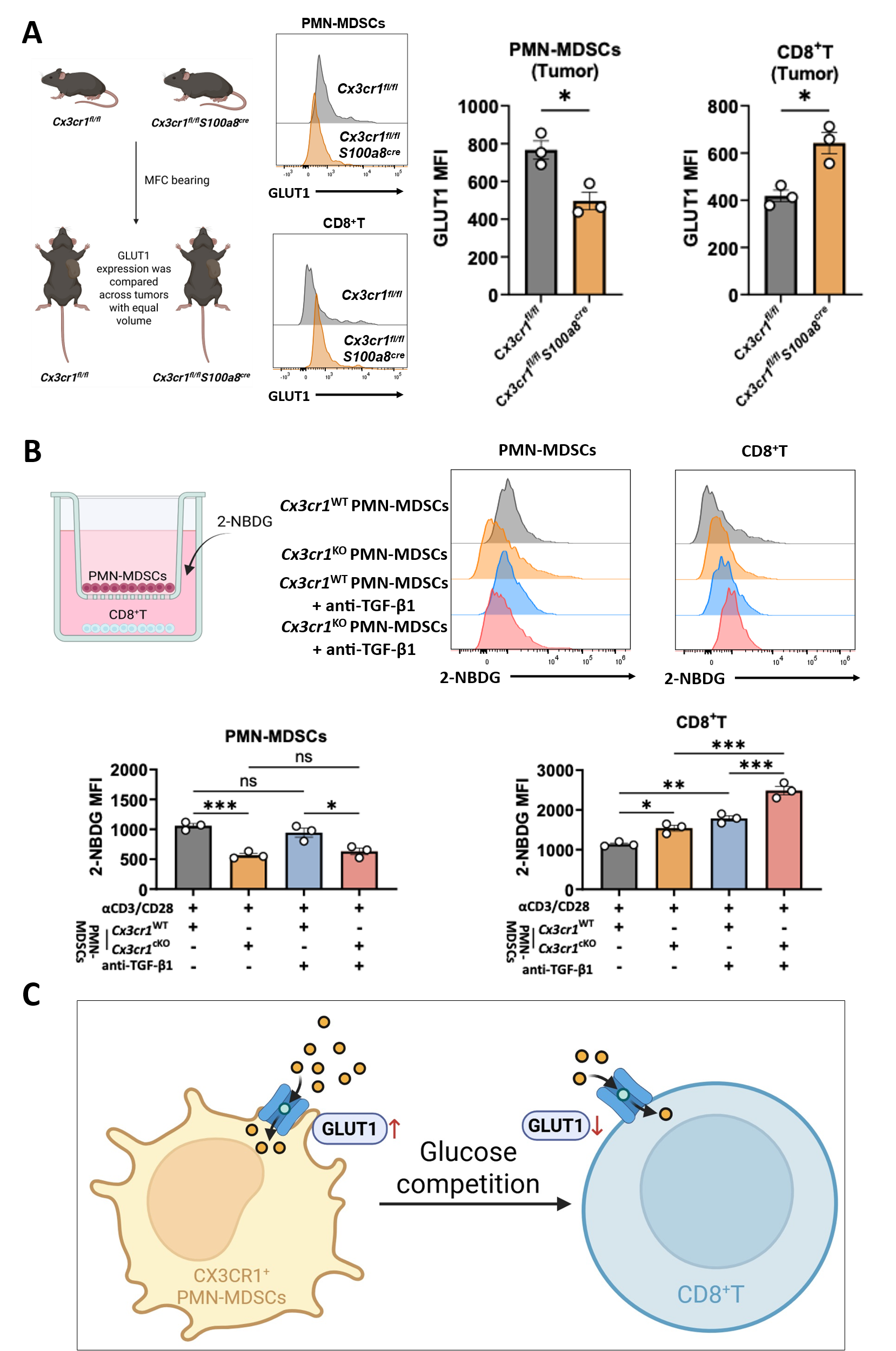
**

**Fig. S14: CX3CR1^+^ PMN-MDSCs compete glucose with CD8^+^ T cells in the TME.**

A. Experimental strategy for evaluating GLUT1 expression in PMN-MDSCs and CD8^+^T cell from *Cx3cr1^fl/fl^* and *Cx3cr1^fl/fl^S100a8^cre^* mice bearing MFC tumors. GLUT1 expression was compared across tumors with equal volume from the different genotypes.
B. Flow cytometry histograms showing GLUT1 expression in PMN-MDSCs and CD8^+^ T cells, with or without anti-TGF-β1 treatment in the co-culture system.
C. Schematic illustrating the competition for glucose between CX3CR1^+^ PMN-MDSCs and CD8^+^ T cells in the TME.

Data are presented as mean ± SEM for the bar charts. All groups had n = 3 biological replicates. Data of (A) were analyzed using Student’s t-test. Data of (B) were analyzed using one-way ANOVA followed by Bonferroni’s post hoc test. **P* < 0.05, ***P* < 0.01, ****P* < 0.005, *****P* < 0.0001, and ns for non-significant.

**Fig. S15**

**
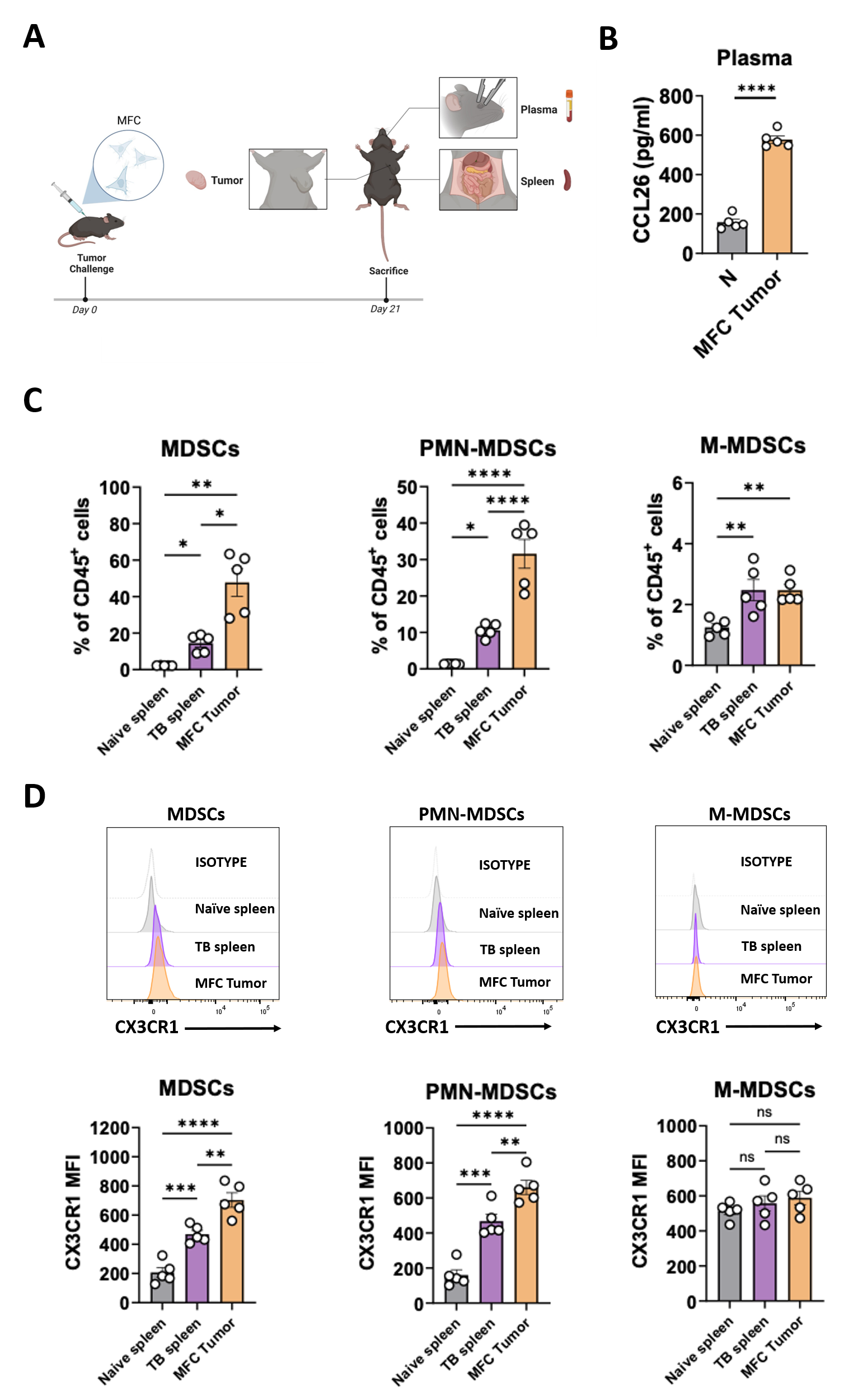
**

**Fig. S15: Plasma level of CCL26 and CX3CR1 expression on MDSCs exhibited upregulation in MFC tumor-bearing mice.**

A: Schematic illustration of the animal model construction.

B: Plasma level of CCL26 between MFC tumor-bearing mice and blank control group.

C: Frequencies of total MDSCs/CD45^+^ cells, PMN-MDSCs/CD45^+^ cells and M-MDSCs/CD45^+^ cells in spleen and tumor.

D: Quantitative mean fluorescence intensity (MFI) of CX3CR1 on total MDSCs, PMN-MDSCs and M-MDSCs in spleen and tumor.

Data are presented as mean ± SEM for the bar charts. All groups had n = 5 biological replicates. Data of (B) were analyzed using Student’s t-test. Data of (C) and (D) were analyzed using one-way ANOVA followed by Bonferroni’s post hoc test. **P* < 0.05, ***P* < 0.01, ****P* < 0.005, *****P* < 0.0001, and ns for non-significant.

**Fig. S16**

**
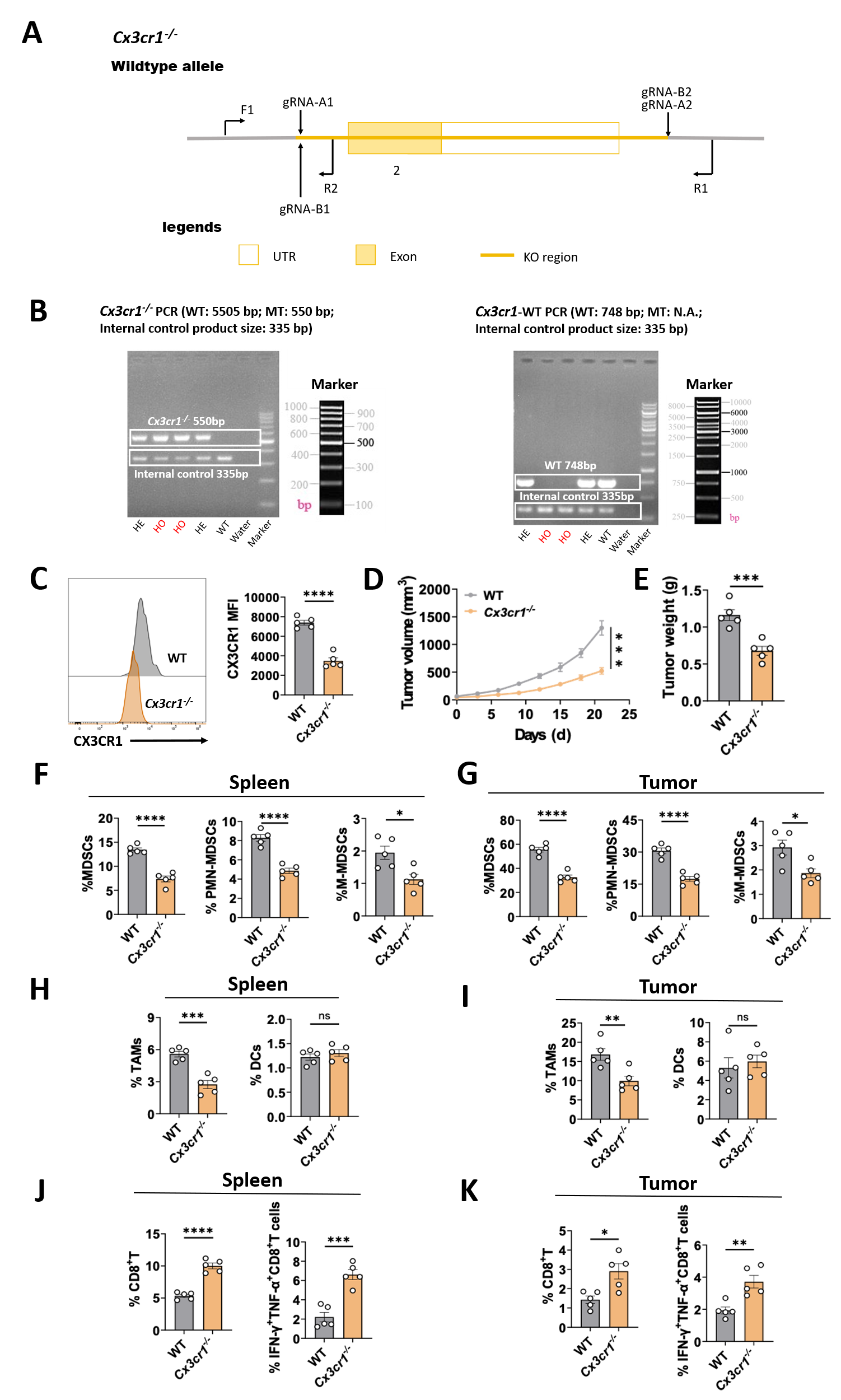
**

**Fig. S16: CX3CR1 knockout reduced MDSCs infiltration and improved CD8^+^ T cell anti-tumor response.**

A: Schematic illustration of the *Cx3cr1* knockout strategy.

B: Verification of mouse genotypes by agarose gel electrophoresis.

C: Tumor volume measurements in WT and *Cx3cr1^-/-^* mice.

D: Tumor weight measurements in WT and *Cx3cr1^-/-^* mice.

E: Representative flow cytometry plots showing the expression of CX3CR1 in PMN-MDSCs from the spleen of WT and *Cx3cr1^-/-^* mice.

F: Summary data of MDSCs and their subsets in the spleen of WT and *Cx3cr1^-/-^* mice.

G: Summary data of MDSCs and their subsets within the tumor of WT and *Cx3cr1^-/-^* mice.

H: Summary data of TAMs and DCs in the spleen of WT and *Cx3cr1^-/-^* mice.

I: Summary data of TAMs and DCs within the tumor of WT and *Cx3cr1^-/-^* mice.

J: Summary data of CD8^+^T cells producing dual cytokines (IFN-γ and TNF-α) in the spleen of WT and *Cx3cr1^-/-^* mice.

K: Summary data of CD8^+^T cells producing dual cytokines (IFN-γ and TNF-α) within the tumor of WT and *Cx3cr1^-/-^* mice.

Data are presented as mean ± SEM for the bar charts. All groups had n = 5 biological replicates. All data were analyzed using Student’s t-test. **P* < 0.05, ***P* < 0.01, ****P* < 0.005, *****P* < 0.0001, and ns for non-significant.

**Fig. S17**

**
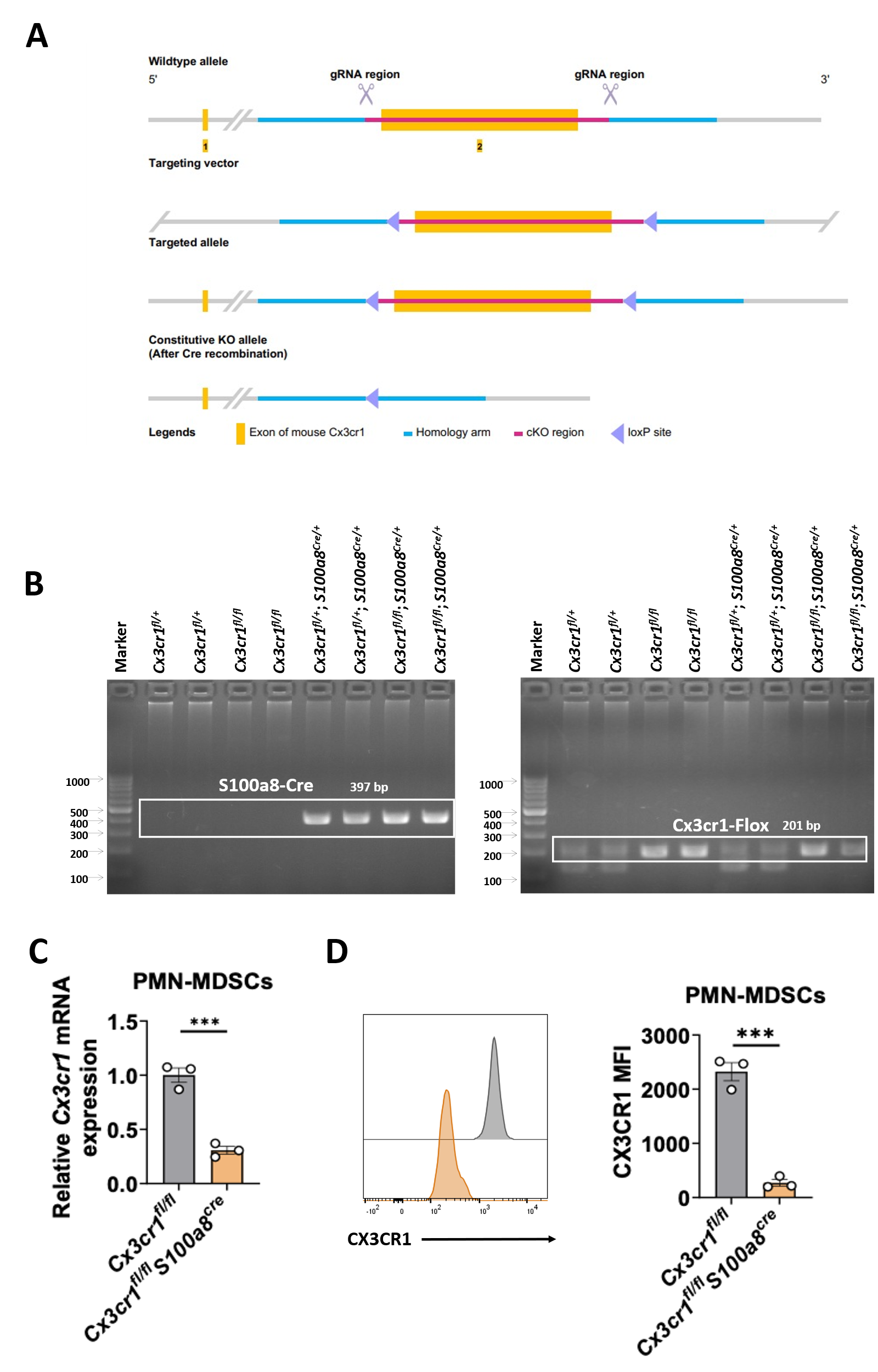
**

**Fig. S17: Verification of genotypes of PMN-MDSCs-specific CX3CR1 knockout mice by agarose gel electrophoresis.**

A: Strategy to generate *Cx3cr1^fl/fl^* mice.

B: Verification of mouse genotypes by agarose gel electrophoresis.

C: *Cx3cr1* mRNA expression in PMN-MDSCs from the spleens of *Cx3cr1^fl/fl^* and *Cx3cr1^fl/fl^S100a8^cre^* mice, as measured by qPCR.

D: CX3CR1 MFI in PMN-MDSCs from the spleens of *Cx3cr1^fl/fl^* and *Cx3cr1^fl/fl^S100a8^cre^* mice, as measured by flow cytometry.

Data are presented as mean ± SEM for the bar charts. All groups had n = 3 biological replicates. All data were analyzed using Student’s t-test. **P* < 0.05, ***P* < 0.01, ****P* < 0.005, *****P* < 0.0001, and ns for non-significant.

**Fig. S18**

**
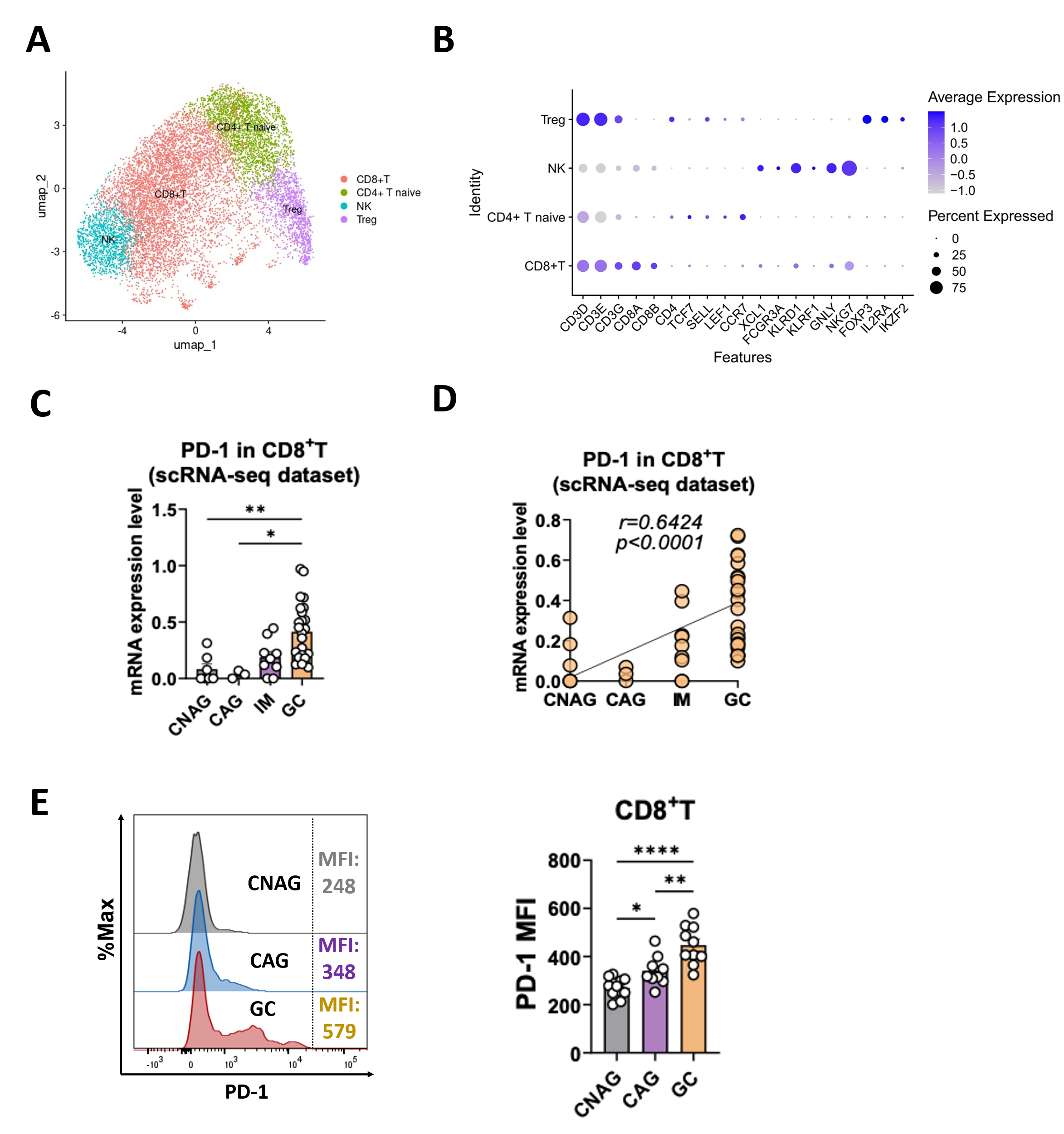
**

**Fig. S18: Identification of** **CD8^+^T cells and analysis of PD-1 on CD8^+^ T cells during stomach carcinogenesis.**

A: UMAP plot depicting the distribution of subtypes within T/NK cells. Data were derived from four scRNA-seq datasets (GEO: GSE134520, GSE183904, GSE150290; dbGaP: phs001818.v2), totaling n = 44 patients. Processing was performed in Seurat (v4.3.0) with quality control (200-6,000 genes per cell; mitochondrial RNA ≤ 10%), LogNormalize normalization, and selection of 3,000 highly variable genes, followed by PCA (first 20 components). Datasets were integrated with Harmony for batch correction. Analyses were restricted to the T/NK compartment; clustering (resolution r = 0.1) and UMAP were performed on the Harmony-corrected principal components using dimensions 1-20.

B: Dot plot of cell types markers within T/NK cells. Same datasets and preprocessing pipeline as in Fig. S18A. Dot size indicates the percentage of cells expressing each gene; color indicates average scaled expression.

C: Expression of PD-1 in CD8^+^ T cells during stomach carcinogenesis as assessed by scRNA-seq data across the CNAG (n = 7), CAG (n = 3), IM (n = 9), and GC (n = 25) groups.

D: Correlation between PD-1 expression in CD8^+^ T cells and pathological stages in stomach carcinogenesis based on scRNA-seq data from the CNAG (n = 7), CAG (n = 3), IM (n = 9), and GC (n = 25) groups.

E: Flow cytometry analysis of PD-1 expression in CD8^+^ T cells across different stages of stomach carcinogenesis, with n = 10 per group.

Data are presented as mean ± SEM for the bar chart. Data of (C) were compared using the Kruskal-Wallis test followed by Dunnett's post hoc test. Correlation analysis of (D) was performed using Spearman’s correlation. Data of (E) were analyzed using one-way ANOVA followed by Bonferroni’s post hoc test.

**P* < 0.05, ***P* < 0.01, ****P* < 0.005, *****P* < 0.0001, and ns for non-significant.

**Fig. S19**

**
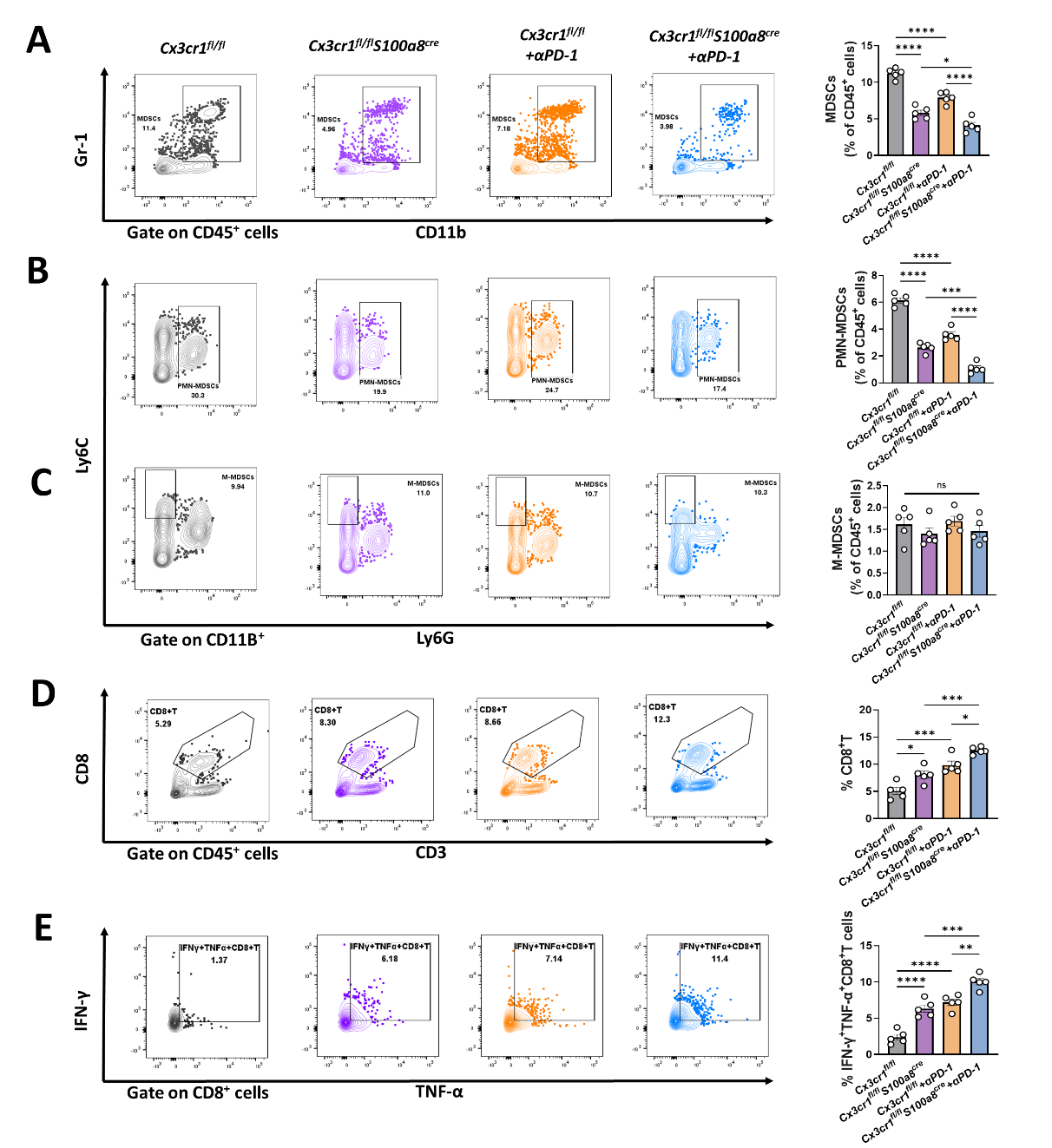
**

**Fig. S19: PMN-MDSCs-specific CX3CR1 knockout reduced PMN-MDSCs infiltration and improved CD8^+^ T cell anti-tumor response in spleens.**

A: Flow cytometry plots and corresponding quantitative analysis of MDSCs in spleens from mice of *Cx3cr1^fl/fl^*, *Cx3cr1^fl/fl^S100a8^cre^*, *Cx3cr1^fl/fl^* treated with αPD-1, and *Cx3cr1^fl/fl^S100a8^cre^* treated with αPD-1.

B: Flow cytometry plots and corresponding quantitative analysis of PMN-MDSCs in spleens from mice of *Cx3cr1^fl/fl^*, *Cx3cr1^fl/fl^S100a8^cre^*, *Cx3cr1^fl/fl^* treated with αPD-1, and *Cx3cr1^fl/fl^S100a8^cre^* treated with αPD-1.

C: Flow cytometry plots and corresponding quantitative analysis of M-MDSCs in spleens from mice of *Cx3cr1^fl/fl^*, *Cx3cr1^fl/fl^S100a8^cre^*, *Cx3cr1^fl/fl^* treated with αPD-1, and *Cx3cr1^fl/fl^S100a8^cre^* treated with αPD-1.

D: Flow cytometry plots and corresponding quantitative analysis of CD8^+^T cells in spleens from mice of *Cx3cr1^fl/fl^*, *Cx3cr1^fl/fl^S100a8^cre^*, *Cx3cr1^fl/fl^* treated with αPD-1, and *Cx3cr1^fl/fl^S100a8^cre^* treated with αPD-1.

E: Flow cytometry plots and corresponding quantitative analysis of IFN-γ^+^TNF-α^+^CD8^+^T cells in spleens from mice of *Cx3cr1^fl/fl^*, *Cx3cr1^fl/fl^S100a8^cre^*, *Cx3cr1^fl/fl^* treated with αPD-1, and *Cx3cr1^fl/fl^S100a8^cre^* treated with αPD-1.

Data are presented as mean ± SEM for the bar charts. All groups had n = 5 biological replicates. All data were analyzed using one-way ANOVA followed by Bonferroni’s post hoc test. **P* < 0.05, ***P* < 0.01, ****P* < 0.005, *****P* < 0.0001, and ns for non-significant.
